# Supplementary material for: Recognition of centromere‐specific histone Cse4 by the inner kinetochore Okp1‐Ame1 complex
Source: EMBO Rep. 2023 Nov 20;24(12):e57702. doi: 10.15252/embr.202357702 (PMC10702835; doi:10.15252/embr.202357702)
Supplement: Supplementary file 1 — Expanded View Figures PDF [file EMBR-24-e57702-s008.pdf]

Expanded View Figures

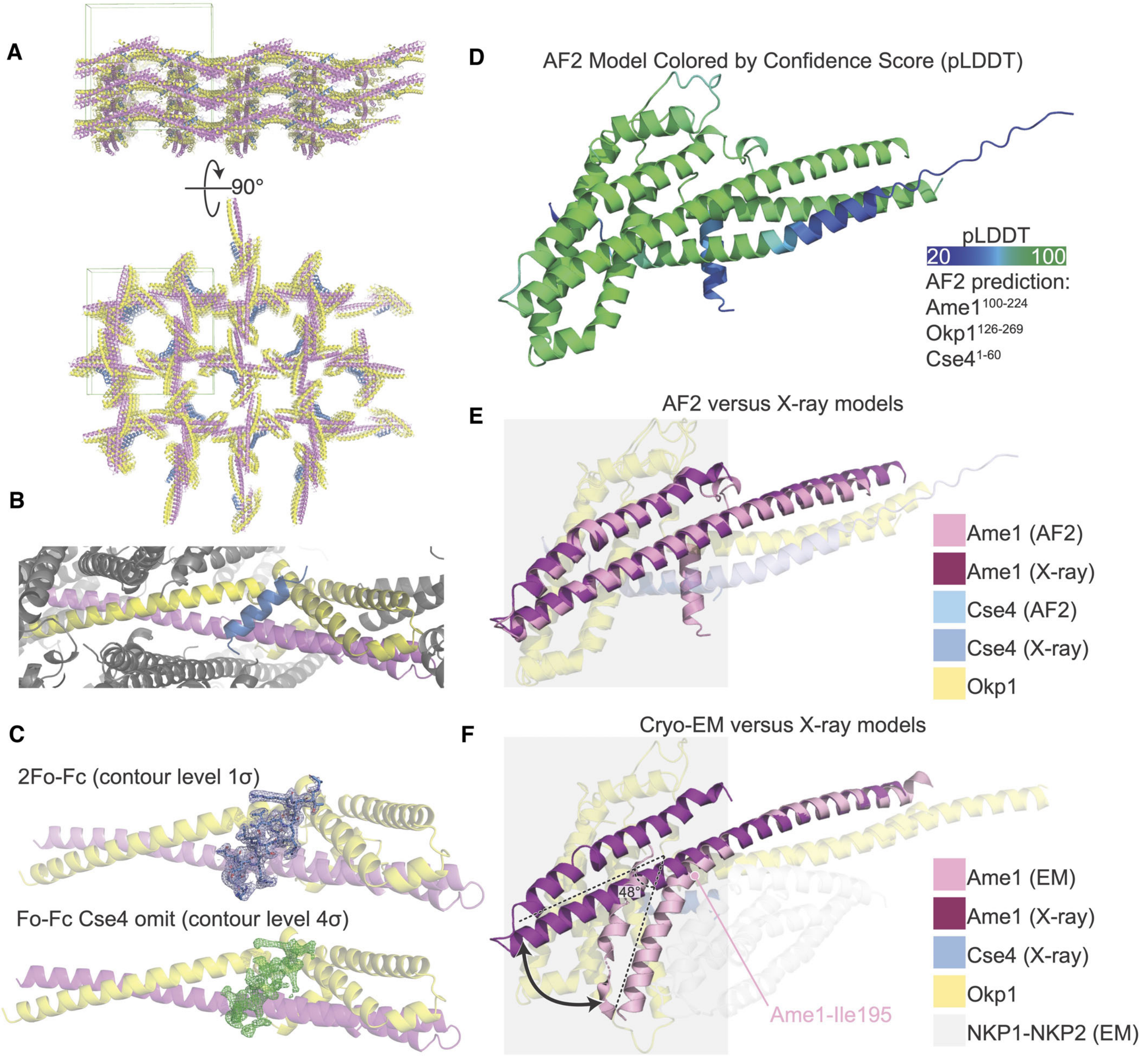

Figure EV1. Crystal structure of Okp1-Ame1-Cse4^{END} and flexibility at the Okp1-Ame1 head-coiled-coil joint and Nkp1-Nkp2 position.

A The crystal structure of Okp1-Ame1-Cse4 colored as in Fig 1. The green box shows the limits of a single unit cell.

B Close-up view of an individual biological protomer. Okp1-Ame1-Cse4 colored as in Fig 1. Neighboring protomers are colored gray.

C Cse4^{END} peptide density from the final refined model (top; 2Fo-Fc) and from the refined model lacking Cse4^{END} (bottom; Fo-Fc, Cse4 omitted).

D The structure of Okp1-Ame1-Cse4 as predicted by AlphaFold 2 (AF2) (Jumper *et al*, 2021). The model is colored according to confidence score (pLDDT) from low (blue) to high (green). The peptides used for prediction are given at right.

E Overlay of Okp1-Ame1-Cse4 from AF2 with the current Okp1-Ame1-Cse4 crystal structure (X-ray). Only Ame1 (magenta and pink) is shown as an opaque chain for clarity. The gray box marks the Okp1-Ame1 head domain.

F Overlay of the Okp1-Ame1-Cse4 structure from cryo-EM (EM) with the current crystal structure. The angle between the head and coiled coil shaft is indicated for the cryo-EM structure. Ame1-Leu195, which is the position at which Ame1 bends in the cryo-EM structure, is annotated. The Okp1-Ame1 head domain is marked as in panel E. Structures were aligned on the Okp1-Ame1 coiled coil shaft. The Nkp1-Nkp2 structure from cryo-EM (NKP1$^{2-76}$; NKP2$^{4-84}$) is shown as transparent gray chains.

© 2023 The Authors

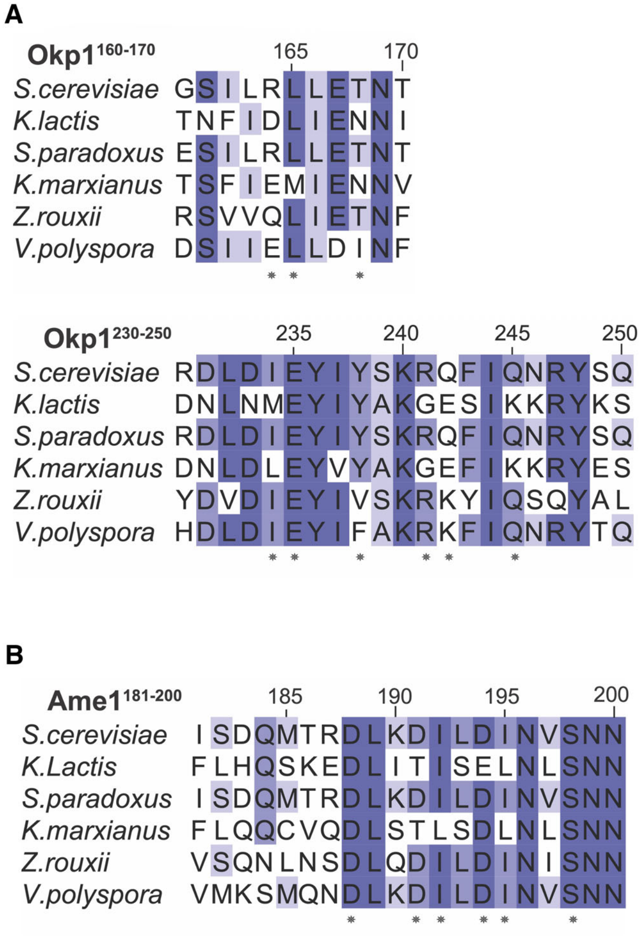

Figure EV2. Protein sequence alignments for Okp1 and Ame1 covering the Cse4^{END} contacts shown in Fig 2.

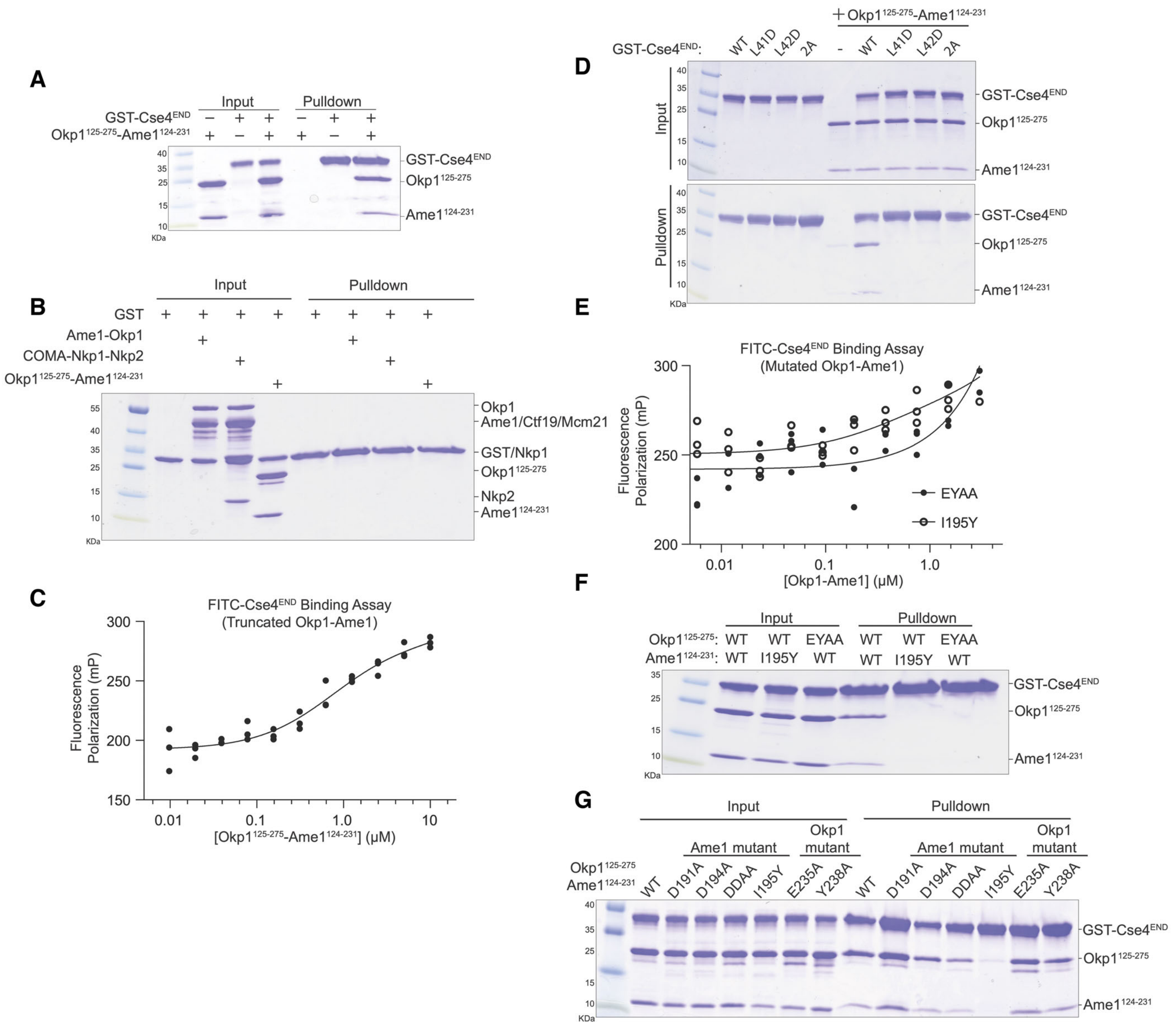

Figure EV3. Further biochemical characterization of the Okp1-Ame1-Cse4^{END} interaction.

A Pulldown assay showing binding between the truncated Okp1-Ame1 complex used for crystallography (Okp1$^{125\text{-}275I}$-Ame1$^{124\text{-}231}$) and GST-Cse4^{END}.

B Recombinant proteins used for pulldowns in Fig 3 were tested for their association with GST to determine the level of non-specific binding. Results of a GST pulldown assay are shown.

C The minimal Okp1-Ame1 complex used for crystallography was tested for its association with FITC-Cse4^{END} in a fluorescence polarization experiment. The measured dissociation constant is ~750 nM (see Materials and Methods; n = 3 independent experiments).

D GST-Cse4^{END} and its mutants were tested for Okp1-Ame1 binding.

E Full-length mutant Okp1-Ame1 complex (EYAA or I195Y as indicated) was tested for its association with FITC-Cse4^{END}.

F Various Okp1-Ame1 mutants (indicated above) were tested for binding to GST-Cse4^{END}.

G Various Okp1-Ame1 mutants were tested for Cse4^{END} binding as in panel F.

Source data are available online for this figure.

 © 2023 The Authors

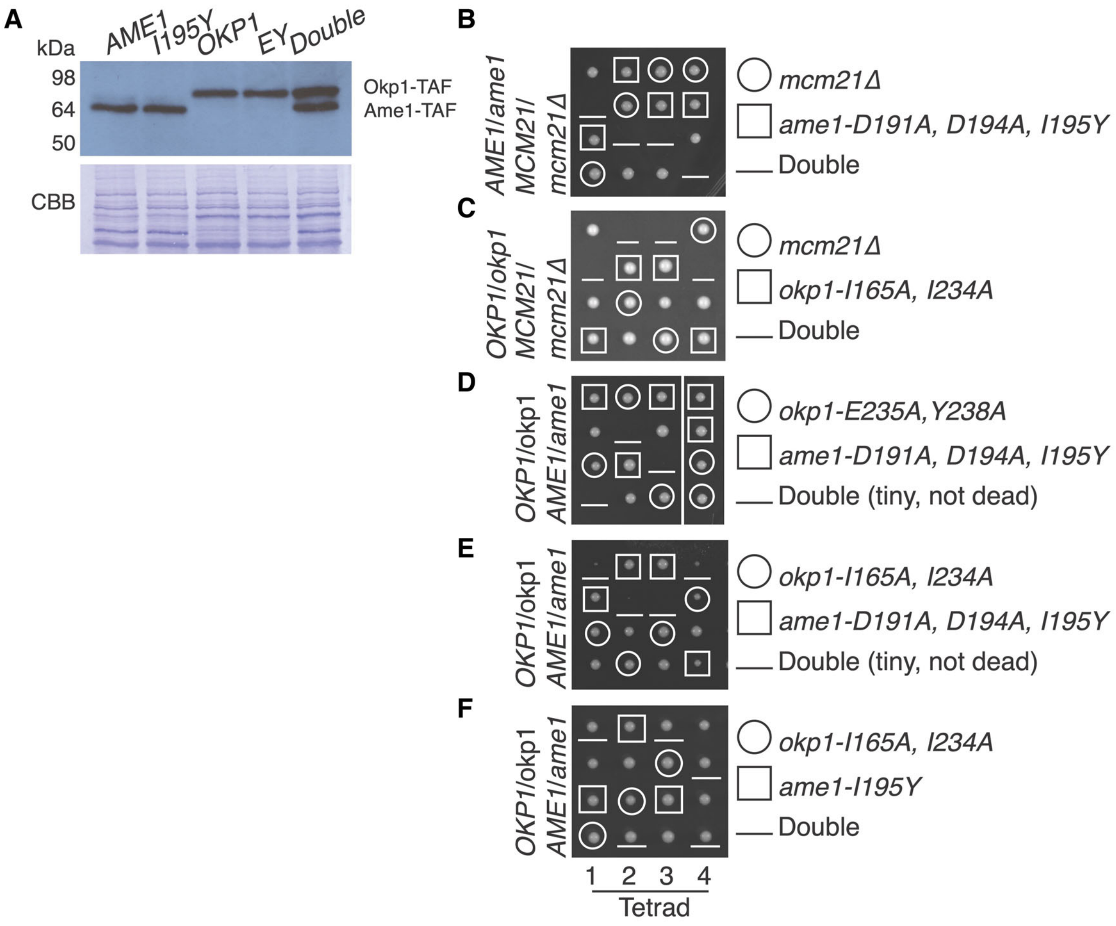

Figure EV4. ***In vivo* consequences of Okp1-Ame1 mutations.**

A Western blot showing expression of Ame1, Okp1, and their mutants in whole cell extracts (TAF – protein A-FLAG tag; anti-Protein A used for detection).

B–F Tetrad dissection results as in Fig 4B–D. The mutants tested and the resulting spore genotypes are shown at right.

Source data are available online for this figure.

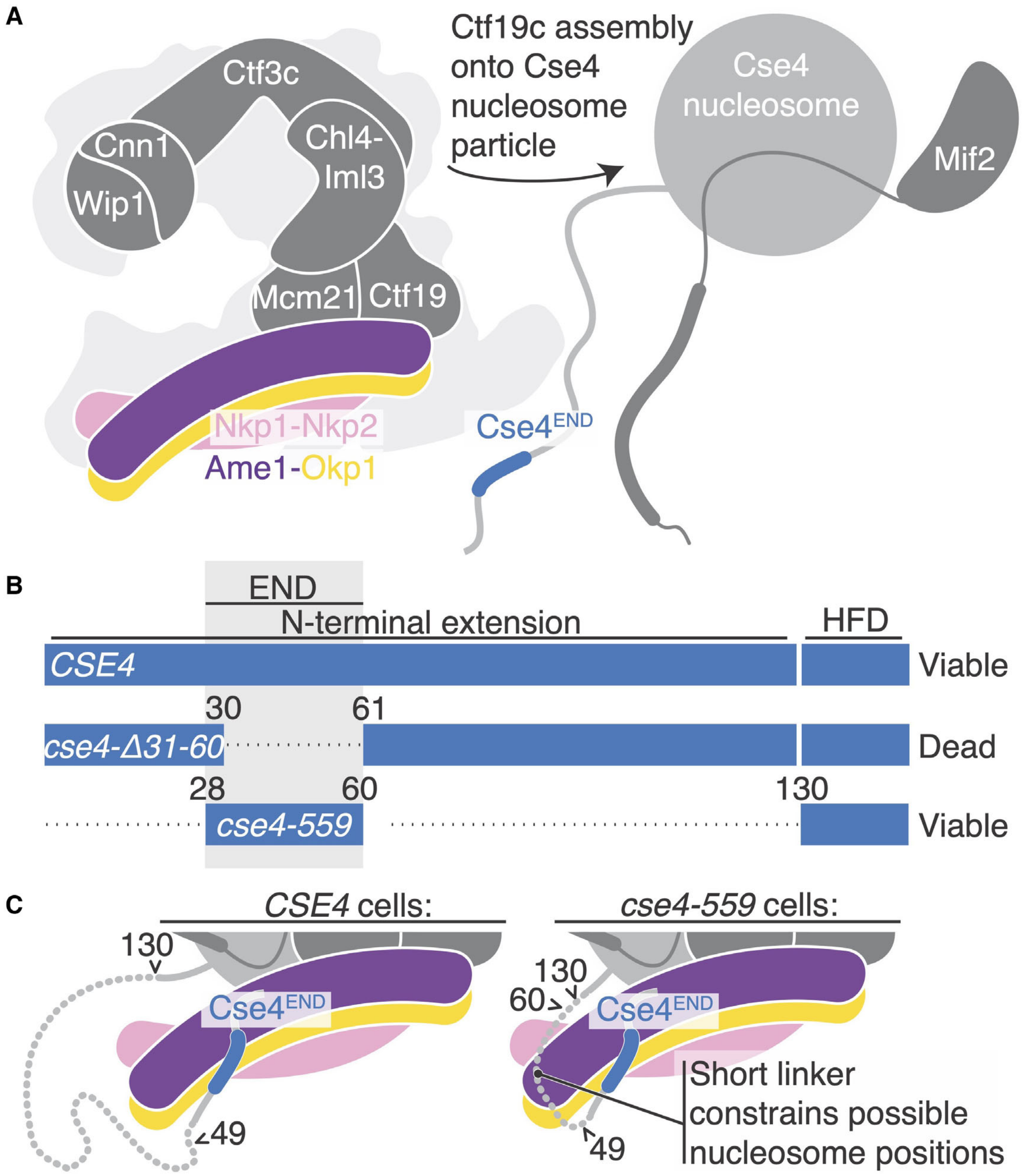

Figure EV5. Model for Cse4 nucleosome contact by the Ctf19c and relation to *cse4* alleles.

A Schematic showing Ctf19c assembly onto the Cse4-Mif2 complex. The assembly occurs via a largely unknown biochemical mechanism.

B *cse4* alleles (white text), their corresponding Cse4 proteins (blue bars), and their reported abilities to support cell viability (right). The alleles were reported by Chen *et al* (2000) and Fischbock-Halwachs *et al* (2019). The dotted lines indicate omitted fragments. The numbers correspond to full length Cse4. A gray box marks the boundaries of $Cse4^{END}$. HFD – histone fold domain.

C Structural view of the Cse4 N-terminal extension in *CSE4* (left) or *cse4-559* (right) cells. Arrows and nearby numbers point to Cse4 amino acid positions according to their numbering in the full-length protein.

 © 2023 The Authors
