# Supplementary material for: Recognition of centromere‐specific histone Cse4 by the inner kinetochore Okp1‐Ame1 complex
Source: EMBO Rep. 2023 Nov 20;24(12):e57702. doi: 10.15252/embr.202357702 (PMC10702835; doi:10.15252/embr.202357702)
Supplement: Supplementary file 2 — PDF+ [file EMBR-24-e57702-s004.pdf]

Report

SOURCE DATA · TRANSPARENT PROCESS · OPEN ACCESS

EMBO reports

Recognition of centromere-specific histone Cse4 by the inner kinetochore Okp1-Ame1 complex

Sunbin Deng[1], Jiaxi Cai[2], Stephen C Harrison[1], Huilin Zhou[2,3,*] & Stephen M Hinshaw[4,**]

Abstract

Successful mitosis depends on the timely establishment of correct chromosomal attachments to microtubules. The kinetochore, a modular multiprotein complex, mediates this connection by recognizing specialized chromatin containing a histone H3 variant called Cse4 in budding yeast and CENP-A in vertebrates. Structural features of the kinetochore that enable discrimination between Cse4/CENP-A and H3 have been identified in several species. How and when these contribute to centromere recognition and how they relate to the overall structure of the inner kinetochore are unsettled questions. More generally, this molecular recognition ensures that only one kinetochore is built on each chromatid and that this happens at the right place on the chromatin fiber. We have determined the crystal structure of a Cse4 peptide bound to the essential inner kinetochore Okp1-Ame1 heterodimer from budding yeast. The structure and related experiments show in detail an essential point of Cse4 contact and provide information about the arrangement of the inner kinetochore.

Keywords centromere; kinetochore; mitosis; X-ray crystallography
Subject Categories Cell Cycle; Structural Biology
DOI 10.15252/embr.202357702 | Received 21 June 2023 | Revised 30 October 2023 | Accepted 2 November 2023
EMBO Reports (2023) e57702

Introduction

Kinetochores assemble at centromeres by interacting with a variant histone H3 known as centromere protein A (CENP-A) in vertebrates and as Cse4 in *Saccharomyces cerevisiae* and other point-centromere yeast. CENP-A and Cse4 deviate from canonical histone H3 at residues interspersed in the histone core and in the sequence and length of the N-terminal segment (Fig 1A). Components of the assembled kinetochore that recognize these CENP-A- or Cse4-specific features include yeast Mif2 (CENP-C in vertebrates) and one or more subunits of the yeast Ctf19 complex (Ctf19c), which is homologous to the human CCAN (for Constitutive Centromere Associated Network). An important open question is how these factors "read" distinguishing features of Cse4 to restrict kinetochore assembly to centromeres.

The Cse4 N-terminal extension is much longer than its histone H3 counterpart (~140 and ~40 amino acid residues, respectively). Despite its length, a relatively short segment of the Cse4 N-terminal extension is necessary and sufficient for cell growth (residues 28–60, Fig 1A) (Chen *et al*, 2000; Ichikawa *et al*, 2018). Accordingly, this segment was named the Cse4 Essential N-terminal Domain (Cse4^{END}). Early yeast two-hybrid experiments suggested that Ctf19 binds Cse4^{END} (Chen *et al*, 2000). More recent biochemical reconstitution experiments showed that the Okp1-Ame1 heterodimer, which is a Ctf19c sub-module that binds the Ctf19 protein, is the direct binding partner of Cse4^{END} (Anedchenko *et al*, 2019; Fischbock-Halwachs *et al*, 2019; Hinshaw & Harrison, 2019).

Okp1 and Ame1 are the only Ctf19c components fully required for mitosis (Ortiz *et al*, 1999; Pot *et al*, 2005). They associate as an elongated heterodimer with a globular "head" and an extended alpha-helical coiled-coil "shaft." Loops in both chains interrupt the shaft and interact with Ctf19 and Mcm21 to make the COMA complex (Fig 1B) (Cheeseman *et al*, 2002; De Wulf *et al*, 2003; Hinshaw & Harrison, 2019). Nkp1 and Nkp2 bind along the length of the Okp1-Ame1 dimer, making a six-protein complex (Schmitzberger *et al*, 2017). Okp1, Ame1, Ctf19, and Mcm21 all have long, flexible N-terminal extensions. Only that of Ame1 is essential; it indirectly recruits the microtubule-binding Ndc80 complex to the kinetochore, thus enabling chromosomal contact with the mitotic spindle (Hornung *et al*, 2014).

Molecular recognition of Cse4 by the Okp1-Ame1 heterodimer has not been resolved, despite recent cryo-EM structures that show in detail nearly all protein contacts that contribute to inner kinetochore assembly in yeast (Yan *et al*, 2018, 2019; Hinshaw & Harrison, 2019, 2020; Guan *et al*, 2021; Dendooven *et al*, 2023). An experimental solution will constrain models for centromeric nucleosome binding by the Ctf19c. There are also several reported post-translational modifications of the Cse4 N-terminal extension that are thought to control kinetochore function (Hewawasam *et al*, 2010; Ranjitkar *et al*, 2010; Samel *et al*, 2012; Boeckmann *et al*, 2013; Hoffmann *et al*, 2018; Ohkuni *et al*, 2018; Anedchenko *et al*, 2019; Mishra *et al*, 2021). Cse4 binding to Okp1-Ame1 can be

1 Department of Biological Chemistry and Molecular Pharmacology, Harvard Medical School, and Howard Hughes Medical Institute, Boston, MA, USA
2 Department of Bioengineering, Jacobs School of Engineering, UCSD, San Diego, CA, USA
3 Department of Cellular and Molecular Medicine, School of Medicine, Moores Cancer Center, UCSD, San Diego, CA, USA
4 Stanford Cancer Institute, Stanford School of Medicine, Stanford, CA, USA
*Corresponding author. Tel: (858) 534-7808; E-mail: huzhou@health.ucsd.edu
**Corresponding author. Tel: (650)497-4248; E-mail: hinshaw@stanford.edu

© 2023 The Authors. Published under the terms of the CC BY 4.0 license

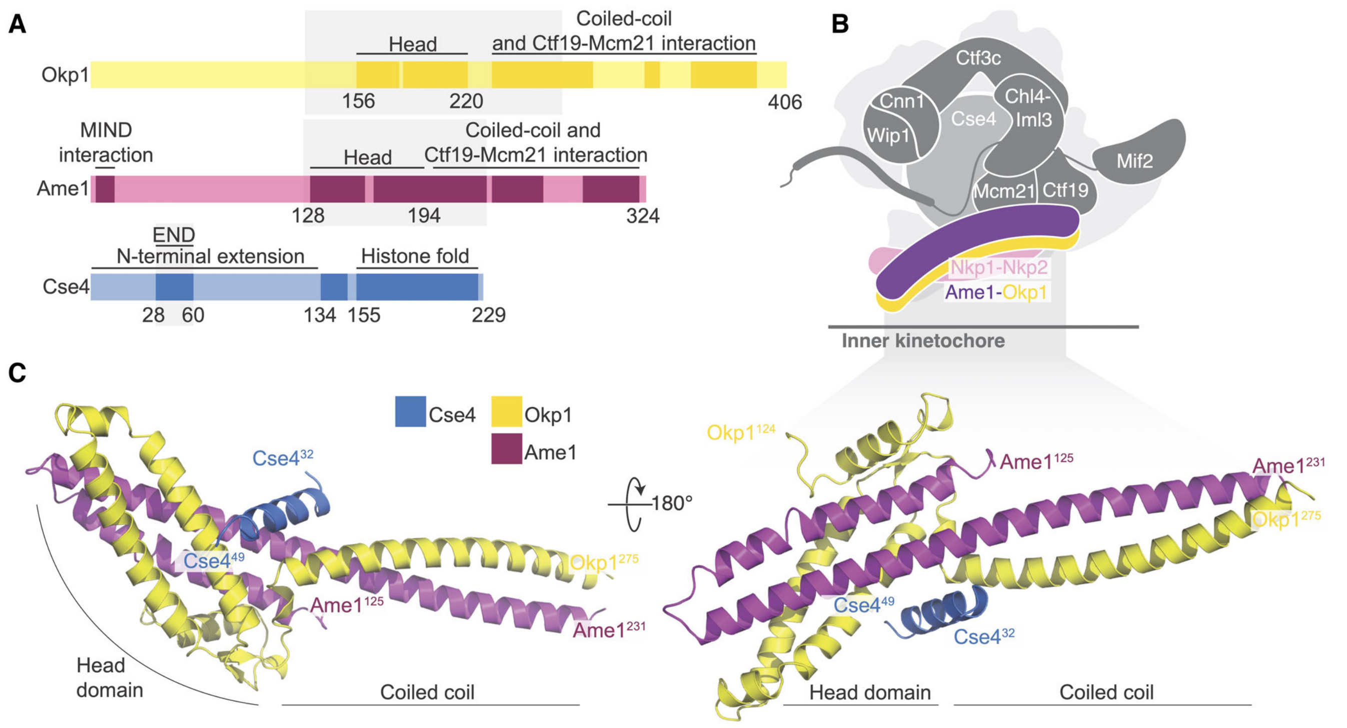

Figure 1. Crystal structure of the Okp1-Ame1-Cse4^{END} complex.

A Domain diagram showing the relevant regions of Okp1, Ame1, and Cse4. The shaded boxes demarcate the minimal Okp1-Ame1 peptides used for crystallography. The "Head" domains correspond to the previously described four-helix bundle (by analogy to the MIND complex; Dimitrova *et al*, 2016). Darker regions of the primary structure diagram indicate segments that are ordered in previously reported structures or that are known to bind partners. An N-terminal peptide of Ame1 connects to spindle microtubules indirectly via the MIND complex (Hornung *et al*, 2014).

B Schematic view of the yeast inner kinetochore (Ctf19c, Mif2, and Cse4) showing the position of Okp1-Ame1 (purple and yellow) and Nkp1-Nkp2 (pink). N-terminal extensions are omitted for clarity.

C Crystal structure of the Okp1-Ame1-Cse4^{END} complex (this work). Protein chains are colored as in panel A.

Source data are available online for this figure.

Table 1. Affinity measurements for Cse4^{END} binding determined by fluorescence polarization.

	Okp1-Ame1 version	K_D	r^2
Okp1-Ame1 (WT)	FL	152 (97–240) nM	0.9623
Okp1-Ame1 W(T) + 10 μM Nkp1-Nkp2	FL	133 (80–220) nM	0.9576
Okp1(EYAA)-Ame1	FL	> 3 μM	n.d.
Okp1-Ame1(I195Y)	FL	>> 3 μM	n.d.

Full-length Okp1-Ame1 complex was used. Equilibrium constant values are given with the asymmetric 95% confidence interval shown in parentheses (K_D – equilibrium dissociation constant, r^2 – goodness of fit, n.d. – not determined).

reconstituted *in vitro* (Anedchenko *et al*, 2019; Fischbock-Halwachs *et al*, 2019; Hinshaw & Harrison, 2019), but a published cryo-EM structure that includes Cse4 and the assembled Ctf19c did not show Cse4^{END} density (Yan *et al*, 2019). In our own efforts to solve this problem, we have attempted to determine the structure of the Ctf19c bound either to a reconstituted Cse4 nucleosome particle or to a minimal Cse4^{END} peptide, but the resulting maps do not show density corresponding to Cse4^{END}. In the current work, we have used X-ray crystallography to identify the Cse4^{END} binding site.

We show here that Cse4^{END} binds at the head-shaft junction of Okp1-Ame1. We determined a 1.8 Å resolution crystal structure of a truncated Okp1-Ame1 bound with a Cse4^{END} peptide to specify in detail the amino acid residues involved, and we have shown by binding experiments and yeast genetics that the interaction is functionally relevant in cells.

Results and Discussion

The crystal structure of Cse4^{END} with Okp1-Ame1

We determined the crystal structure of the Cse4^{END} peptide bound to a minimal Okp1-Ame1 heterodimer (Fig 1C). Crystals containing all three proteins, including the full Cse4^{END} sequence, grew in space group P $4_2 2_1 2$ (Fig EV1A and B) and yielded diffraction data to a minimum Bragg spacing of 1.8 Å. We determined the structure by molecular replacement (MR) as described in the Materials and Methods section and in Table EV1.

 © 2023 The Authors

In the crystal structure of the heterotrimeric complex, Cse4^{END} binds at the junction between the globular head of Okp1-Ame1 and the proximal part of the coiled-coil shaft (Figs 1C and EV1C). The ordered Cse4 residues in the crystal structure are mostly α-helical (residues 34–46). The Cse4^{END} position is essentially as predicted by AlphaFold 2 (Jumper *et al*, 2021; Dendooven *et al*, 2023 and this work; Fig EV1D and E). The Cse4^{END} binding site is on an external surface in structures of the assembled Ctf19c (Hinshaw & Harrison, 2019; Yan *et al*, 2019).

The orientation of the four-helix bundle that makes up the Okp1-Ame1 head domain differs from what we and others observed in cryo-EM reconstructions of the Ctf19c (Fig EV1F) (Hinshaw & Harrison, 2019; Yan *et al*, 2019). In the current structure, the Okp1-Ame1 head tilts away from the shaft. This is most evident for Ame1, where there is no discernable break in the extended helix that connects the shaft and head, whereas the same helix bends at Ame1-I195 in the previous Ctf19c structures.

Specific contacts that enable Cse4^{END}-Okp1-Ame1 binding

The three-way interface between Cse4, Okp1, and Ame1 has a hydrophobic core surrounded by polar interactions. The Cse4^{END} residues at the interface are conserved across point-centromere yeast, as are most of their partners in Okp1 and Ame1 (Figs 2A and EV2). Hydrophobic contacts between Cse4 and Okp1 include Cse4-L42 and Okp1-I234 (Fig 2B). Likewise, Cse4-L41 and -I34 contact Ame1 I195 (Fig 2C). Peripheral charged contacts include an electrostatic interaction between Cse4-R46 and Okp1-E235. Cse4-R37 makes complementary charge contacts with Ame1-D191 and -D194. Both contacts are conserved in yeast. This overall arrangement, with multiple charged contacts surrounding a hydrophobic core encompassing all three proteins, explains how a short Cse4^{END} peptide binds the Okp1-Ame1 dimer with an equilibrium dissociation constant of ~150 nM (see below).

Comparison with Cryo-EM structures of the Ctf19c (Hinshaw & Harrison, 2019; Yan *et al*, 2019) shows that Nkp1 and Nkp2 bind close to the Cse4^{END} binding site on Okp1-Ame1. Because the Okp1-Ame1 head domain is shifted in the crystal structure as described above, it is not clear from these comparisons whether Nkp1-Nkp2 would need to partly dissociate from Okp1-Ame1 to accommodate Cse4^{END} (Fig EV1F). Indeed, a recent cryo-EM structure of COMA-Nkp1-Nkp2 shows partial Nkp1-Nkp2 dissociation from Okp1-Ame1 is possible (Dendooven *et al*, 2023). We used pulldown assays to test whether Nkp1-Nkp2 and Cse4^{END} compete for Okp1-Ame1 binding. Consistent with published data (Anedchenko *et al*, 2019; Fischbock-Halwachs *et al*, 2019; Hinshaw & Harrison, 2019), a glutathione-S-transferase (GST) fusion of Cse4^{END} bound Okp1-Ame1 (Fig 3A). GST-Cse4^{END} bound the four-protein COMA complex and the six-protein COMA-Nkp1-Nkp2 complex equally well. Truncation of Okp1-Ame1 to the minimal heterodimeric complex used for crystallization (Okp1$^{125\text{-}275}$-Ame1$^{124\text{-}231}$) preserved GST-Cse4^{END} binding (Fig EV3A). Control pulldowns confirmed that none of the tested prey proteins bind GST (Fig EV3B).

To quantify Cse4^{END} binding to Okp1-Ame1, we created a fluorescence polarization assay using a fluorescent Cse4^{END} peptide (Fig 3B). An equilibrium dissociation constant of ~150 nM describes the binding event, and this value matches one previously reported (Anedchenko *et al*, 2019). Adding excess Nkp1-Nkp2 protein to the binding reaction did not change the dissociation constant, consistent with the pulldown results. Therefore, Cse4 can bind the assembled COMA-Nkp1-Nkp2 complex. Because Nkp1-Nkp2 do not displace Cse4^{END} from Okp1-Ame1, their inclusion cannot explain the inability of Cse4^{END} to bind the reconstituted Ctf19c in biochemical experiments (Dendooven *et al*, 2023).

We also measured Cse4 affinity for the truncated Okp1-Ame1 complex used for crystallography. The measured dissociation constant was ~750 nM (Fig EV3C). The ~5-fold higher value versus full-length Okp1-Ame1 could partly be due to the higher salt concentration required for the truncated Okp1-Ame1 sample.

Effects of mutations on affinity of Cse4^{END} for Okp1-Ame1

We tested the importance of residues at the interface between Cse4^{END} and Okp1-Ame1 using the biochemical assays described above. The *cse4-L41D*, *cse4-L42D*, and *cse4-L41,42A* (*cse4-2A*) mutations disrupt central hydrophobic interactions with Okp1-Ame1. The corresponding Cse4^{END} peptides failed to bind recombinant Okp1-Ame1 in the pulldown assay (Fig 3C). The same was true in a pulldown assay carried out with the minimal Okp1-Ame1 complex used for crystallography (Fig EV3D). These experiments confirm the importance of hydrophobic contacts at the core of the three-protein interface.

We used the crystal structure to create Okp1-Ame1 mutations that specifically interfere with Cse4^{END} binding. Among those tested, *ame1-I195Y* and *okp1-E235,Y238A* (*okp1-EYAA*) were the most potent disruptors of Cse4 binding. Both mutations prevented Cse4^{END} binding in the pulldown and fluorescence polarization assays (Figs 3D and EV3E). They also prevented Cse4^{END} binding when introduced into the minimal Okp1-Ame1 complex (Fig EV3F). In addition to these mutations, we tested the *ame1-D191A* and *-D194A* mutations, which prevent coordination of the conserved Cse4-R37 via hydrogen bonding interactions. Neither mutation perturbed Cse4^{END} binding, and the combination (*ame1-DDAA*) disrupted Cse4 binding in the pulldown assay, but only slightly (Fig EV3G).

Effects of Cse4^{END} binding mutants on cell growth

To test whether Cse4^{END} mutations that disrupt Okp1-Ame1 binding interfere with cell division, we used a plasmid shuffling assay in which a plasmid-borne complementing *CSE4* allele restores the viability of *cse4Δ* cells. Selection against the complementing allele reveals the phenotype associated with a *CSE4* test allele carried on a second plasmid. The *cse4-2A* test allele did not support cell growth, and the *cse4-L41A* (*cse4-1A*) test allele produced slower-growing cells than did the *CSE4* allele (Fig 4A). Therefore, Cse4^{END} hydrophobic residues required for Ame1-Okp1 binding are also required for cell viability.

We next tested whether Okp1-Ame1 residues that interact with Cse4^{END} are essential. To do so, we sporulated diploid cells heterozygous for the *ame1-I195Y* and *okp1-E235A,Y238A* mutations and examined their haploid progeny (Fig 4B–D). Neither mutation produced obvious dominant defects in the parental heterozygous cells, and the expression levels of these mutant proteins matched their wild type counterparts (Fig EV4A). Both mutations permitted cell growth in haploid spores but were lethal in combination with

© 2023 The Authors

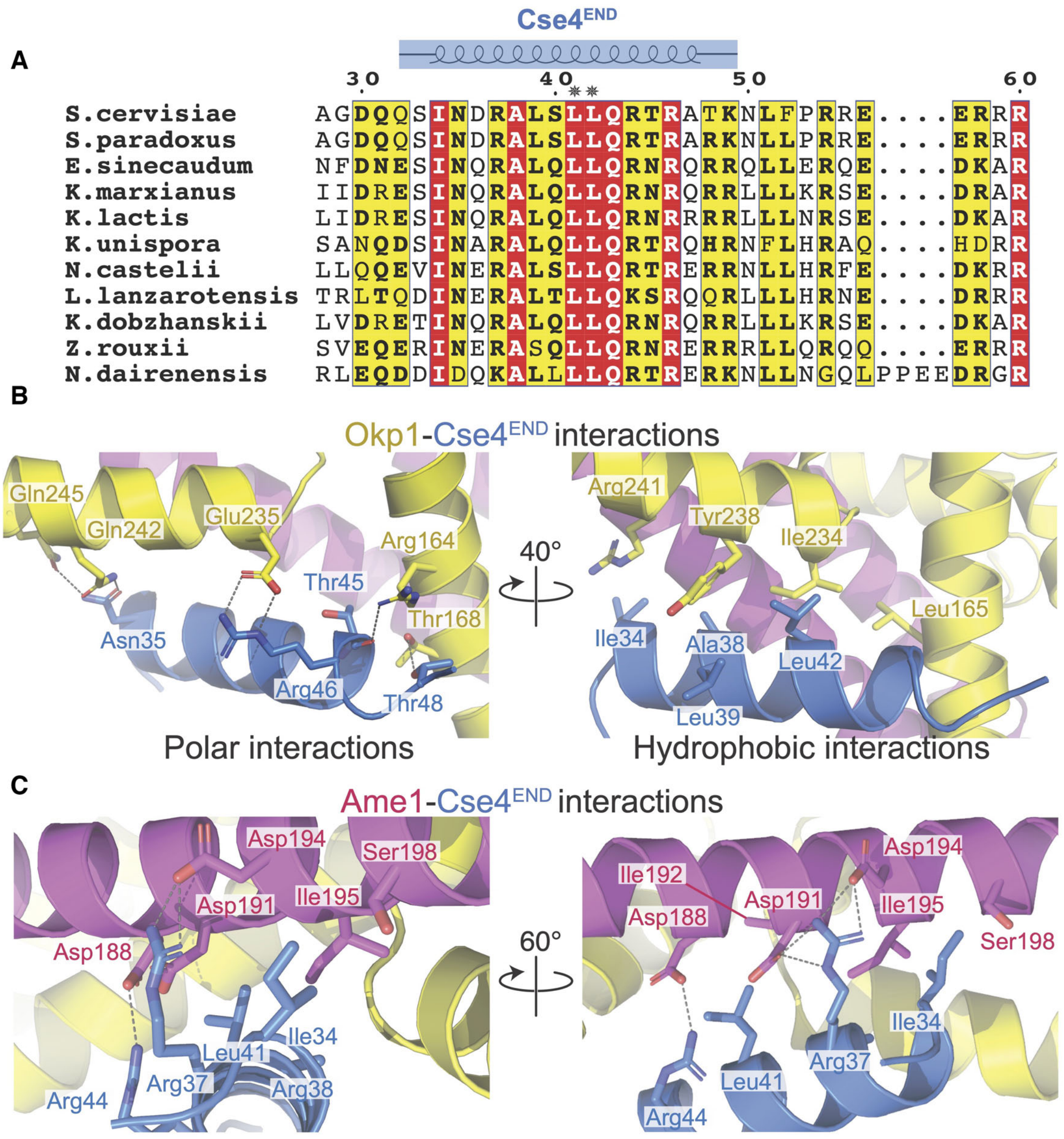

Figure 2. Contacts between Okp1-Ame1 and Cse4^{END}.

A Protein sequence alignment showing the Cse4^{END} peptide. Numbering corresponds to the *S. cerevisiae* Cse4 protein. Amino acid residues 28–60 are shown. The blue diagram above shows the residues visible in the crystal structure. Asterisks mark L41 and L42.

B Close-up views of the Okp1-Cse4 interface. Polar interactions are shown on the left. Hydrophobic contacts are shown on the right.

C Two views of the Ame1-Cse4 interface.

Source data are available online for this figure.

mcm21Δ (Fig 4C). This result matches the previous observation that non-lethal Cse4^{END} mutations are synthetic-lethal with Ctf19c mutations (e.g., *cse4-R37A chl4Δ* double mutant cells are inviable) (Samel *et al*, 2012; Anedchenko *et al*, 2019).

If *ame1-I195Y* and *okp1-E235A*, *Y238A* each support weak Cse4 binding below the detection limit of our biochemical assay, then introduction of both mutations might completely ablate the interaction, yielding inviable spores. To test this idea, we examined *okp1-E235A*, *Y238A ame1-I195Y* double mutants and found that these spores produced tiny colonies (Fig 4D). We carried out the same experiments for the *ame1-D191A*, *D194A*, *I195Y* and *okp1-I165A*, *I234A* mutations and observed the same set of double mutant phenotypes in progeny spores (Fig EV4B–F). Therefore, the Cse4 binding site on Okp1-Ame1 is required for cell viability. As

 © 2023 The Authors

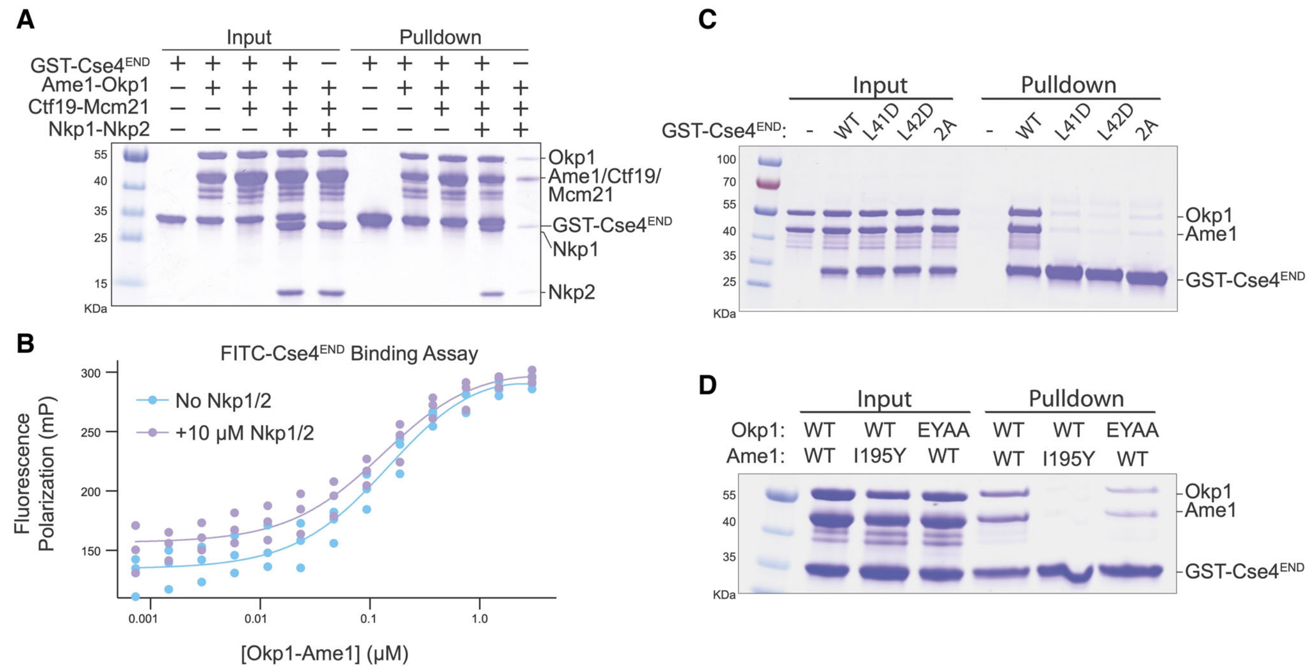

Figure 3. Biochemical investigation of Okp1-Ame1-Cse4^{END} binding and disruptive point mutations.

A GST pulldown assay for Cse4 binding. The indicated COMA complex proteins were tested for their association with immobilized GST-Cse4^{END}.

B Fluorescence polarization assay for quantification of Cse4 binding affinities (n = 3 independent replicates). FITC-labeled Cse4^{END} peptide was incubated with increasing concentrations of the indicated protein complexes. See also Table 1.

C, D GST pulldown assays as in panel A. Cse4^{END} and its mutants (L41D, L42D, or L41,42A) were tested in panel C. The indicated Okp1-Ame1 complexes were tested for their association with GST-Cse4^{END} in panel D.

Source data are available online for this figure.

demonstrated by the synthetic lethality when combined with *mcm21Δ*, an intact Ctf19c can compensate for partial disruption of the Cse4 binding site *in vivo*.

We have determined the crystal structure of an essential segment of Cse4 bound to the inner kinetochore proteins Okp1 and Ame1. This segment, Cse4^{END}, binds the junction between the Okp1-Ame1 coiled-coil shaft and head domains. Minimal Okp1-Ame1 mutations that disrupt Cse4 binding (this work) or MIND binding (Hornung *et al*, 2014), are lethal, indicating that the main essential function of Okp1-Ame1 is to connect centromeric DNA (via Cse4) to spindle microtubules (via MIND-Ndc80). Force transmission through these connections has been reconstituted in a minimal biochemical system (Hamilton *et al*, 2020). Whether this axis is the essential conduit for the force that moves chromosomes *in vivo* or if its essential function is regulatory remains to be seen.

Contacts between Cse4^{END} and Okp1-Ame1 seen in the crystal structure clarify observations from genetics experiments. The *cse4-R37A* mutation weakens Cse4-Okp1 binding and is lethal in genetic backgrounds with compromised kinetochores (e.g., *mcm21Δ*) (Anedchenko *et al*, 2019). Charge complementarity between Cse4-R37 and Ame1-D191/194 provides a structural basis for this finding. The *okp1-R164C* mutation suppresses lethality in *cse4-R37A mcm21Δ* cells (Anedchenko *et al*, 2019). The mutant Okp1 cysteine would have a set of non-polar contacts with T45 and T48 of Cse4, potentially compensating for the loss of a salt bridge between Cse4 R37 and Ame1 D191/194. Cse4 lysine acetylation also regulates Okp1-Ame1 binding, but the acetylated residue (Cse4-K49) is not visible in the crystal structure, preventing detailed analysis of this phenotype. Identification of Ame1 point mutations that prevent Cse4-R37 recognition (*ame1-D191A,D194A*) provides a tool useful for studying the function of Cse4 arginine methylation.

The Cse4-Okp1-Ame1 structure prompted us to revisit previous models for centromeric nucleosome recognition (Fig EV5). An initial Ctf19c structure in the absence of the Cse4 nucleosome suggested a straightforward docking model for Ctf19c-Cse4 binding, with which the observed position of Cse4^{END} in the current structure is compatible (Hinshaw & Harrison, 2019). This model requires repositioning of the Ctf3c-Cnn1-Wip1 complex and the Okp1-Ame1 head domains, along with partial DNA unwrapping from the nucleosome itself. Flexibility of the Ctf3 module and partial DNA unwrapping have been observed (Hinshaw & Harrison, 2020; Migl *et al*, 2020; Dendooven *et al*, 2023), and we report that the Okp1-Ame1 head domain is indeed mobile with respect to the adjacent coiled-coil shaft.

More recent cryo-EM structures of the yeast inner kinetochore (Yan *et al*, 2019; Dendooven *et al*, 2023) have raised the possibility of alternative Ctf19c-nucleosome poses. These rely principally on contact between Ctf19c proteins and the DNA phosphate backbone. In one case, the Cbf1 and Cep3 proteins contribute DNA sequence specificity (Dendooven *et al*, 2023). In these published structures, a ~60 Å peptide linker would be required to connect the Cse4 histone core to the Cse4^{END} peptide. This contradicts genetic evidence, which shows that fusion of the Cse4^{END} segment directly to the

© 2023 The Authors

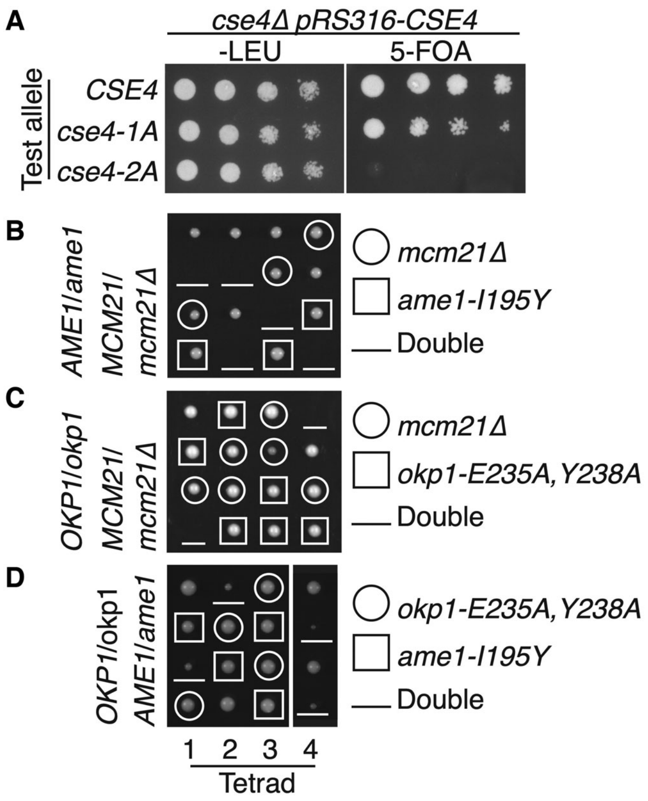

Figure 4. Cellular fitness of yeast with disrupted Cse4^{END} binding.

A Growth of *cse4* mutants. Loss of a complementing *CSE4* plasmid (*pRS316-CSE4*) was induced by growth on 5-FOA. Test *cse4* alleles are indicated at left (*cse4-1A* – L41A; *cse4-2A* – L41,42A).

B–D Haploid progeny from sporulation of the indicated heterozygous diploid strain (left). Spore genotypes and mutant alleles are given at right. Meiotic products from each tetrad are arranged vertically.

Source data are available online for this figure.

histone core (at residue 130) supports cell viability at a level indistinguishable from that of a wild type *CSE4* allele (Fig EV5B and C) (Chen *et al*, 2000). Dendooven *et al* have proposed an alternative assembly, in which an extra and presumably essential COMA complex orbits the Ctf19c and attaches through Cse4^{END} to satisfy this discrepancy (Dendooven *et al*, 2023), but there is at present no evidence for such an arrangement. Indeed, it is unclear what physiological context might support such an assembly, since the imaged samples were generated by mixing recombinant protein complexes without regard for cell cycle state, a known regulator of kinetochore assembly. Considering the existing structural data, we suggest two possibilities: (i) the heterotrimeric contacts we have described serve a cell cycle-specific function and do not occur in the fully assembled Ctf19c or (ii) the published Ctf19c-Cse4 structures do not recapitulate the physiologically relevant positions of the centromeric histone proteins and of the centromere itself.

This work serves to re-emphasize the question, what distinguishes the structure of the assembled Ctf19c, which is known, from its structure when bound to the centromeric nucleosome? There are likely several answers to this question, depending on cell cycle context. Neither the published Ctf19c-Cse4 structures nor our own unpublished structures, in which we have attempted to visualize just the Cse4^{END} peptide bound to the assembled Ctf19c, show any discernable Cse4^{END} density. That Cse4^{END} binds recombinant COMA but not the assembled Ctf19c suggests that a structural switch accompanies nucleosome engagement *in vivo*. The altered orientation of the Okp1-Ame1 head domain in the current structure is one candidate for such a switch. Relevant post-translational modifications of Ctf19c proteins, Cse4, and Mif2 have all been reported (Boeckmann *et al*, 2013; de Albuquerque *et al*, 2016; Anedchenko *et al*, 2019; Hinshaw *et al*, 2023). Mif2 is of particular interest; it is heavily phosphorylated, and any models of kinetochore assembly must account for this and its essentiality *in vivo*. Overall, it will be important to determine which modifications are required for inner kinetochore assembly and under what cell cycle conditions they contribute.

Materials and Methods

Protein expression and purification

Heterodimers of full-length Okp1-Ame1, Ctf19-Mcm21, and Nkp1-Nkp2 were expressed and purified in *E.coli* as previously described (Hinshaw & Harrison, 2019). Briefly, transformed cells were grown to an OD_{600} of 0.5 at 37°C in 2XYT media with antibiotic selection, induced with 0.5 mM of Isopropyl β-D-1-thiogalactopyranoside (IPTG) at 18°C for ~16 h, and harvested by centrifugation for 20 min at 4000 rpm. Pelleted cells were lysed by sonication in the lysis buffer containing 25 mM HEPES, pH 7.5, 800 mM NaCl, 10 mM imidazole,1 mM tris(2-carboxyethyl)phosphine (TCEP), 0.1 mg/ml PMSF, 5 mM β-mercaptoethanol, 1 mM PMSF, 1 μg/ml pepstatin, 1 μg/ml aprotinin, 1 μg/ml leupeptin, 30 μg/ml DNase I, and an EDTA-free protease inhibitor tablet (Roche, cOmplete™). After sonication, lysate clarified by centrifugation was passed over TALON Metal Affinity Resin (Takara), washed with 10 column volumes (CV) of lysis buffer, and eluted with 400 mM imidazole. The eluate was further purified on a HiTrap SP or Q ion-exchange column (Cytiva). The peak fractions were pooled for gel filtration in 20 mM Tris, pH 8.5, 200 mM NaCl, 1 mM TCEP. Peak fractions were collected, concentrated, snap-frozen, and stored at −80°C until use.

For pulldown assays, a DNA fragment coding for Cse4 residues 28–60 (Cse4^{END}) was cloned into a pLIC vector coding for a TEV-cleavable N-terminal Glutathione S-transferase tag and transformed into Rosetta™ 2 (pLysS) competent cells (Sigma-Aldrich). GST-Cse4^{END} was purified as described above, using Pierce Glutathione Agarose (Thermo Scientific) and eluting with L-Glutathione.

To find a minimal dimeric construct of Okp1-Ame1 that could bind Cse4, we cloned various truncated constructs of Okp1 and Ame1 genes into the pet-Duet bacterial expression vector, with a poly-histidine tag and tobacco etch virus (TEV) protease site at the N terminus of Okp1 and no tag on Ame1. We found that Okp1$^{125\text{-}275}$ – Ame1$^{124\text{-}231}$ can be expressed in Rosetta™ 2 (pLysS) competent cells (Sigma-Aldrich) and purified as described above, except that the N-His_6 tag on Okp1 was cleaved with TEV protease at 30°C for 1 h before the ion-exchange step. Final proteins after gel filtration were concentrated to ~15 mg/ml, snap frozen, and stored in 100 mM HEPES, pH 7.5, 300 mM ammonium sulfate, and 1 mM TCEP, at −80°C until use.

 © 2023 The Authors

Pulldown assays

GST-Cse4$^{28-60}$ was mixed with Okp1-Ame1, COMA, COMA-Nkp1-Nkp2, or Okp1$^{125-275}$ – Ame1$^{124-231}$ and the mixed samples were incubated with glutathione agarose resin at 4°C for 1 h in binding buffer containing 20 mM Tris, pH 8.5, 200 mM NaCl and 1 mM TCEP. Resin was washed at least three times with the same buffer to remove unbound protein, and bound protein then eluted with glutathione. Samples of input and eluate were collected for analysis by SDS–PAGE.

Fluorescence polarization assays

FITC-Cse4$^{28-60}$ peptide was synthesized by the Tufts University Core Facility. 20 nM of FITC-Cse4$^{28-60}$ peptide was used in all reactions, and Okp1-Ame1 concentrations were varied to determine the dissociation constant (K_D), in reaction buffer containing 100 mM HEPES, pH 7.5, 200 mM NaCl, 1 mM TCEP (full-length Okp1-Ame1 complex) or reaction buffer with 600 mM NaCl (truncated Okp1-Ame1 complex). The reactions were analyzed after incubating for 30 min at room temperature. Readings were recorded with a Perkin Elmer EnVision (ICCB-Longwood Screening Facility, Harvard University), and each curve was repeated in triplicate. EnVision data collection protocols were optimized separately for each titrated complex (Okp1-Ame1 full-length or its mutants, Okp1-Ame1 with Nkp1/2, and truncated Okp1-Ame1). GraphPad Prism was used for all data fitting (one site – total binding model). The 20 μM Okp1-Ame1 data points were eliminated for the truncated Okp1-Ame1 dataset due to high apparent non-specific binding.

Protein crystallization and diffraction data collection

Purified Okp1$^{125-275}$ – Ame1$^{124-231}$ was mixed with peptide Cse4$^{28-60}$ (synthesized at the Tufts University Core facility) in a ratio of 1: 1.3 and diluted to 9 mg/ml. The best crystals were grown within ~3 days by hanging drop vapor diffusion at 18°C against reservoir solution containing 19% PEG 8000, 0.55 M lithium sulfate and a 1:1 sample:well drop ratio. Crystals were transferred into mother liquor supplemented with 25% glycerol and flash-frozen in liquid nitrogen. Diffraction data to 1.8 Å minimum Bragg spacing were collected on beamline 24-ID-E at the Advanced Photon Source (Argonne National Laboratory). The complex crystallized in space group P 4_2 2_1 2 (a = 154.52, b = 154.52, c = 37.18). Data collection statistics are in Table EV1.

Structure determination

X-ray diffraction data processing was carried out with xia2 (https://xia2.github.io/index.html) using DIALS (Winter *et al*, 2022) and Aimless (Evans & Murshudov, 2013). The Okp1$^{162-275}$ – Ame1$^{124-231}$ structure from the yeast the Ctf19c/CCAN structure (PDB: 6NUW) failed to yield a molecular replacement solution in Phenix (Adams *et al*, 2010), but the AlphaFold2 (Jumper *et al*, 2021) prediction for Okp1$^{125-275}$ – Ame1$^{124-231}$ yielded a robust solution with clear density for the Cse4 peptide. Model building was carried out in Coot (Emsley *et al*, 2010) and refined in Phenix (Adams *et al*, 2010). Coordinates and diffraction data have been deposited in the protein data bank (PDB ID: 8T0P). Refinement statistics are in Table EV1.

Construction of plasmids and yeast strains

Centromeric plasmids (pRS315 and pRS316) were linearized by NotI and repaired by PCR products of Cse4 (ChrXI: 345945-347290), Ame1 (ChrII: 646026-647638) and Okp1 (ChrVII: 853524-855435) flanked by 34 bp homology via homologous recombination in yeast using standard yeast transformation method. Plasmids were rescued from successful transformants via electroporation, sequenced, and then used to make other plasmid derivatives. These plasmid derivatives, including insertion of 3xFlag, TAF tag (3xFlag-TEV-ProteinA), KanMX6 marker and later point mutants, were generated by combining PCR fragments with overlapping homology regions (> 33 bp), via homologous recombination in yeast. Specifically, a 3xFlag tag was inserted at an internal XbaI site in Cse4 (Wisniewski *et al*, 2014) and then a KanMX6 marker was inserted in the promoter region of Cse4 (ChrXI: 347222) to generate HZE2719. A TAF tag followed by KanMX marker was appended to the C-terminus of Ame1 (HZE2663) or Okp1 (HZE3198). These plasmids were then used to generate various point mutants as indicated.

A diploid of W303 strain (HZY1079) was used to generate a heterozygous deletion of Cse4, Ame1 or Okp1 using a natMX6 marker. After introducing pRS316-Cse4, pRS316-Ame1, or pRS316-Okp1, the transformed cells were sporulated to obtain the haploid *cse4*, *ame1* and *okp1* null mutants that were kept alive by their respective complementing plasmids. PCR products of *ame1* and *okp1* point mutants (with C-terminal TAF tag and G418 marker) were amplified from the mutant plasmids using a high-fidelity DNA polymerase (SuperFi, Invitrogen) and transformed into the respective haploid *ame1* and *okp1* null mutants. Correct integrations were identified by resistance to G418 and 5-Fluoroorotic acid (5-FOA), and nourseothricin-sensitivity, and confirmed by sequencing the genomic DNA of Ame1 and Okp1. Standard yeast genetic methods, including mating, sporulation, and dissection with a Singer dissection microscope, were used to construct various double mutants, as described by Lichten (2014). Plates were incubated at 30°C for 2 or 3 days before taking images using a BioRad ChemiDoc MP imaging system. The spores were identified based on the selection markers used to create each mutant. Yeast strains used are summarized in Table EV2. Plasmids used are summarized in Table EV3.

Plasmid shuffling using URA3/5-FOA

Haploid strains *cse4Δ::natMX6 pRS316-Cse4* (HZY2970), were transformed with the indicated plasmids bearing *cse4* mutants. Successful transformants were grown up in Sc-Leu media to an OD_{600} ~ 1, normalized based on cell density, and then spotted on the indicated plates following 5-fold serial dilutions. 5-fluoroorotic acid (Sc supplemented with 0.1% 5-FOA treatment removes the complementing *URA* plasmid), exposing the phenotypes of *cse4* mutants. Plates were incubated at 30°C for 2 or 3 days before taking images using a BioRad ChemiDoc MP imaging system.

Data availability

Coordinates of the structure described in this article have been deposited in the PDB with accession number (PDB ID: 8T0P; https://www.rcsb.org/structure/8T0P).

© 2023 The Authors

Expanded View for this article is available online.

Acknowledgements

We thank Cindy Zhang for technical assistance. We thank the ICCB-Longwood Screening Facility (Harvard Medical School) for help collecting fluorescence polarization data. X-ray diffraction data were recorded at beamline ID-24-E (operated by the Northeast Collaborative Access team: NE-CAT) at the Advanced Photon Source (APS, Argonne National Laboratory). We thank the staff of the NE-CAT beamlines for help in data collection. NE-CAT is supported by NIH grant P30 GM124165, using resources of the APS, operated by Argonne National Laboratory under Contract DE-AC02-06CH11357. JC and HZ are supported by RO1 GM116897. SCH is an Investigator in the Howard Hughes Medical Institute.

Author contributions

Sunbin Deng: Data curation; formal analysis; validation; investigation; visualization; methodology; writing – review and editing. **Jiaxi Cai:** Data curation; formal analysis; investigation; methodology; writing – review and editing. **Stephen C Harrison:** Formal analysis; writing – original draft; writing – review and editing. **Huilin Zhou:** Data curation; formal analysis; investigation; methodology; writing – review and editing. **Stephen M Hinshaw:** Visualization; methodology; writing – original draft; writing – review and editing.

Disclosure and competing interests statement

The authors declare that they have no conflict of interest.

References

Adams PD, Afonine PV, Bunkoczi G, Chen VB, Davis IW, Echols N, Headd JJ, Hung LW, Kapral GJ, Grosse-Kunstleve RW *et al* (2010) PHENIX: a comprehensive Python-based system for macromolecular structure solution. *Acta Crystallogr D Biol Crystallogr* 66: 213–221

de Albuquerque CP, Liang J, Gaut NJ, Zhou H (2016) Molecular circuitry of the SUMO (small ubiquitin-like modifier) pathway in controlling sumoylation homeostasis and suppressing genome rearrangements. *J Biol Chem* 291: 8825–8835

Anedchenko EA, Samel-Pommerencke A, Tran Nguyen TM, Shahnejat-Bushehri S, Popsel J, Lauster D, Herrmann A, Rappsilber J, Cuomo A, Bonaldi T *et al* (2019) The kinetochore module Okp1(CENP-Q)/Ame1(CENP-U) is a reader for N-terminal modifications on the centromeric histone Cse4(CENP-A). *EMBO J* 38: e98991

Boeckmann L, Takahashi Y, Au WC, Mishra PK, Choy JS, Dawson AR, Szeto MY, Waybright TJ, Heger C, McAndrew C *et al* (2013) Phosphorylation of centromeric histone H3 variant regulates chromosome segregation in *Saccharomyces cerevisiae*. *Mol Biol Cell* 24: 2034–2044

Cheeseman IM, Anderson S, Jwa M, Green EM, Kang J, Yates JR 3rd, Chan CS, Drubin DG, Barnes G (2002) Phospho-regulation of kinetochore-microtubule attachments by the Aurora kinase Ipl1p. *Cell* 111: 163–172

Chen Y, Baker RE, Keith KC, Harris K, Stoler S, Fitzgerald-Hayes M (2000) The N terminus of the centromere H3-like protein Cse4p performs an essential function distinct from that of the histone fold domain. *Mol Cell Biol* 20: 7037–7048

De Wulf P, McAinsh AD, Sorger PK (2003) Hierarchical assembly of the budding yeast kinetochore from multiple subcomplexes. *Genes Dev* 17: 2902–2921

Dendooven T, Zhang Z, Yang J, McLaughlin SH, Schwab J, Scheres SHW, Yatskevich S, Barford D (2023) Cryo-EM structure of the complete inner kinetochore of the budding yeast point centromere. *Sci Adv* 9: eadg7480

Dimitrova YN, Jenni S, Valverde R, Khin Y, Harrison SC (2016) Structure of the MIND complex defines a regulatory focus for yeast kinetochore assembly. *Cell* 167: 1014–1027

Emsley P, Lohkamp B, Scott WG, Cowtan K (2010) Features and development of Coot. *Acta Crystallogr D Biol Crystallogr* 66: 486–501

Evans PR, Murshudov GN (2013) How good are my data and what is the resolution? *Acta Crystallogr D Biol Crystallogr* 69: 1204–1214

Fischbock-Halwachs J, Singh S, Potocnjak M, Hagemann G, Solis-Mezarino V, Woike S, Ghodgaonkar-Steger M, Weissmann F, Gallego LD, Rojas J *et al* (2019) The COMA complex interacts with Cse4 and positions Sli15/Ipl1 at the budding yeast inner kinetochore. *Elife* 8: e42879

Guan R, Lian T, Zhou BR, He E, Wu C, Singleton M, Bai Y (2021) Structural and dynamic mechanisms of CBF3-guided centromeric nucleosome formation. *Nat Commun* 12: 1763

Hamilton GE, Helgeson LA, Noland CL, Asbury CL, Dimitrova YN, Davis TN (2020) Reconstitution reveals two paths of force transmission through the kinetochore. *Elife* 9: e56582

Hewawasam G, Shivaraju M, Mattingly M, Venkatesh S, Martin-Brown S, Florens L, Workman JL, Gerton JL (2010) Psh1 is an E3 ubiquitin ligase that targets the centromeric histone variant Cse4. *Mol Cell* 40: 444–454

Hinshaw SM, Harrison SC (2019) The structure of the Ctf19c/CCAN from budding yeast. *Elife* 8: e44239

Hinshaw SM, Harrison SC (2020) The structural basis for kinetochore stabilization by Cnn1/CENP-T. *Curr Biol* 30: e3423

Hinshaw SM, Quan Y, Cai J, Zhou AL, Zhou H (2023) Multi-site phosphorylation of yeast Mif2/CENP-C promotes inner kinetochore assembly. *Curr Biol* 33: 688–696

Hoffmann G, Samel-Pommerencke A, Weber J, Cuomo A, Bonaldi T, Ehrenhofer-Murray AE (2018) A role for CENP-A/Cse4 phosphorylation on serine 33 in deposition at the centromere. *FEMS Yeast Res* 18 https://doi.org/10.1093/femsyr/fox094

Hornung P, Troc P, Malvezzi F, Maier M, Demianova Z, Zimniak T, Litos G, Lampert F, Schleiffer A, Brunner M *et al* (2014) A cooperative mechanism drives budding yeast kinetochore assembly downstream of CENP-A. *J Cell Biol* 206: 509–524

Ichikawa Y, Saitoh N, Kaufman PD (2018) An asymmetric centromeric nucleosome. *Elife* 7: e37911

Jumper J, Evans R, Pritzel A, Green T, Figurnov M, Ronneberger O, Tunyasuvunakool K, Bates R, Zidek A, Potapenko A *et al* (2021) Highly accurate protein structure prediction with AlphaFold. *Nature* 596: 583–589

Lichten M (2014) Tetrad, random spore, and molecular analysis of meiotic segregation and recombination. *Methods Mol Biol* 1205: 13–28

Migl D, Kschonsak M, Arthur CP, Khin Y, Harrison SC, Ciferri C, Dimitrova YN (2020) Cryoelectron microscopy structure of a yeast centromeric nucleosome at 2.7 a resolution. *Structure* 28: 363–370

Mishra PK, Wood H, Stanton J, Au WC, Eisenstatt JR, Boeckmann L, Sclafani RA, Weinreich M, Bloom KS, Thorpe PH *et al* (2021) Cdc7-mediated phosphorylation of Cse4 regulates high-fidelity chromosome segregation in budding yeast. *Mol Biol Cell* 32: ar15

Ohkuni K, Levy-Myers R, Warren J, Au WC, Takahashi Y, Baker RE, Basrai MA (2018) N-terminal sumoylation of centromeric histone H3 variant Cse4 regulates its proteolysis to prevent mislocalization to non-centromeric chromatin. *G3 (Bethesda)* 8: 1215–1223

Ortiz J, Stemmann O, Rank S, Lechner J (1999) A putative protein complex consisting of Ctf19, Mcm21, and Okp1 represents a missing link in the budding yeast kinetochore. *Genes Dev* 13: 1140–1155

© 2023 The Authors

Pot I, Knockleby J, Aneliunas V, Nguyen T, Ah-Kye S, Liszt G, Snyder M, Hieter P, Vogel J (2005) Spindle checkpoint maintenance requires Ame1 and Okp1. *Cell Cycle* 4: 1448 – 1456

Ranjitkar P, Press MO, Yi X, Baker R, MacCoss MJ, Biggins S (2010) An E3 ubiquitin ligase prevents ectopic localization of the centromeric histone H3 variant via the centromere targeting domain. *Mol Cell* 40: 455 – 464

Samel A, Cuomo A, Bonaldi T, Ehrenhofer-Murray AE (2012) Methylation of CenH3 arginine 37 regulates kinetochore integrity and chromosome segregation. *Proc Natl Acad Sci U S A* 109: 9029 – 9034

Schmitzberger F, Richter MM, Gordiyenko Y, Robinson CV, Dadlez M, Westermann S (2017) Molecular basis for inner kinetochore configuration through RWD domain-peptide interactions. *EMBO J* 36: 3458 – 3482

Winter G, Beilsten-Edmands J, Devenish N, Gerstel M, Gildea RJ, McDonagh D, Pascal E, Waterman DG, Williams BH, Evans G (2022) DIALS as a toolkit. *Protein Sci* 31: 232 – 250

Wisniewski J, Hajj B, Chen J, Mizuguchi G, Xiao H, Wei D, Dahan M, Wu C (2014) Imaging the fate of histone Cse4 reveals de novo replacement in S phase and subsequent stable residence at centromeres. *Elife* 3: e02203

Yan K, Zhang Z, Yang J, McLaughlin SH, Barford D (2018) Architecture of the CBF3-centromere complex of the budding yeast kinetochore. *Nat Struct Mol Biol* 25: 1103 – 1110

Yan K, Yang J, Zhang Z, McLaughlin SH, Chang L, Fasci D, Ehrenhofer-Murray AE, Heck AJR, Barford D (2019) Structure of the inner kinetochore CCAN complex assembled onto a centromeric nucleosome. *Nature* 574: 278 – 282

License: This is an open access article under the terms of the Creative Commons Attribution License, which permits use, distribution and reproduction in any medium, provided the original work is properly cited.

Expanded View Figures

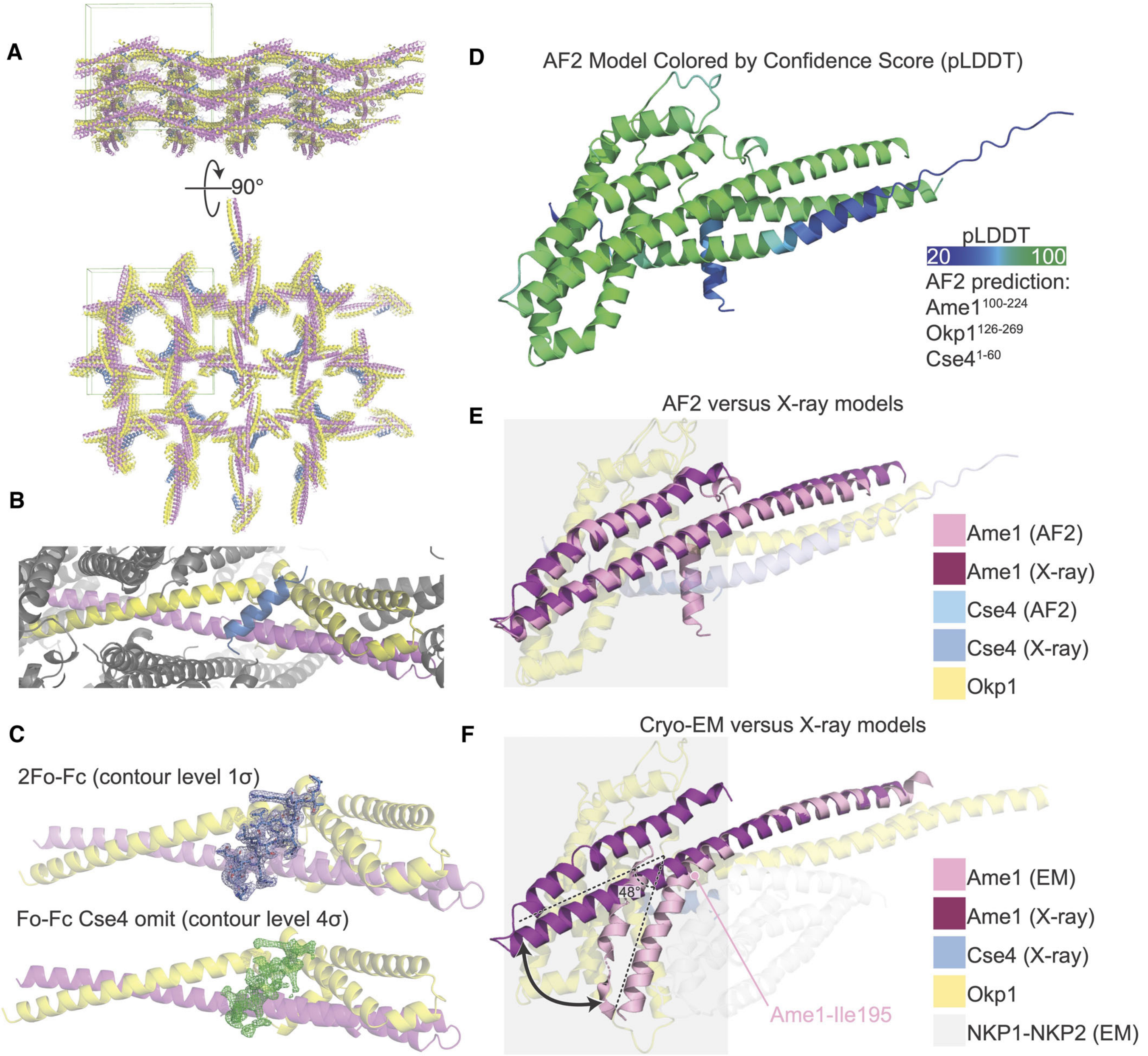

Figure EV1. Crystal structure of Okp1-Ame1-Cse4^{END} and flexibility at the Okp1-Ame1 head-coiled-coil joint and Nkp1-Nkp2 position.

A The crystal structure of Okp1-Ame1-Cse4 colored as in Fig 1. The green box shows the limits of a single unit cell.

B Close-up view of an individual biological protomer. Okp1-Ame1-Cse4 colored as in Fig 1. Neighboring protomers are colored gray.

C Cse4^{END} peptide density from the final refined model (top; 2Fo-Fc) and from the refined model lacking Cse4^{END} (bottom; Fo-Fc, Cse4 omitted).

D The structure of Okp1-Ame1-Cse4 as predicted by AlphaFold 2 (AF2) (Jumper *et al*, 2021). The model is colored according to confidence score (pLDDT) from low (blue) to high (green). The peptides used for prediction are given at right.

E Overlay of Okp1-Ame1-Cse4 from AF2 with the current Okp1-Ame1-Cse4 crystal structure (X-ray). Only Ame1 (magenta and pink) is shown as an opaque chain for clarity. The gray box marks the Okp1-Ame1 head domain.

F Overlay of the Okp1-Ame1-Cse4 structure from cryo-EM (EM) with the current crystal structure. The angle between the head and coiled coil shaft is indicated for the cryo-EM structure. Ame1-Leu195, which is the position at which Ame1 bends in the cryo-EM structure, is annotated. The Okp1-Ame1 head domain is marked as in panel E. Structures were aligned on the Okp1-Ame1 coiled coil shaft. The Nkp1-Nkp2 structure from cryo-EM (NKP1$^{2-76}$; NKP2$^{4-84}$) is shown as transparent gray chains.

 © 2023 The Authors

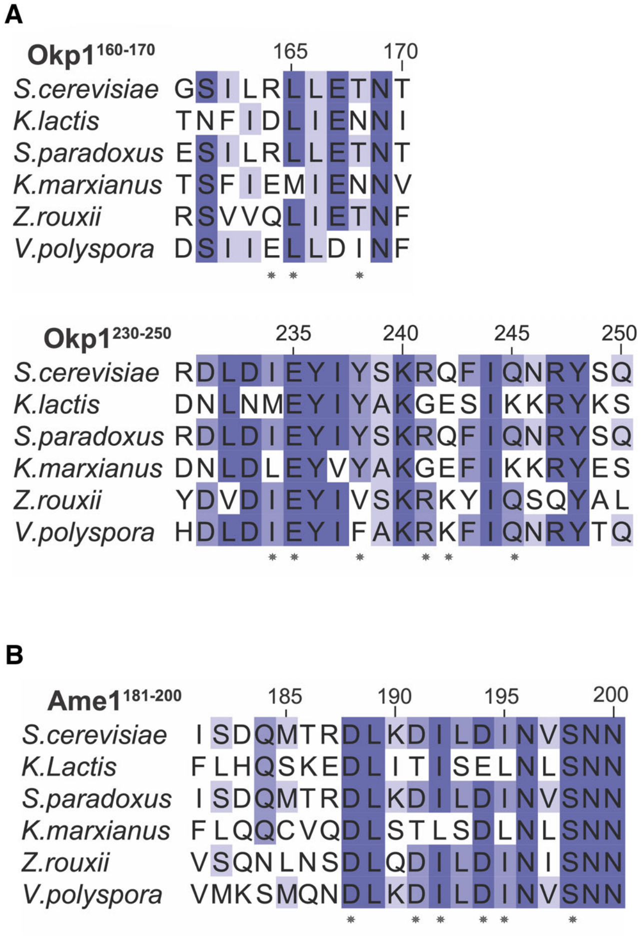

Figure EV2. Protein sequence alignments for Okp1 and Ame1 covering the Cse4^{END} contacts shown in Fig 2.

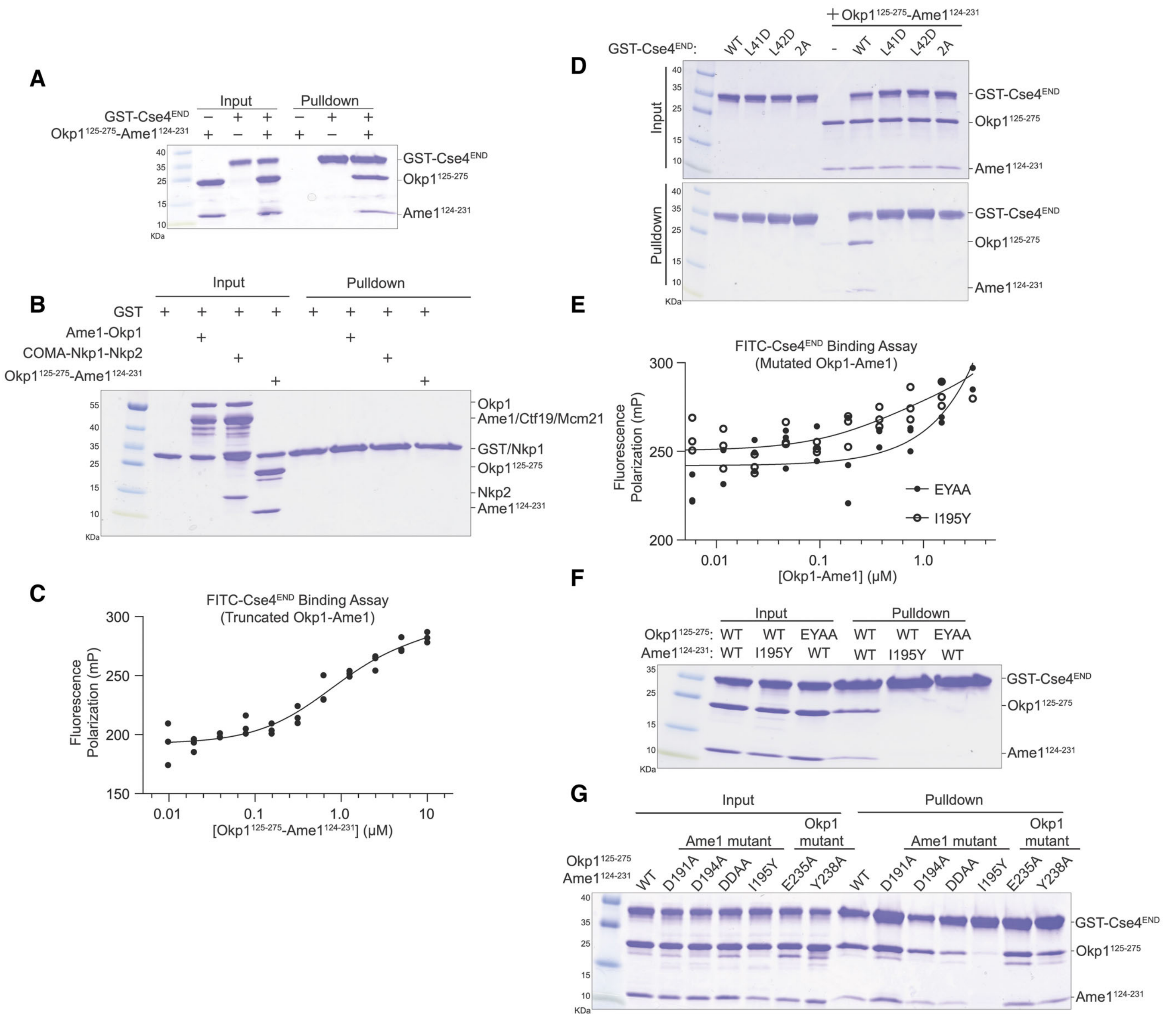

Figure EV3. Further biochemical characterization of the Okp1-Ame1-Cse4^{END} interaction.

A Pulldown assay showing binding between the truncated Okp1-Ame1 complex used for crystallography (Okp1$^{125-275l}$-Ame1$^{124-231}$) and GST-Cse4^{END}.

B Recombinant proteins used for pulldowns in Fig 3 were tested for their association with GST to determine the level of non-specific binding. Results of a GST pulldown assay are shown.

C The minimal Okp1-Ame1 complex used for crystallography was tested for its association with FITC-Cse4^{END} in a fluorescence polarization experiment. The measured dissociation constant is ~750 nM (see Materials and Methods; n = 3 independent experiments).

D GST-Cse4^{END} and its mutants were tested for Okp1-Ame1 binding.

E Full-length mutant Okp1-Ame1 complex (EYAA or I195Y as indicated) was tested for its association with FITC-Cse4^{END}.

F Various Okp1-Ame1 mutants (indicated above) were tested for binding to GST-Cse4^{END}.

G Various Okp1-Ame1 mutants were tested for Cse4^{END} binding as in panel F.

Source data are available online for this figure.

 © 2023 The Authors

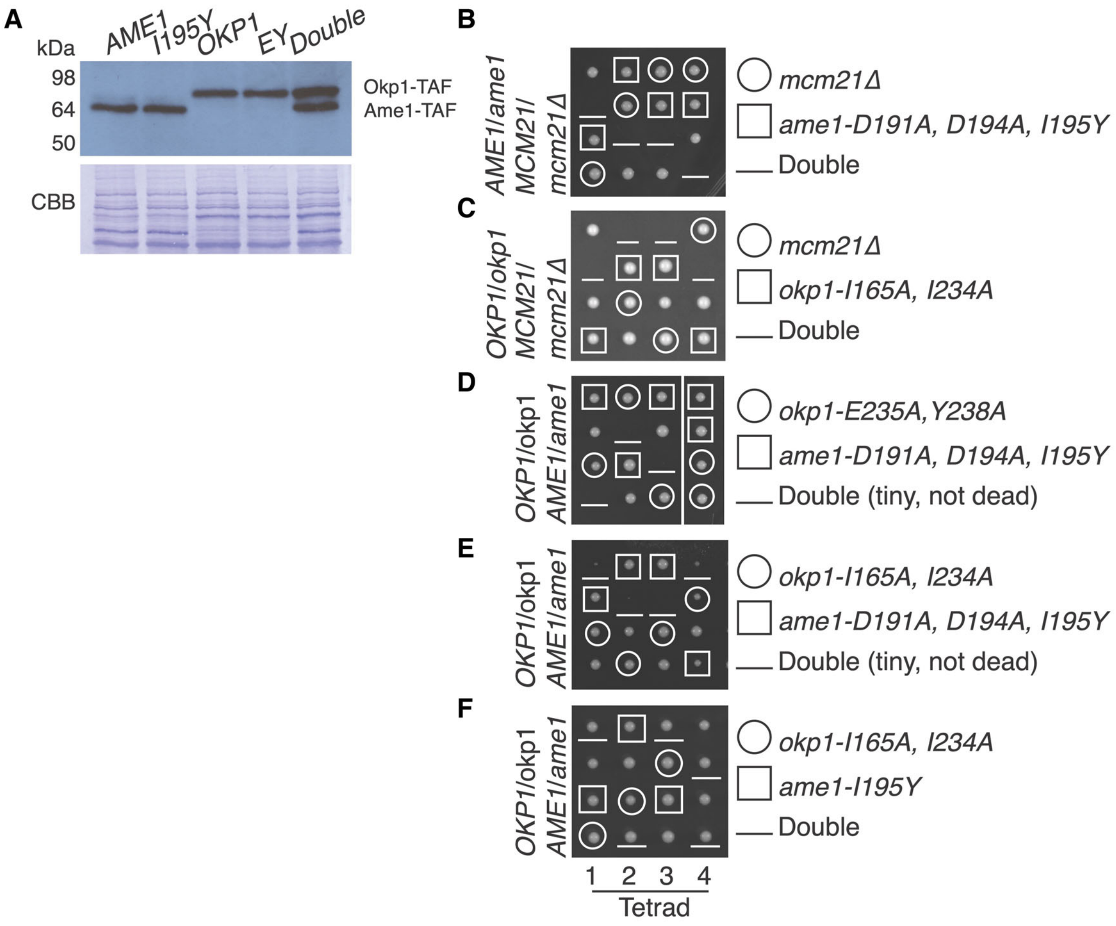

Figure EV4. ***In vivo* consequences of Okp1-Ame1 mutations.**

A Western blot showing expression of Ame1, Okp1, and their mutants in whole cell extracts (TAF – protein A-FLAG tag; anti-Protein A used for detection).

B–F Tetrad dissection results as in Fig 4B–D. The mutants tested and the resulting spore genotypes are shown at right.

Source data are available online for this figure.

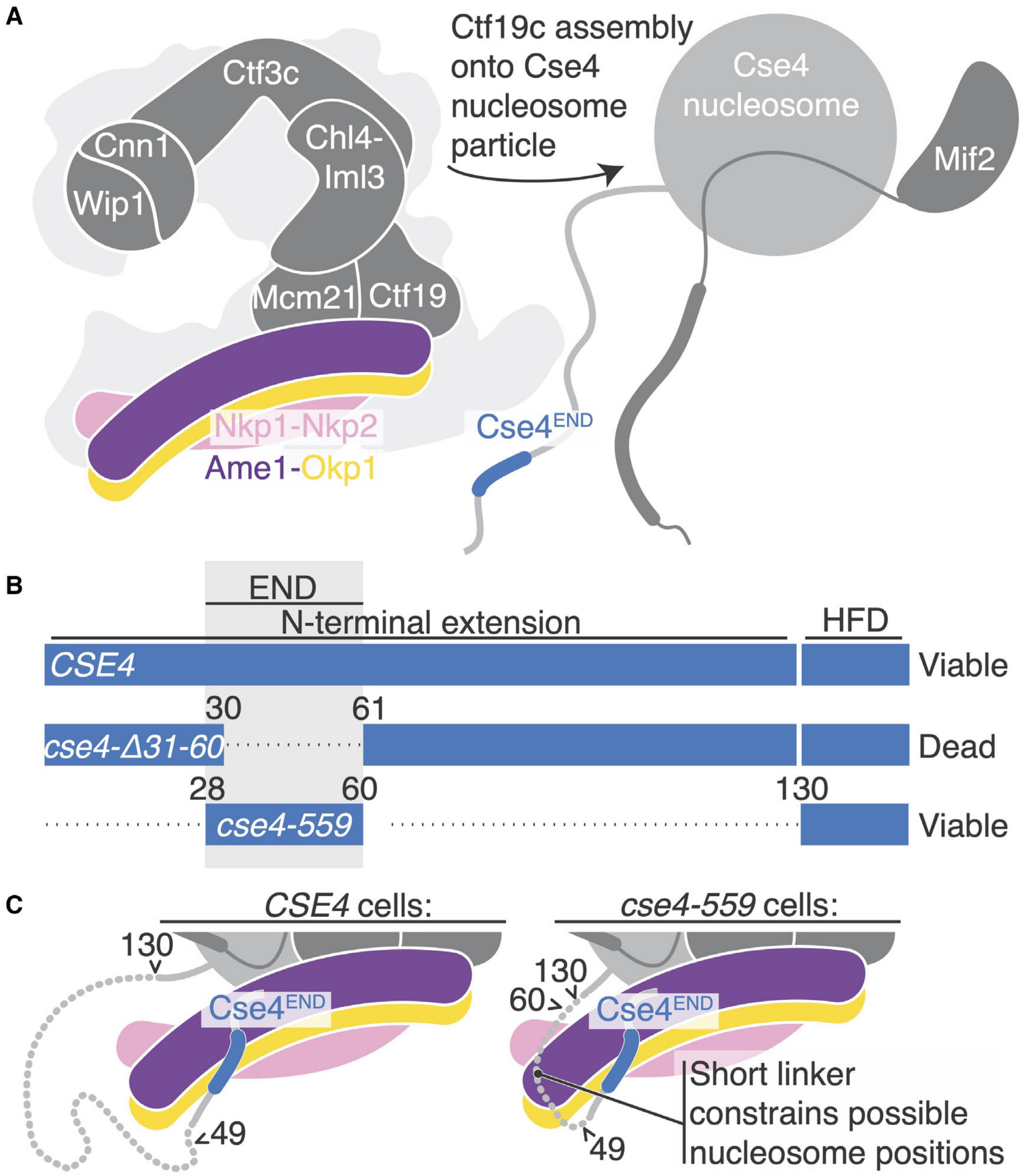

Figure EV5. Model for Cse4 nucleosome contact by the Ctf19c and relation to *cse4* alleles.

A Schematic showing Ctf19c assembly onto the Cse4-Mif2 complex. The assembly occurs via a largely unknown biochemical mechanism.

B *cse4* alleles (white text), their corresponding Cse4 proteins (blue bars), and their reported abilities to support cell viability (right). The alleles were reported by Chen *et al* (2000) and Fischbock-Halwachs *et al* (2019). The dotted lines indicate omitted fragments. The numbers correspond to full length Cse4. A gray box marks the boundaries of Cse4^{END}. HFD – histone fold domain.

C Structural view of the Cse4 N-terminal extension in *CSE4* (left) or *cse4-559* (right) cells. Arrows and nearby numbers point to Cse4 amino acid positions according to their numbering in the full-length protein.

 © 2023 The Authors
