# Supplementary material for: Recognition of centromere‐specific histone Cse4 by the inner kinetochore Okp1‐Ame1 complex
Source: EMBO Rep. 2023 Nov 20;24(12):e57702. doi: 10.15252/embr.202357702 (PMC10702835; doi:10.15252/embr.202357702)
Supplement: Supplementary file 3 — Table EV1 [file EMBR-24-e57702-s005.docx]

**Table EV1.** Crystallographic and model statistics for the Okp1-Ame1-Cse4 crystal structure.

| Statistics for Cse4^END^-Okp1^125-275^-Ame1^124-231^ crystal structure | |
| --- | --- |
| Crystal Parameters |  |
| Space group | P 42 21 2 (No. 94) |
| Unit cell dimension | 154.305 (90) |
|  | 154.305 (90) |
|  | 37.114 (90) |
| Data collection |  |
| Wavelength (Å) | 0.97918 |
| Resolution (Å) | 109.11 -1.73 (1.76-1.73) |
| Unique reflections | 47590 (2301) |
| R_merge_ | 0.125 (4.729) |
| R_meas_ | 0.128 (4.818) |
| R_pim_ | 0.025 (0.915) |
| CC_½_ | 1.000 (0.355) |
| I/σ | 16.9 (1.0) |
| Completeness | 99.99% (97.75%) |
| Redundancy | 14.2% (14.1%) |
| Refinement |  |
| R_work_/R_free_ | 0.2067/0.2364 |
| R_msd_ |  |
| Bonds (Å) | 0.017 |
| Angels (°) | 1.554 |
| Average B factors (Å^2^) |  |
| Protein | 47.39 |
| Ramachandran statistics (%) |  |
| Favored | 97.83% |
| Allowed | 100% |
| Outliers | 0 |
| MolProbity clash score | 5.0 |
