# Supplementary material for: Recognition of centromere‐specific histone Cse4 by the inner kinetochore Okp1‐Ame1 complex
Source: EMBO Rep. 2023 Nov 20;24(12):e57702. doi: 10.15252/embr.202357702 (PMC10702835; doi:10.15252/embr.202357702)
Supplement: Supplementary file 4 — Table EV2 [file EMBR-24-e57702-s003.docx]

**Table EV2.** Yeast strains used in this work.

| Yeast strains | |
| --- | --- |
| HZY988 | *mcm21∆:Hyg*, MAT alpha, W303 |
| HZY1077 | MAT a, W303 (*ade2-1, can1-100, his3-1115, leu2-3, 112trp-1, ura3-1, RAD5+*) |
| HZY1078 | MAT alpha, W303 (*ade2-1, can1-100, his3-1115, leu2-3, 112trp-1, ura3-1, RAD5+*) |
| HZY1079 | Diploid by crossing HZY1078 with HZY1077 |
| HZY2869 | *ame1-2DA (D191A, D194A)-TAF:G418*, MAT a, W303 |
| HZY2870 | *ame1-I195Y-TAF:G418*, MAT a, W303 |
| HZY2871 | *ame1-triple (D191A, D194A, I195Y)-TAF:G418*, MAT a, W303 |
| HZY2872 | *okp1-E235A-TAF:G418*, MAT a, W303 |
| HZY2873 | *okp1-Y238A-TAF:G418*, MAT a, W303 |
| HZY2874 | *okp1-E235A, Y238A-TAF:G418*, MAT a, W303 |
| HZY2875 | *okp1-I165A, I234A-TAF:G418*, MAT a, W303 |
| HZY2876 | *okp1-I165A-TAF:G418*, MAT a, W303 |
| HZY2877 | Diploid by crossing HZY988 (*mcm21∆:Hyg*) with HZY2869 (*ame1-2DA-TAF:G418*) |
| HZY2878 | Diploid by crossing HZY988 (*mcm21∆:Hyg*) with HZY2873 (*okp1-Y238A-TAF:G418*) |
| HZY2879 | Diploid by crossing HZY988 (*mcm21∆:Hyg*) with HZY2874 (*okp1-E235A, Y238A-TAF:G418*) |
| HZY2880 | Diploid by crossing HZY988 (*mcm21∆:Hyg*) with HZY2875 (*okp1-I165A, I234A-TAF:G418*) |
| HZY2881 | Diploid by crossing HZY988 (*mcm21∆:Hyg*) with HZY2876 (*okp1-I165A-TAF:G418*) |
| HZY2882 | Diploid by crossing HZY988 (*mcm21∆:Hyg*) with HZY2872 *(okp1-E235A-TAF:G418*) |
| HZY2911 | Diploid by crossing HZY988 (*mcm21∆:Hyg*) with HZY2870 (*ame1-I195Y-TAF:G418*) |
| HZY2912 | Diploid by crossing HZY988 (*mcm21∆:Hyg*) with HZY2871 (*ame1-triple-TAF:G418*) |
| HZY2943 | *ame1-triple (D191A, D194A, I195Y)-TAF:G418*, MAT a, derived from HZY2912 |
| HZY2945 | *ame1-triple-TAF:G418*, MAT alpha, derived from HZY2912 |
| HZY2970 | *cse4∆:NAT*, pRS316-G419-3xFlag-*CSE4*, MAT alpha, W303 |
| HZY2971 | *okp1-E235A, Y238A-TAF:G418*, MAT alpha, W303, derived from HZY2879 dissection |
| HZY2976 | *okp1-I165A, I234A-TAF:G418*, MAT alpha, W303, derived from HZY2880 dissection |
| HZY2977 | *okp1-E235A, Y238A-TAF:NAT*, MAT alpha, W303, derived from HZY2971 |
| HZY2978 | *okp1-I165A, I234A-TAF:NAT*, MAT alpha, W303, derived from HZY2876 |
| HZY2979 | Diploid by crossing HZY2943 (*ame1-triple-TAF:G418*) with HZY2977 (*okp1-E235A, Y238A-TAF:Nat*) |
| HZY2980 | Diploid by crossing HZY2943 (*ame1-triple-TAF:G418*) with HZY2978 (*okp1-I165A, I234A-TAF:Nat*) |
| HZY2981 | Diploid by crossing HZY2943 (*ame1-I195Y-TAF:G418*) with HZY2977 (*okp1-E235A, Y238A-TAF:Nat*) |
| HZY2982 | Diploid by crossing HZY2943 (*ame1-I195Y-TAF:G418*) with HZY2978 (*okp1-I165A, I234A-TAF:Nat*) |
| HZY2983 | *okp1-I165D-TAF:G418*, MAT a, W303 |
| HZY2984 | *okp1-I234D-TAF:G418*, MAT a, W303 |
| HZY2985 | *okp1-I165D,I234D-TAF:G418*, MAT a, W303 |
| HZY2986 | *okp1-E235A, Y238A-TAF:Nat*, MAT a, W303, derived from HZY2979 dissection |
| HZY2988 | *okp1-E235A, Y238A-TAF:Nat*, *ame1-triple-TAF:G418*, MAT alpha, W303, derived from HZY2979 dissection |
| HZY2989 | *okp1-I165A, I234A-TAF:Nat*, MAT a, W303, derived from HZY2980 dissection |
| HZY2990 | *okp1-I165A, I234A-TAF:Nat*, *ame1-triple-TAF:G418*, MAT a, W303, derived from HZY2980 dissection |
| HZY2999 | *ame1-I195Y-TAF:G418 okp1-E235A/Y238A-TAF:Nat*, MAT alpha, derived from HZY2981 |
| HZY3900 | *ame1∆:NAT*, pRS316-*AME1*, MAT a, W303 |
| HZY3901 | ame1∆:NAT, pRS316-*AME1*, MAT alpha, W303 |
| HZY3904 | okp1∆:NAT, pRS316-*OKP1*, MAT a, W303 |
| HZY3905 | okp1∆:NAT, pRS316-*OKP1*, MAT alpha, W303 |
