# Supplementary material for: Recognition of centromere‐specific histone Cse4 by the inner kinetochore Okp1‐Ame1 complex
Source: EMBO Rep. 2023 Nov 20;24(12):e57702. doi: 10.15252/embr.202357702 (PMC10702835; doi:10.15252/embr.202357702)
Supplement: Supplementary file 5 — Table EV3 [file EMBR-24-e57702-s002.docx]

**Table EV3.** Plasmids used in this work.

| Plasmids | |
| --- | --- |
| HZE2612 | pRS315-*OKP1* |
| HZE2613 | pRS316-*OKP1* |
| HZE2614 | pRS315-*AME1* |
| HZE2623 | pRS316-*AME1* |
| HZE2662 | pRS316-*AME1-TAF:HisMX* |
| HZE2663 | pRS316-*AME1-TAF:G418* |
| HZE2665 | pRS315-*CSE4* |
| HZE2666 | pRS316-*CSE4* |
| HZE2719 | pRS316-*G418-3xFlag-CSE4* |
| HZE3198 | pRS315-*OKP1-TAF:G418* |
| HZE3309 | pRS315-*ame1-D191A,D194A-TAF:G418* |
| HZE3310 | pRS315-*ame1-I195Y-TAF:G418* |
| HZE3311 | pRS315-*okp1-E235A-TAF:G418* |
| HZE3312 | pRS315-*okp1-Y238A-TAF:G418* |
| HZE3323 | pRS315-*ame1-D191A, D194A, I195Y-TAF:G418* |
| HZE3324 | pRS315-*okp1-E235A, Y238A-TAF:G418* |
| HZE3336 | pRS315-*okp1-L165A, I234A-TAF:G418* |
| HZE3337 | pRS315-*G418-3xFlag-cse4-L41A* |
| HZE3342 | pRS315-*okp1-L165A-TAF:G418* |
| HZE3344 | pRS315-*G418-3xFlag-cse4-L41D* |
| HZE3345 | pRS315-*G418-3xFlag-cse4-L42D* |
| HZE3346 | pRS315-*G418-3xFlag-cse4-L41A, L42A* |
| pFS1 | pET3aTra His-TEV-Mcm21;Ctf19(untagged) |
| pFS174 | pLIC-Tra His-TEV-Nkp1;Nkp2(untagged) (Schmitzberger et al., 2017) |
| pTDAO | pET28 Ame1-6His;Okp1(untagged) (Hinshaw and Harrison, 2019) |
| pSD2613 | pETDuet- His-TEV-Okp1^125-275^ - Ame1^124-231^ (crystal construct) |
| pSD2614 | pSD2613 (Ame1-D191A) |
| pSD2615 | pSD2613 (Ame1-D194A) |
| pSD2616 | pSD2613 (Ame1-D191A-D194A) |
| pSD2617 | pSD2613 (Ame1-I195Y) |
| pSD2618 | pSD2613 (Okp11-E235A) |
| pSD2619 | pSD2613 (Okp1-Y238A) |
| pSD2620 | pSD2613 (Okp1-E235A-Y238A) |
| pSD2621 | pTDAO (Ame1-I195Y) |
| pSD2622 | pTDAO (Okp1-E235A-Y238A) |
| pSD2623 | pLIC-Tra His-GST-TEV-Cse4^28-60^ |
| pSD2624 | pLIC-Tra His-GST-TEV-Cse4^28-60^(L41D) |
| pSD2625 | pLIC-Tra His-GST-TEV-Cse4^28-60^(L42D) |
| pSD2626 | pLIC-Tra His-GST-TEV-Cse4^28-60^(L41D) |
