# Supplementary figures and images for: Recognition of centromere‐specific histone Cse4 by the inner kinetochore Okp1‐Ame1 complex
Source: EMBO Rep. 2023 Nov 20;24(12):e57702. doi: 10.15252/embr.202357702 (PMC10702835; doi:10.15252/embr.202357702)

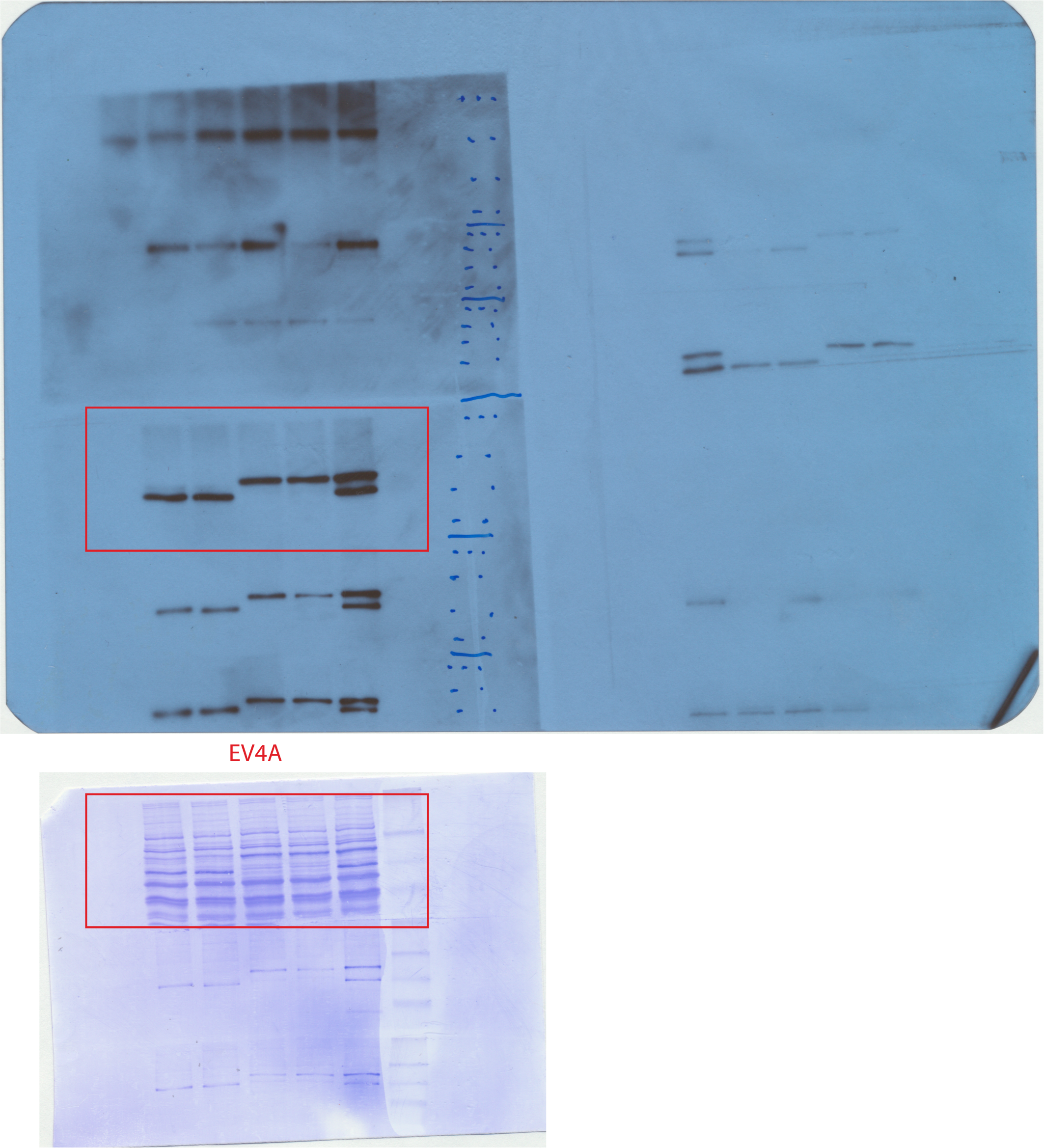

Supplement: Supplementary file 6 — Source Data for Expanded View [file EMBR-24-e57702-s010.zip › Figure_EV4/EV4A/EV4A.tif]

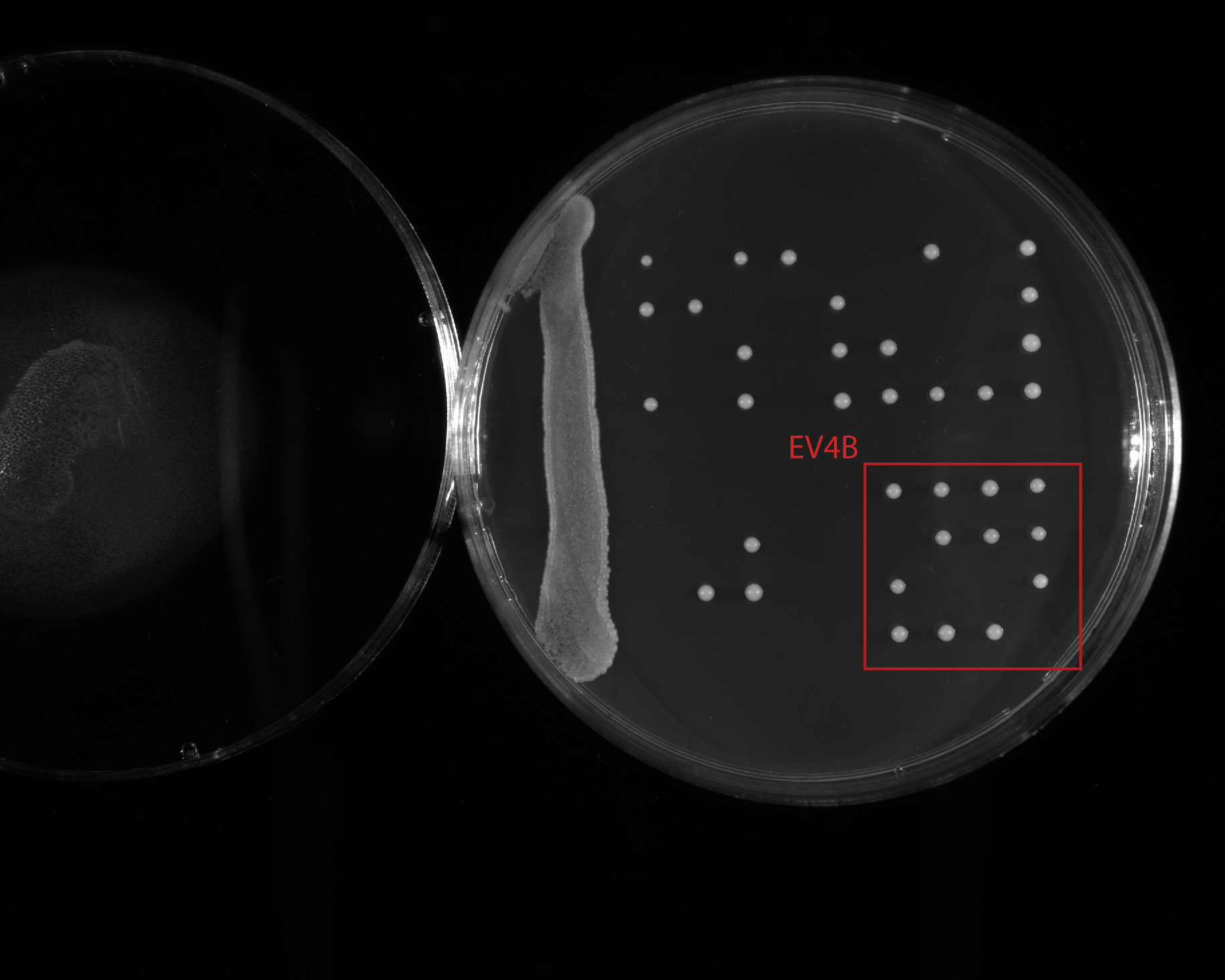

Supplement: Supplementary file 6 — Source Data for Expanded View [file EMBR-24-e57702-s010.zip › Figure_EV4/EV4B/EV4B.tif]

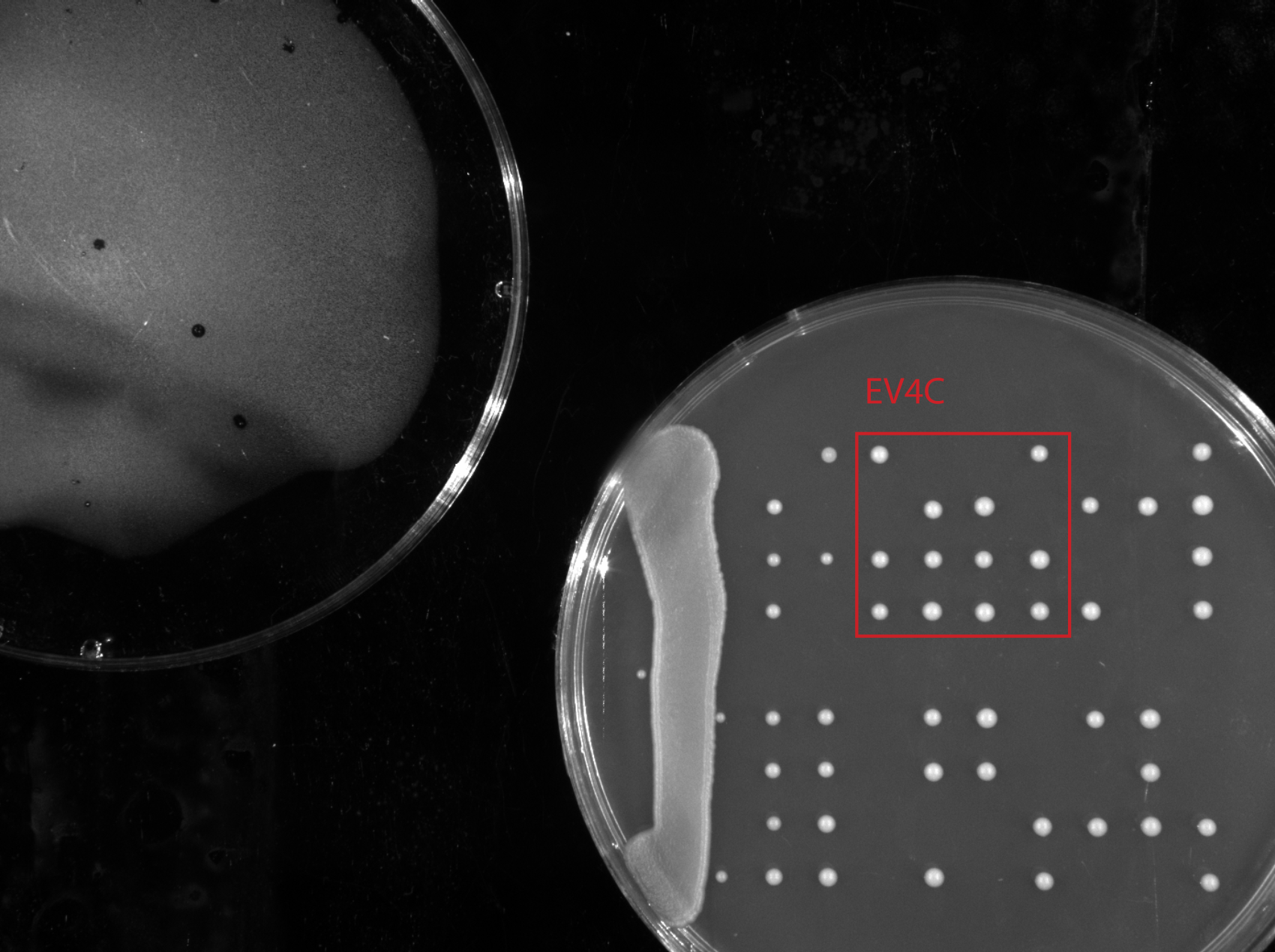

Supplement: Supplementary file 6 — Source Data for Expanded View [file EMBR-24-e57702-s010.zip › Figure_EV4/EV4C/EV4C.tif]

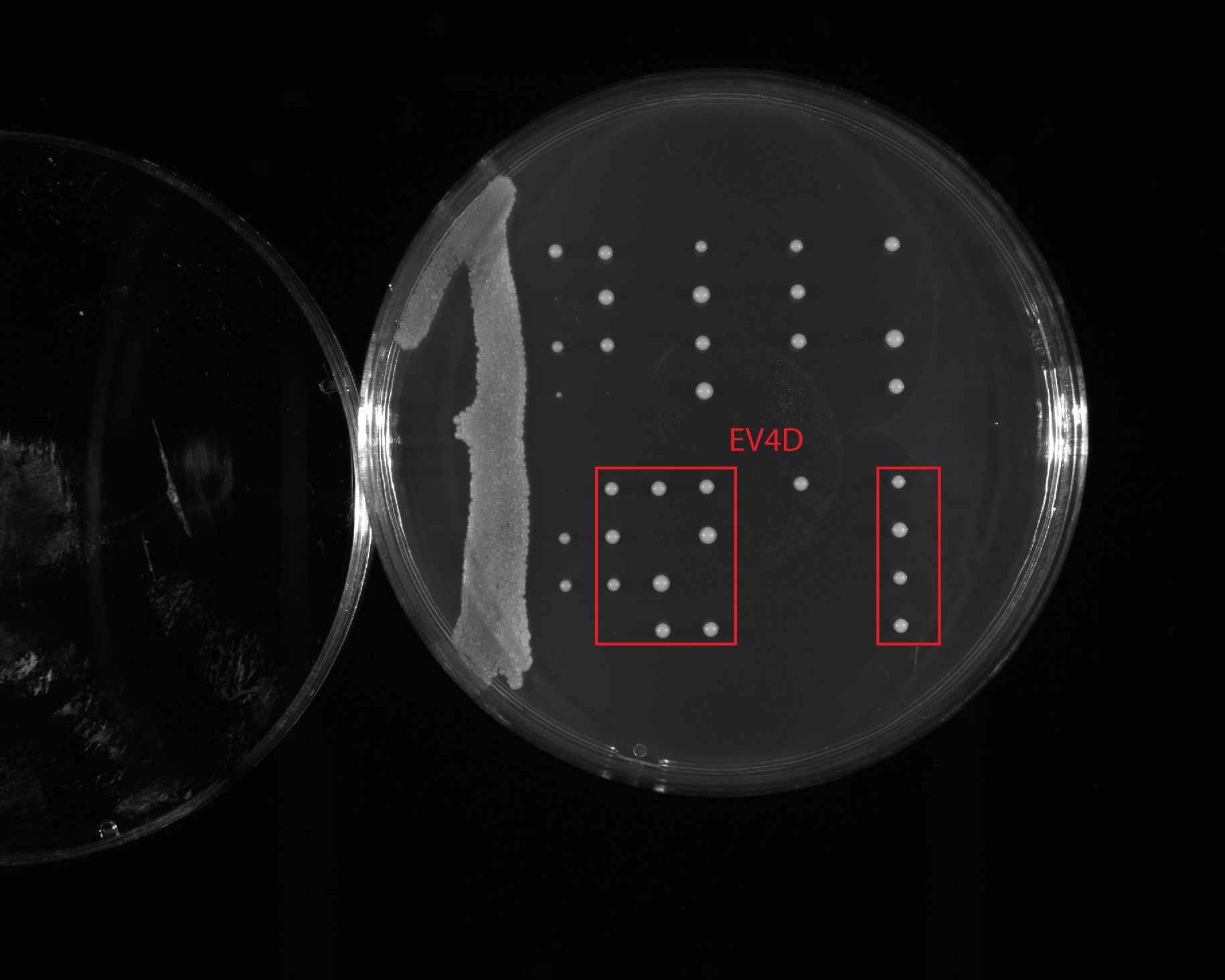

Supplement: Supplementary file 6 — Source Data for Expanded View [file EMBR-24-e57702-s010.zip › Figure_EV4/EV4D/EV4D.tif]

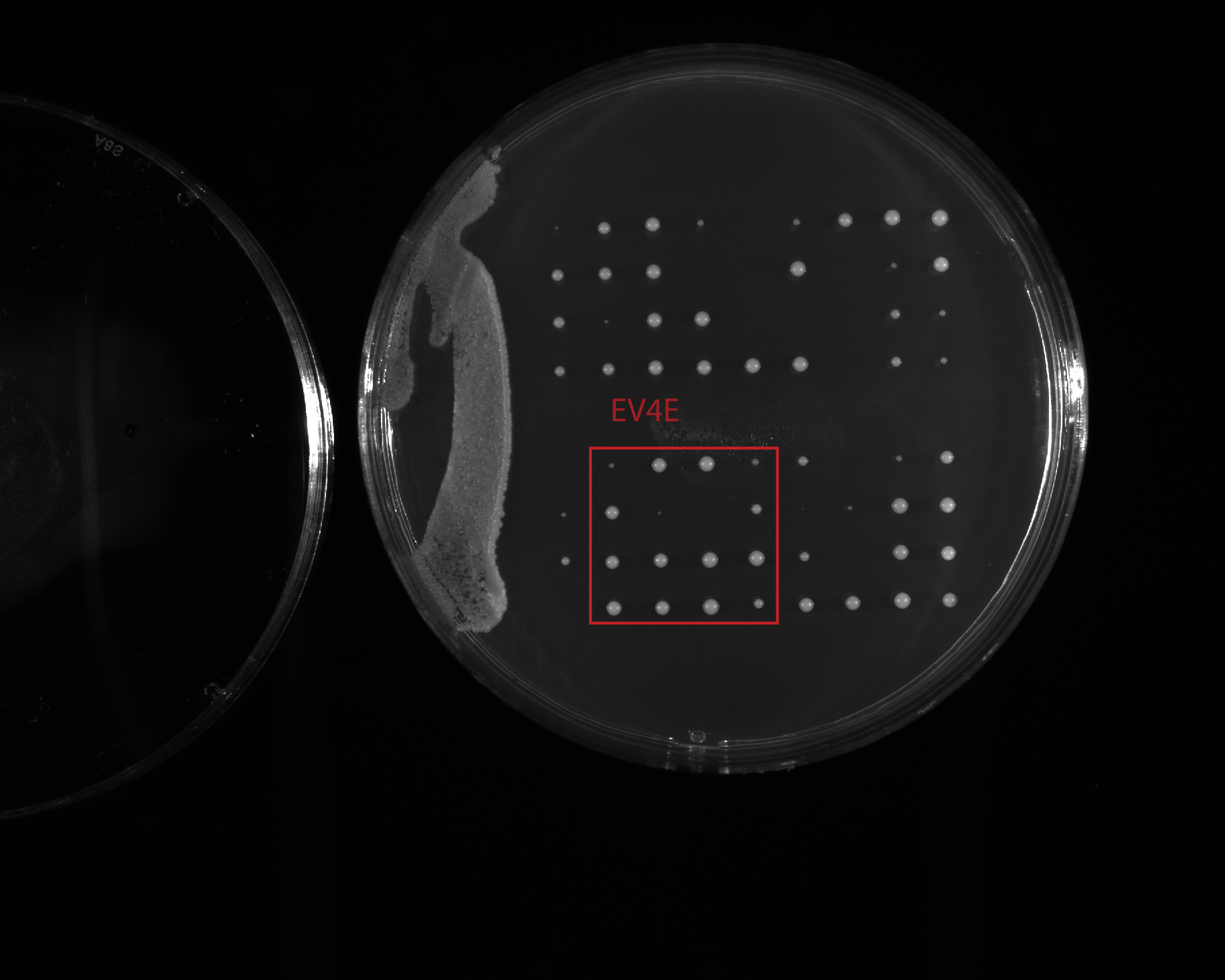

Supplement: Supplementary file 6 — Source Data for Expanded View [file EMBR-24-e57702-s010.zip › Figure_EV4/EV4E/EV4E.tif]

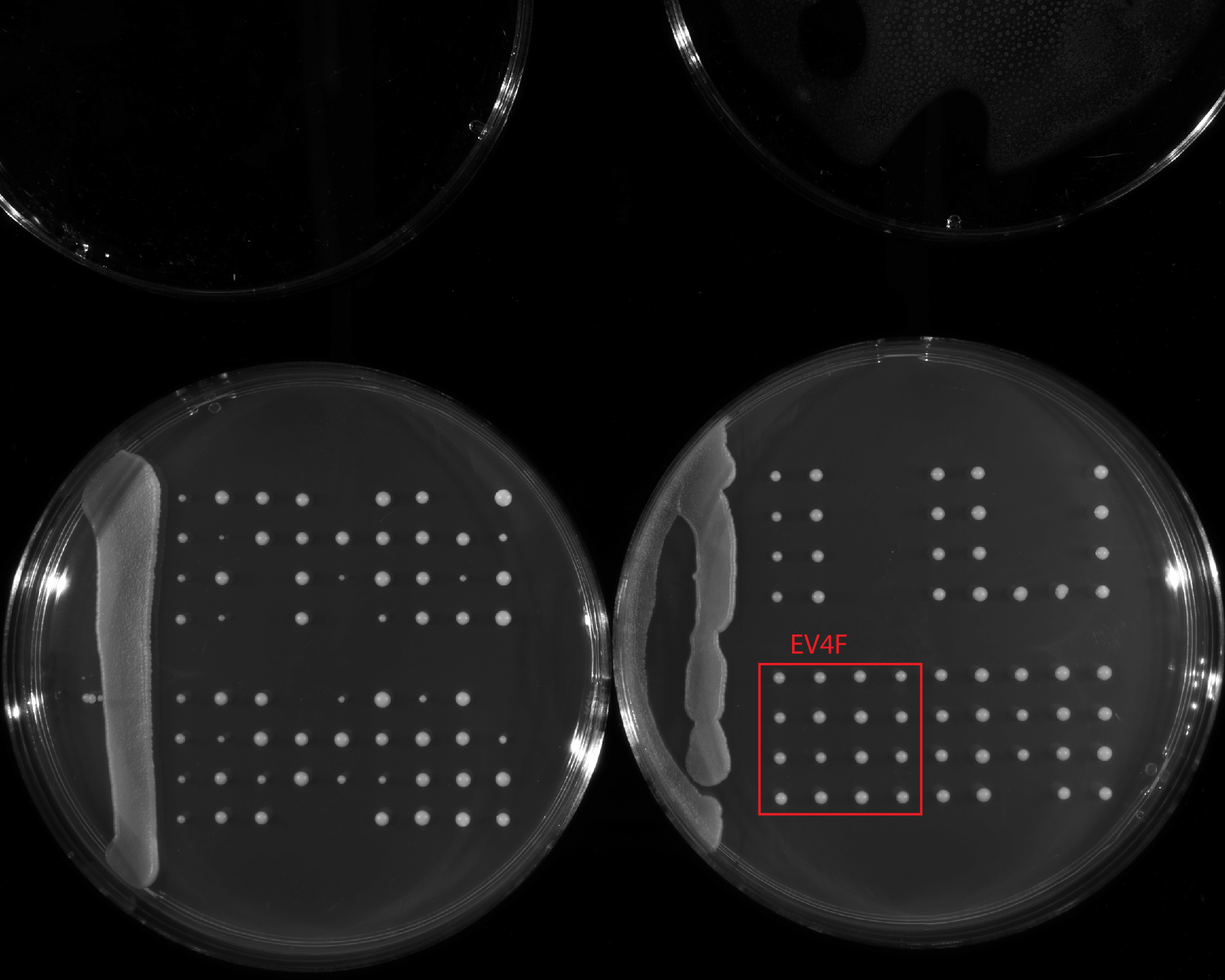

Supplement: Supplementary file 6 — Source Data for Expanded View [file EMBR-24-e57702-s010.zip › Figure_EV4/EV4F/EV4F.tif]

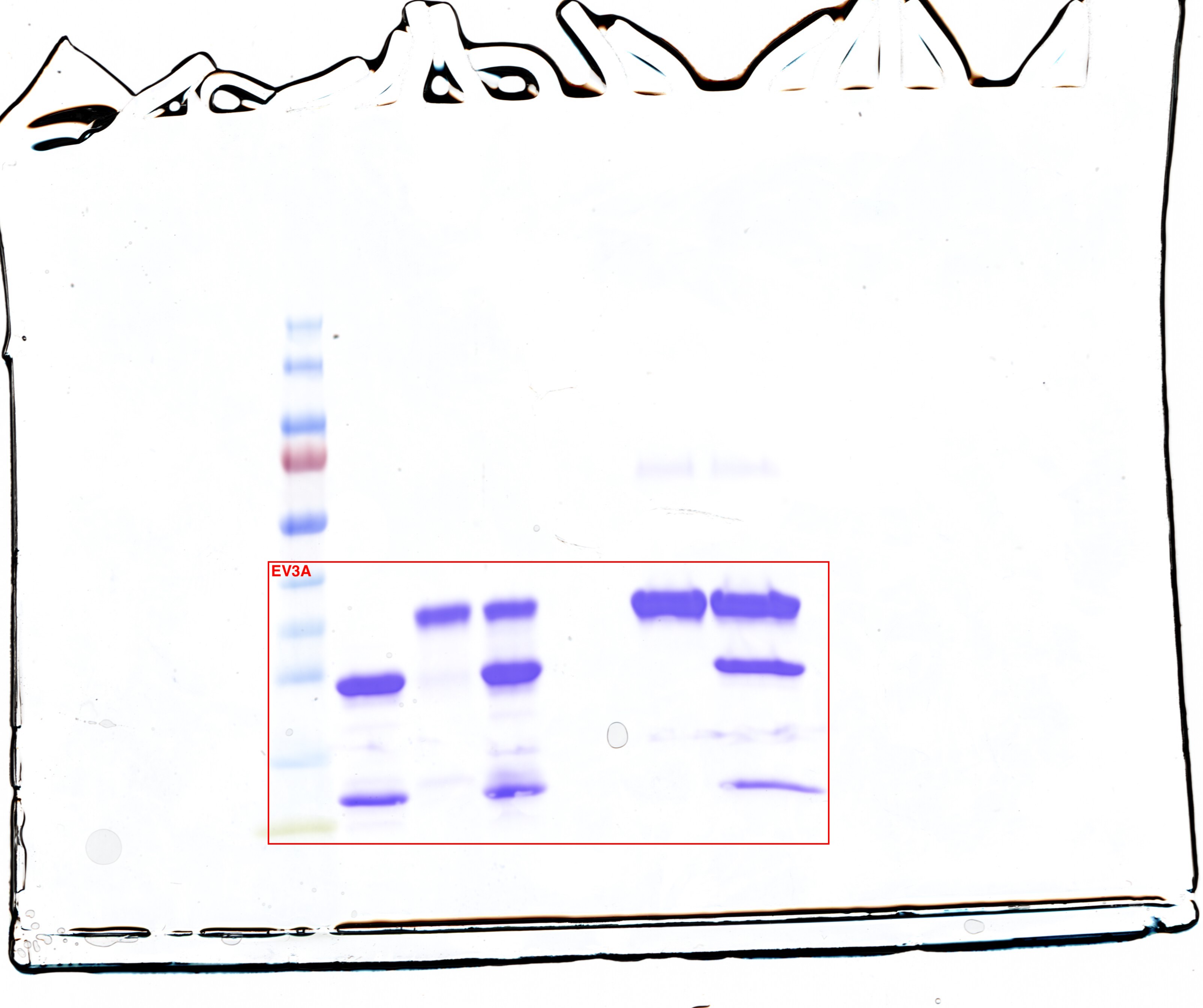

Supplement: Supplementary file 6 — Source Data for Expanded View [file EMBR-24-e57702-s010.zip › Figure_EV3/EV3A/Protein_gel_panel_A.jpg]

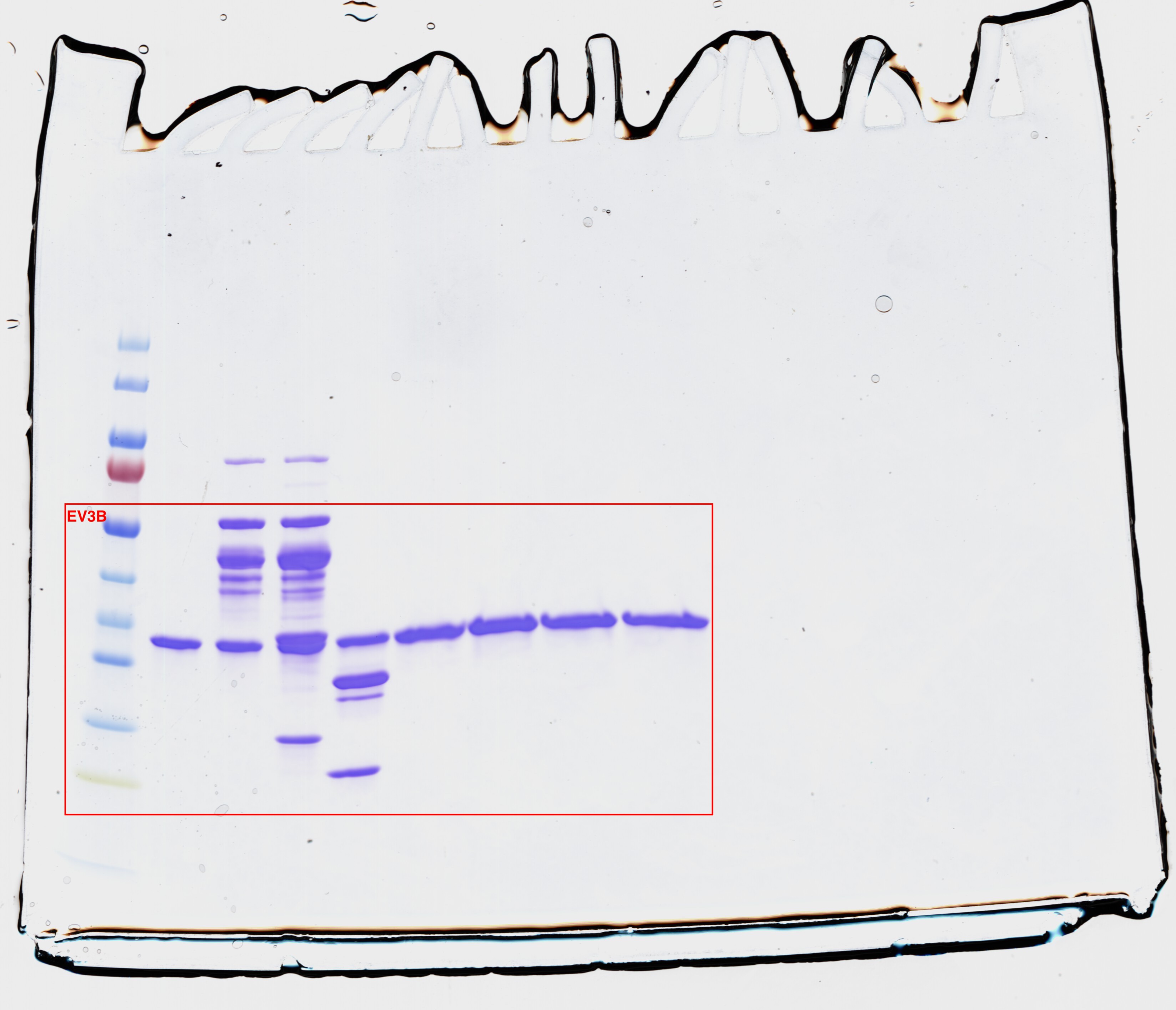

Supplement: Supplementary file 6 — Source Data for Expanded View [file EMBR-24-e57702-s010.zip › Figure_EV3/EV3B/Protein_gel_panel_EV3B.jpg]

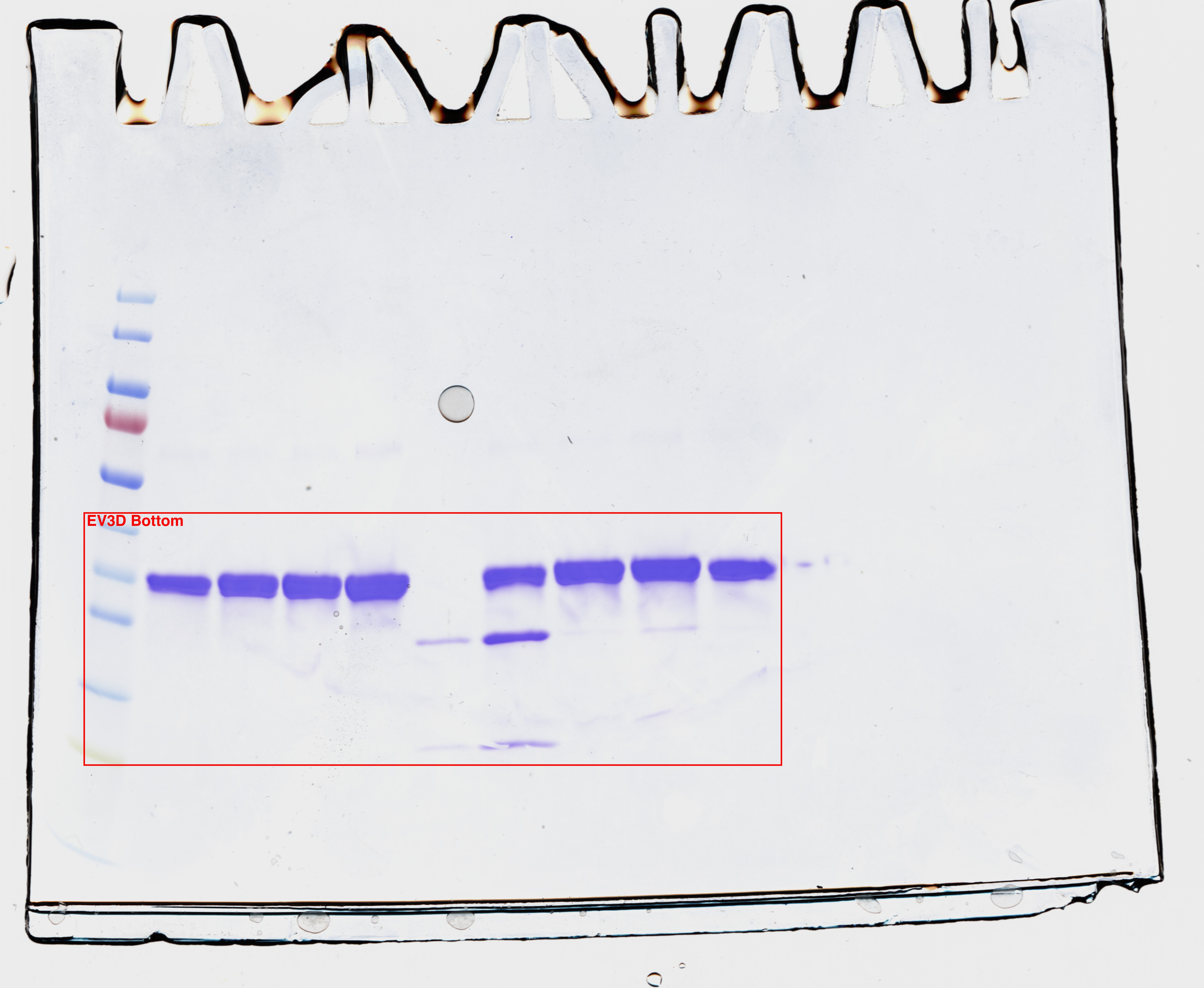

Supplement: Supplementary file 6 — Source Data for Expanded View [file EMBR-24-e57702-s010.zip › Figure_EV3/EV3D/Protein_gel_panel_D_bottom.jpg]

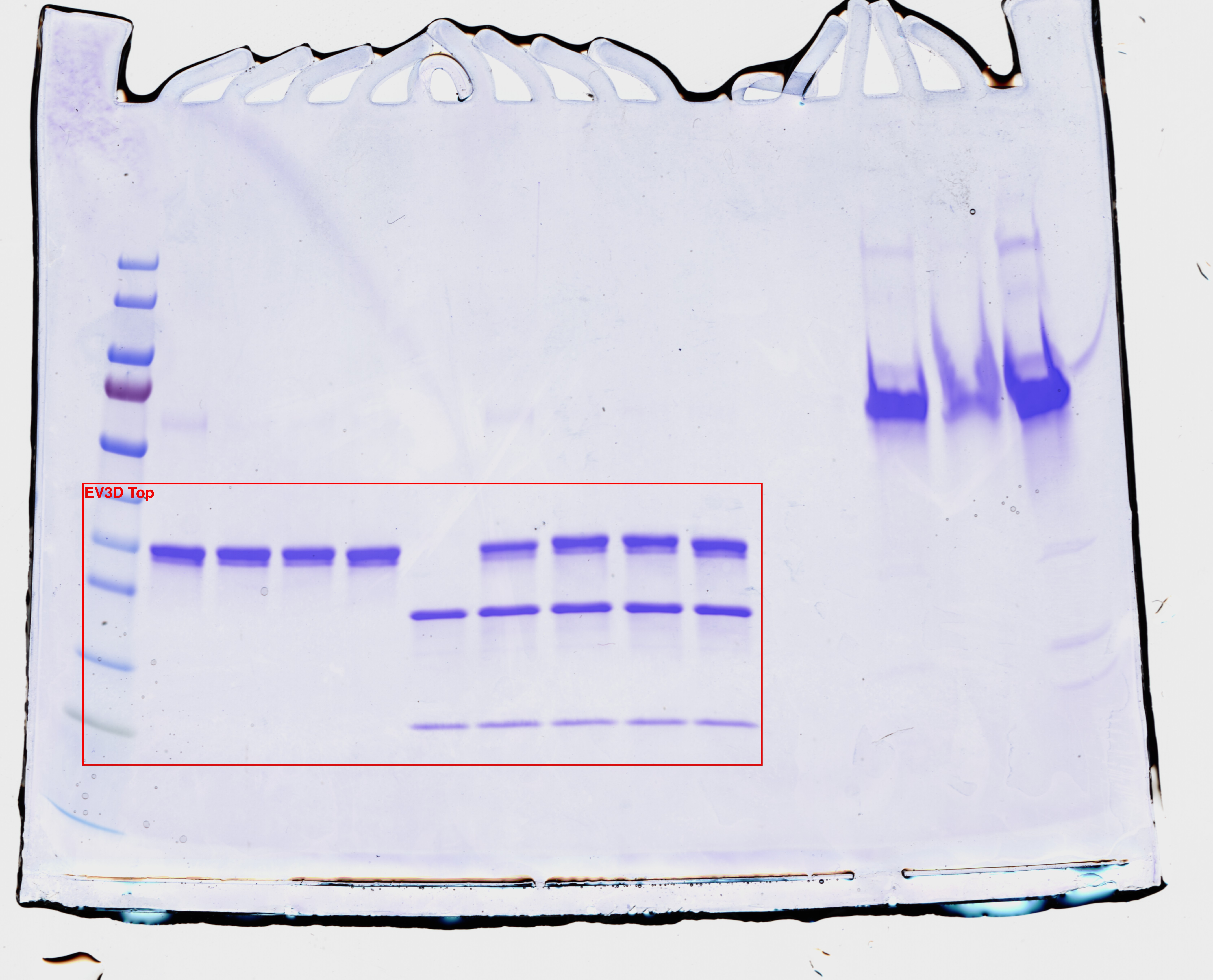

Supplement: Supplementary file 6 — Source Data for Expanded View [file EMBR-24-e57702-s010.zip › Figure_EV3/EV3D/Protein_gel_panel_D_top.jpg]

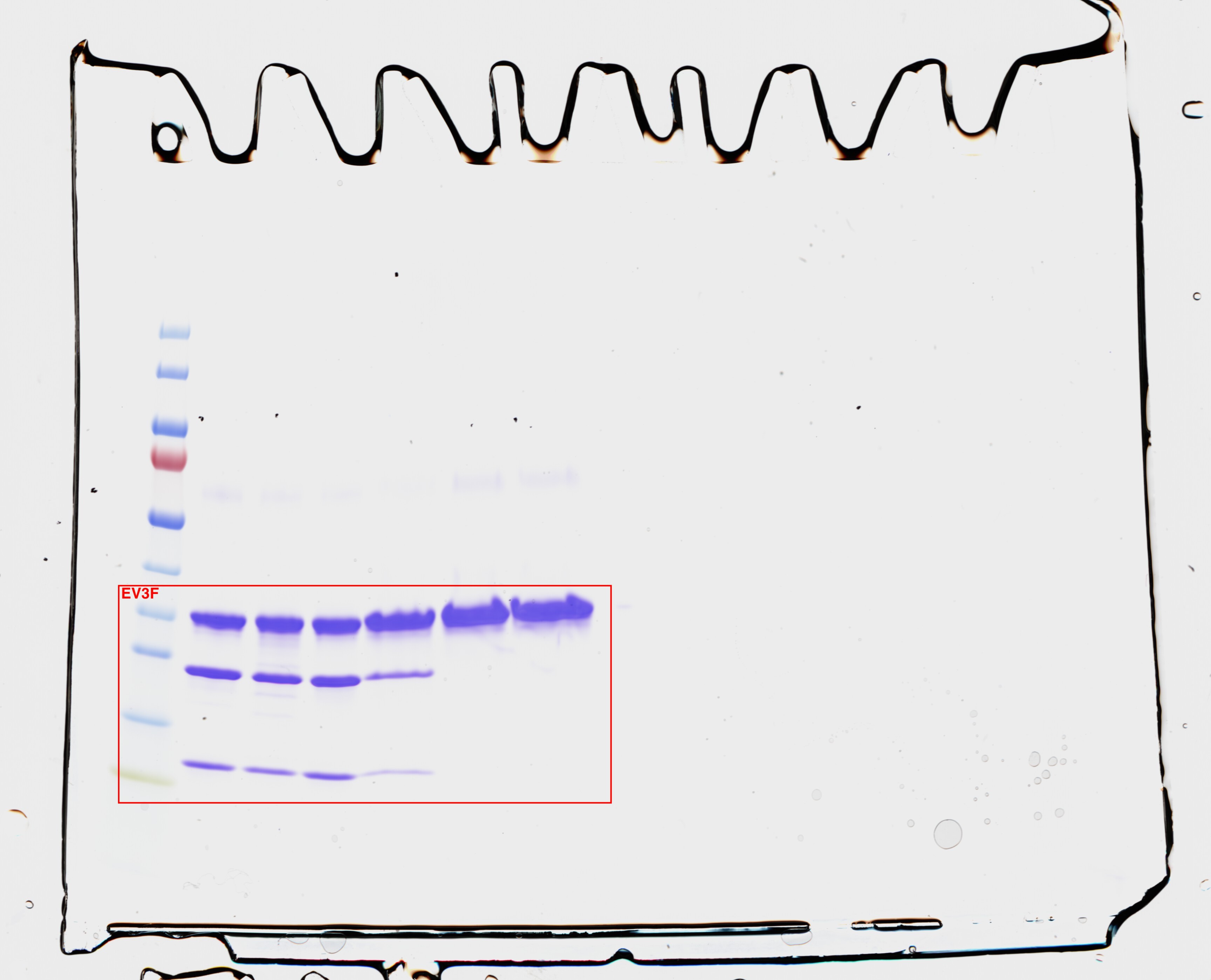

Supplement: Supplementary file 6 — Source Data for Expanded View [file EMBR-24-e57702-s010.zip › Figure_EV3/EV3F/Protein_gel_panel_F.jpg]

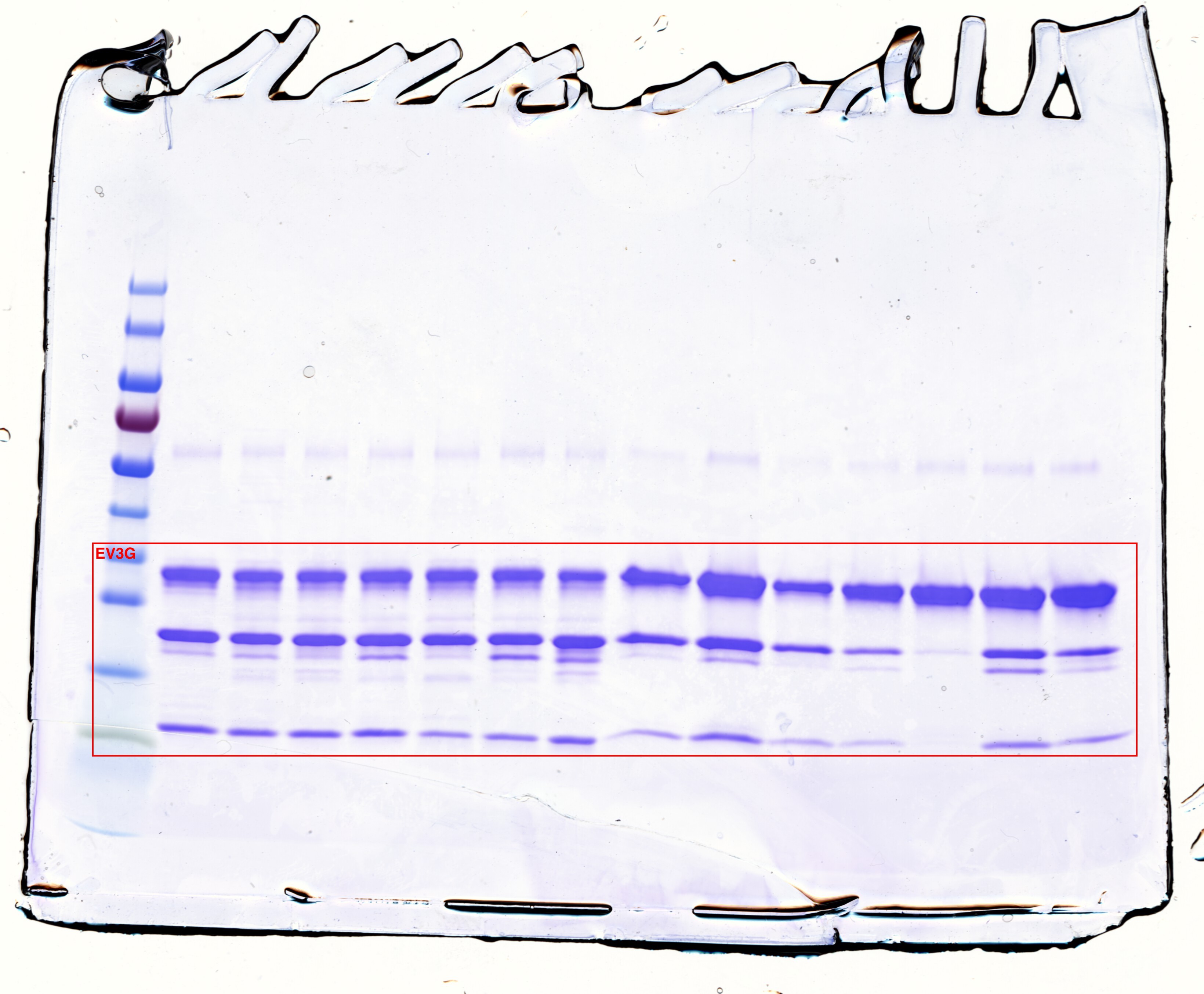

Supplement: Supplementary file 6 — Source Data for Expanded View [file EMBR-24-e57702-s010.zip › Figure_EV3/EV3G/Protein_gel_panel_G.jpg]

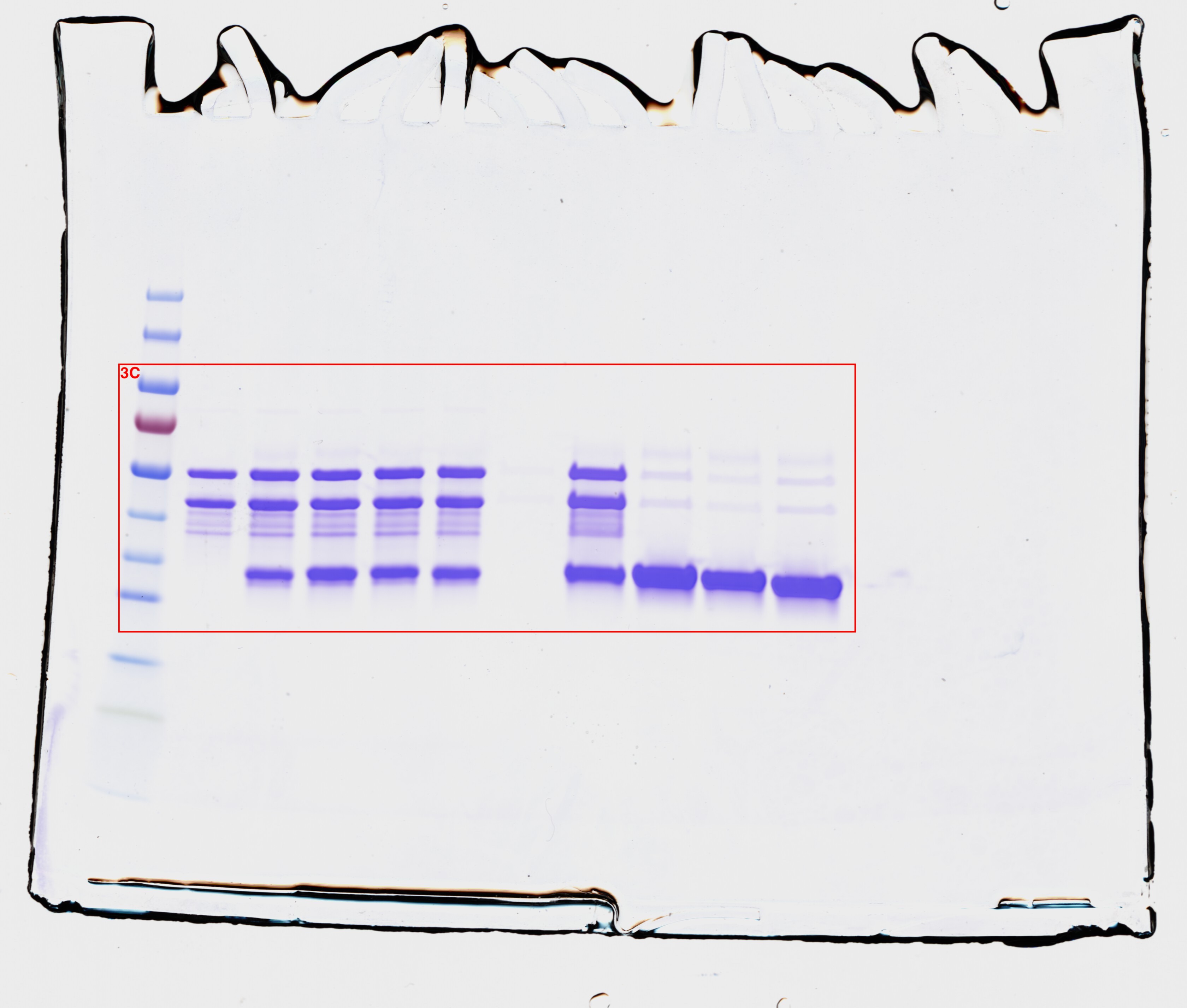

Supplement: Supplementary file 9 — Source Data for Figure 3 [file EMBR-24-e57702-s011.zip › Figure_3/3C/Protein_gel_panel_C.jpg]

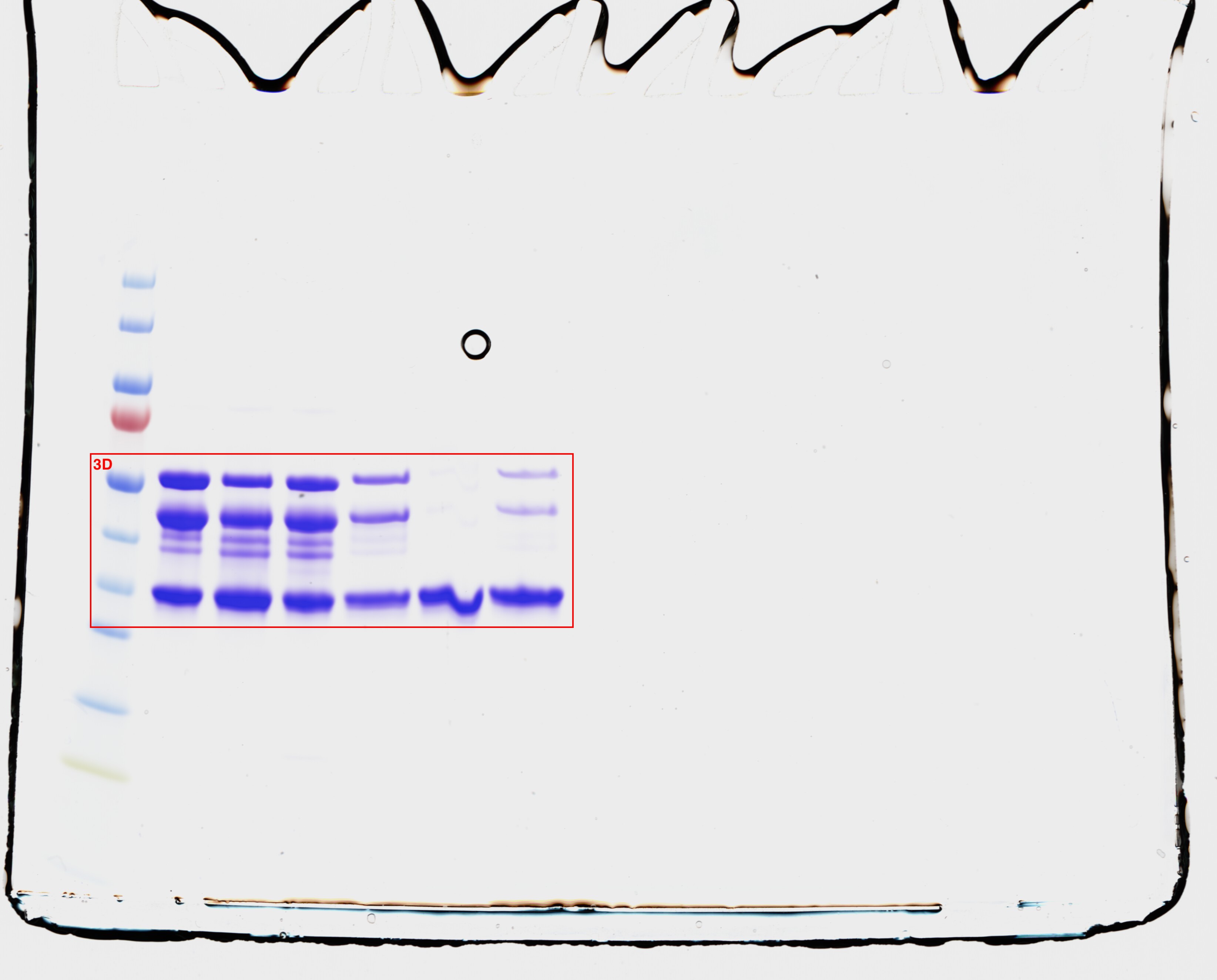

Supplement: Supplementary file 9 — Source Data for Figure 3 [file EMBR-24-e57702-s011.zip › Figure_3/3D/Protein_gel_panel D.jpg]

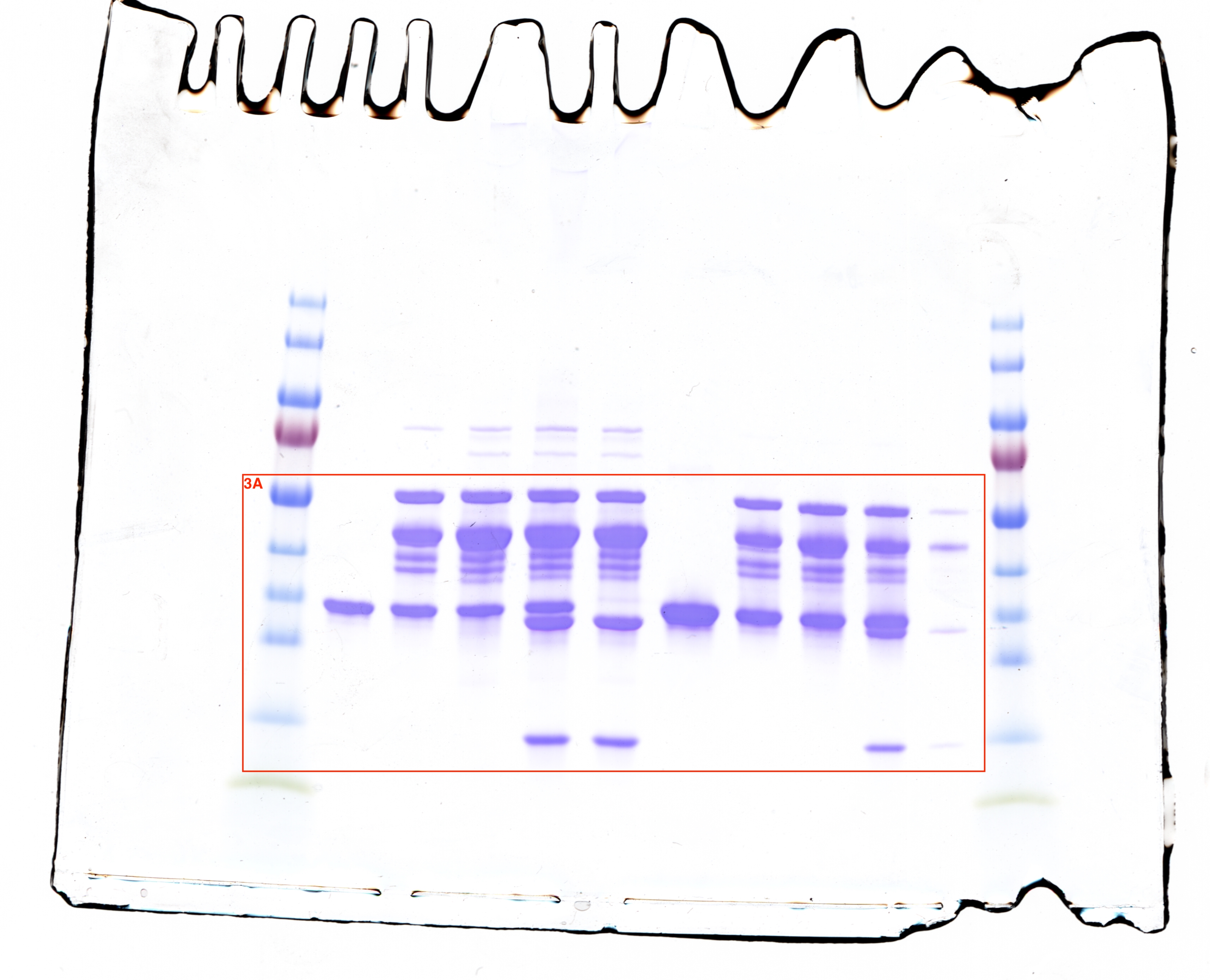

Supplement: Supplementary file 9 — Source Data for Figure 3 [file EMBR-24-e57702-s011.zip › Figure_3/3A/Protein_gel_panel_A.jpg]

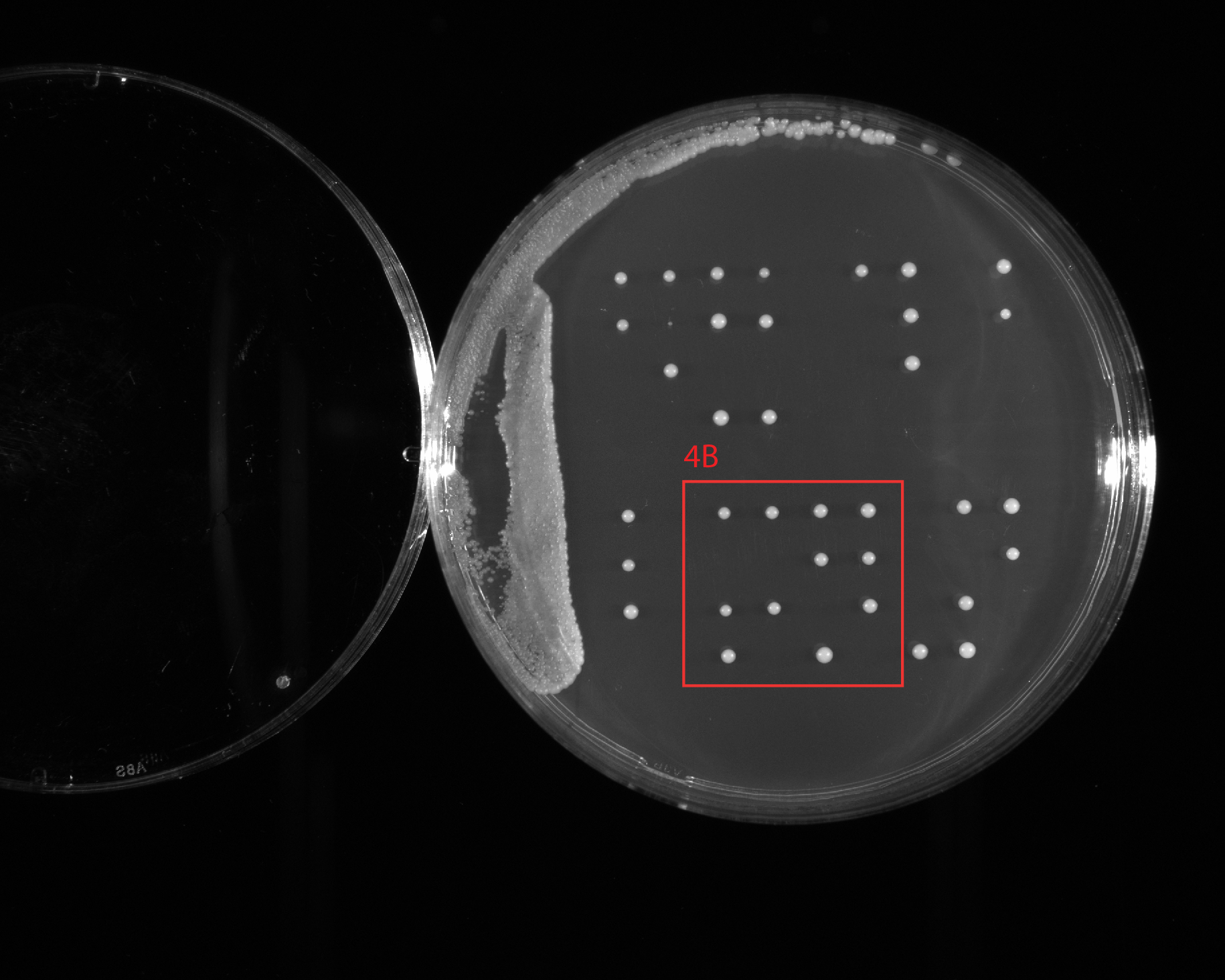

Supplement: Supplementary file 10 — Source Data for Figure 4 [file EMBR-24-e57702-s007.zip › Figure_4/4B/4B.tif]

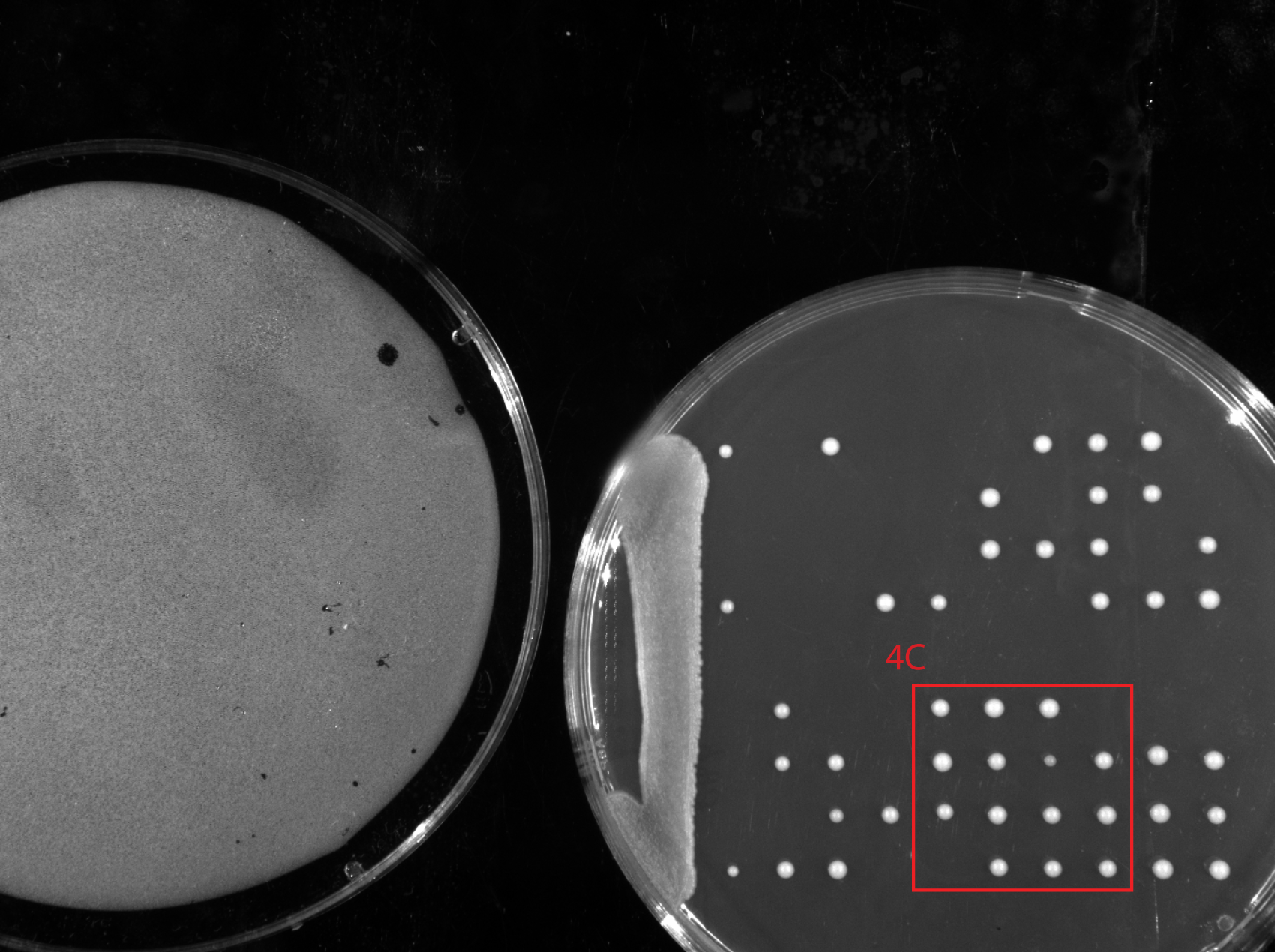

Supplement: Supplementary file 10 — Source Data for Figure 4 [file EMBR-24-e57702-s007.zip › Figure_4/4C/4C.tif]

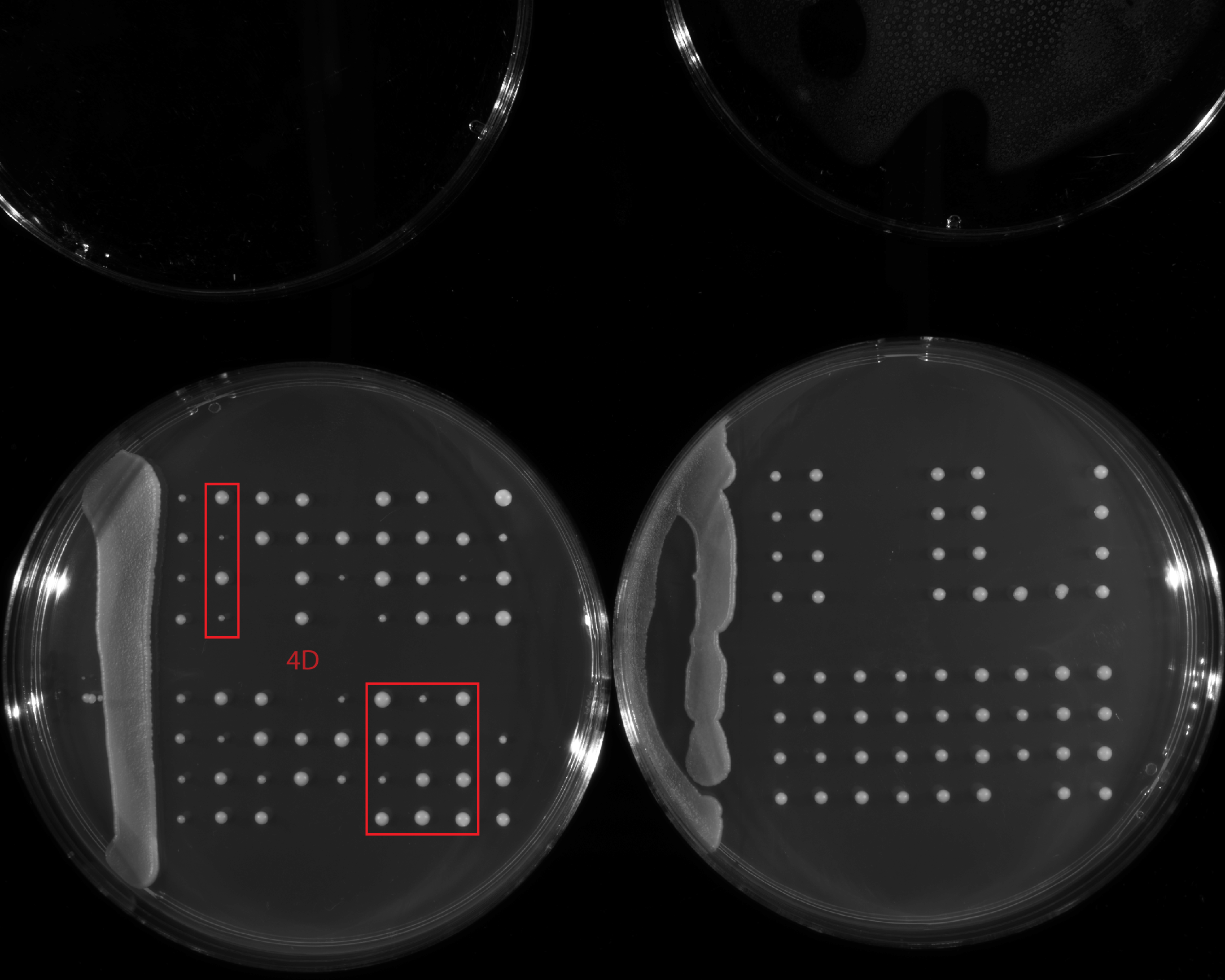

Supplement: Supplementary file 10 — Source Data for Figure 4 [file EMBR-24-e57702-s007.zip › Figure_4/4D/4D.tif]

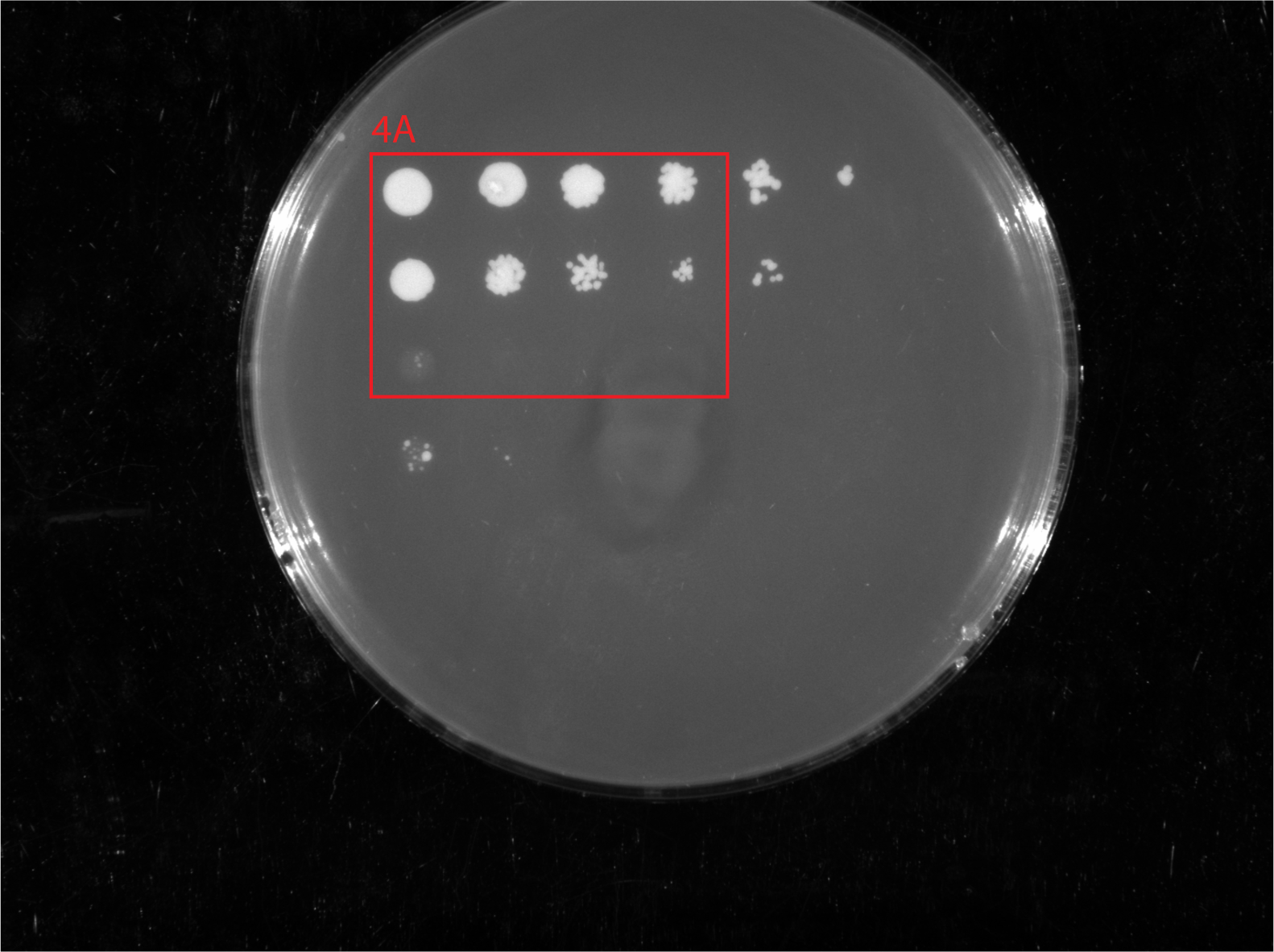

Supplement: Supplementary file 10 — Source Data for Figure 4 [file EMBR-24-e57702-s007.zip › Figure_4/4A/4A_right.tif]

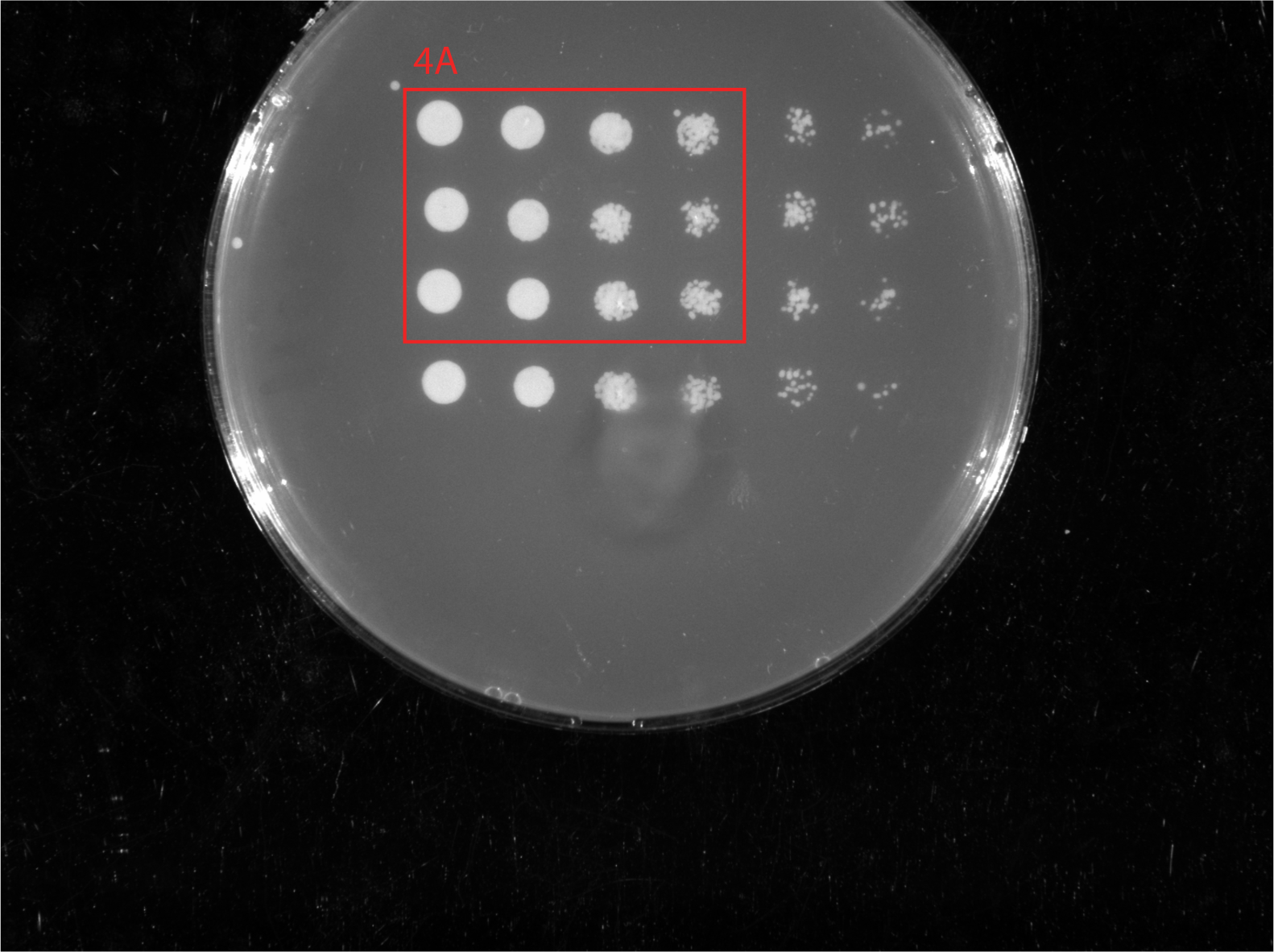

Supplement: Supplementary file 10 — Source Data for Figure 4 [file EMBR-24-e57702-s007.zip › Figure_4/4A/4A_left.tif]
