# Supplementary material for: Recognition of centromere‐specific histone Cse4 by the inner kinetochore Okp1‐Ame1 complex
Source: EMBO Rep. 2023 Nov 20;24(12):e57702. doi: 10.15252/embr.202357702 (PMC10702835; doi:10.15252/embr.202357702)
Supplement: Supplementary file 7 — Source Data for Figure 1 [file EMBR-24-e57702-s001.zip › Figure_1/1C/sch5_15_xia2.html]

# xia2 processing report

- Summary
- Dataset NATIVE
- Output files

## Merging statistics


**Space group:** P 42 21 2 (No. 94)
  
**Unit cell:** (154.305, 154.305, 37.114, 90, 90, 90)
  

xia2 output


×

#### xia2.txt

```
Environment configuration...
Python =&gt; /programs/x86_64-linux/ccp4/8.0/ccp4-8.0/libexec/python3.7
CCTBX =&gt; /programs/x86_64-linux/ccp4/8.0/ccp4-8.0/lib/python3.7/site-packages
CCP4 =&gt; /programs/x86_64-linux/ccp4/8.0/ccp4-8.0
CCP4_SCR =&gt; /tmp/tmpe94je952
Starting directory: /nfs/userdocs/sch/sdeng/Syncr_APS_092122_OACse4/processing/SCH5_15
Working directory: /nfs/userdocs/sch/sdeng/Syncr_APS_092122_OACse4/processing/SCH5_15
Free space:        20061.38 GB
Host: sch-curie
Contact: xia2.support@gmail.com
XIA2 3.8.0-g3d57088-dials-3.8
DIALS 3.8.0-gdc8ae18-release
CCP4 8.0.004
Command line: xia2 pipeline=dials-aimless /nfs/userdocs/sch/sdeng/Syncr_APS_092122_OACse4/images/sdeng/runs/SCH5_15/
Project directory: /nfs/userdocs/sch/sdeng/Syncr_APS_092122_OACse4/processing/SCH5_15
-------------------- Spotfinding SWEEP1 --------------------
303468 spots found on 1800 images (max 7568 / bin)
*                                                           
********                                                    
***********                                                 
******************            ********                      
****************************************************        
********************************************************  **
************************************************************
************************************************************
************************************************************
************************************************************
1                         image                         1800
------------------- Autoindexing SWEEP1 --------------------
All possible indexing solutions:
tP 154.19 154.19  37.09  90.00  90.00  90.00
oC 218.04 218.19  37.10  90.00  90.00  90.00
oP  37.09 154.19 154.15  90.00  90.00  90.00
mC 218.19 218.31  37.12  90.00  90.06  90.00
mP  37.08 154.17 154.13  90.00  90.07  90.00
aP  37.11 154.27 154.23  90.03  90.06  90.03
Indexing solution:
tP 154.19 154.19  37.09  90.00  90.00  90.00
-------------------- Integrating SWEEP1 --------------------
Processed batches 2 to 1801
Standard Deviation in pixel range: 0.31 0.42
Integration status per image (60/record):
oooooooooooooooooooooooooooooooooooooooooooooooooooooooooooo
oooooooooooooooooooooooooooooooooooooooooooooooooooooooooooo
oooooooooooooooooooooooooooooooooooooooooooooooooooooooooooo
oooooooooooooooooooooooooooooooooooooooooooooooooooooooooooo
oooooooooooooooooooooooooooooooooooooooooooooooooooooooooooo
oooooooooooooooooooooooooooooooooooooooooooooooooooooooooooo
oooooooooooooooooooooooooooooooooooooooooooooooooooooooooooo
oooooooooooooooooooooooooooooooooooooooooooooooooooooooooooo
oooooooooooooooooooooooooooooooooooooooooooooooooooooooooooo
oooooooooooooooooooooooooooooooooooooooooooooooooooooooooooo
oooooooooooooooooooooooooooooooooooooooooooooooooooooooooooo
oooooooooooooooooooooooooooooooooooooooooooooooooooooooooooo
oooooooooooooooooooooooooooooooooooooooooooooooooooooooooooo
oooooooooooooooooooooooooooooooooooooooooooooooooooooooooooo
oooooooooooooooooooooooooooooooooooooooooooooooooooooooooooo
oooooooooooooooooooooooooooooooooooooooooooooooooooooooooooo
oooooooooooooooooooooooooooooooooooooooooooooooooooooooooooo
oooooooooooooooooooooooooooooooooooooooooooooooooooooooooooo
oooooooooooooooooooooooooooooooooooooooooooooooooooooooooooo
oooooooooooooooooooooooooooooooooooooooooooooooooooooooooooo
oooooooooooooooooooooooooooooooooooooooooooooooooooooooooooo
oooooooooooooooooooooooooooooooooooooooooooooooooooooooooooo
oooooooooooooooooooooooooooooooooooooooooooooooooooooooooooo
oooooooooooooooooooooooooooooooooooooooooooooooooooooooooooo
oooooooooooooooooooooooooooooooooooooooooooooooooooooooooooo
oooooooooooooooooooooooooooooooooooooooooooooooooooooooooooo
oooooooooooooooooooooooooooooooooooooooooooooooooooooooooooo
oooooooooooooooooooooooooooooooooooooooooooooooooooooooooooo
oooooooooooooooooooooooooooooooooooooooooooooooooooooooooooo
oooooooooooooooooooooooooooooooooooooooooooooooooooooooooooo
&quot;o&quot; =&gt; good        &quot;%&quot; =&gt; ok        &quot;!&quot; =&gt; bad rmsd
&quot;O&quot; =&gt; overloaded  &quot;#&quot; =&gt; many bad  &quot;.&quot; =&gt; weak
&quot;@&quot; =&gt; abandoned
Mosaic spread: 0.071 &lt; 0.071 &lt; 0.071
-------------------- Preparing DEFAULT ---------------------
Likely spacegroups:
P 42 21 2
Reindexing to first spacegroup setting: P 42 21 2 (h,k,l)
--------------------- Scaling DEFAULT ----------------------
Resolution for sweep NATIVE/SWEEP1: 1.73 (cc_half &gt; 0.3)
--------------------- Scaling DEFAULT ----------------------
------------------- Unit cell refinement -------------------
NATIVE: 154.31 154.31  37.11  90.00  90.00  90.00
Project: AUTOMATIC
Crystal: DEFAULT
Sequence: 
Wavelength name: NATIVE
Wavelength 0.97918
Sweeps:
SWEEP SWEEP1 [WAVELENGTH NATIVE]
TEMPLATE SCH5_15_1_######.cbf
DIRECTORY /nfs/userdocs/sch/sdeng/Syncr_APS_092122_OACse4/images/sdeng/runs/SCH5_15
IMAGES (USER) 1 to 1800
MTZ file: /nfs/userdocs/sch/sdeng/Syncr_APS_092122_OACse4/processing/SCH5_15/DEFAULT/NATIVE/SWEEP1/integrate/dials_integrated.mtz
For AUTOMATIC/DEFAULT/NATIVE                 Overall    Low     High
High resolution limit                           1.73    4.69    1.73
Low resolution limit                          109.11  109.33    1.76
Completeness                                  100.0   100.0    97.7
Multiplicity                                   26.4    24.0    26.6
I/sigma                                        16.9    53.6     1.0
Rmerge(I)                                     0.125   0.041   4.729
Rmerge(I+/-)                                  0.124   0.041   4.676
Rmeas(I)                                      0.128   0.042   4.818
Rmeas(I+/-)                                   0.129   0.042   4.850
Rpim(I)                                       0.025   0.008   0.915
Rpim(I+/-)                                    0.034   0.011   1.280
CC half                                       1.000   1.000   0.355
Wilson B factor                              27.750
Anomalous completeness                         99.9   100.0    97.9
Anomalous multiplicity                         14.1    14.0    13.9
Anomalous correlation                        -0.071  -0.131  -0.032
Anomalous slope                               0.856
dF/F                                          0.054
dI/s(dI)                                      0.676
Total observations                          1258712   63096   61251
Total unique                                  47590    2633    2301
Assuming spacegroup: P 42 21 2
Unit cell (with estimated std devs):
154.3050(3) 154.3050(3) 37.11397(12)
 90.0        90.0       90.0        
sca format:
Scaled reflections (NATIVE): /nfs/userdocs/sch/sdeng/Syncr_APS_092122_OACse4/processing/SCH5_15/DataFiles/AUTOMATIC_DEFAULT_scaled.sca
sca_unmerged format:
Scaled reflections (NATIVE): /nfs/userdocs/sch/sdeng/Syncr_APS_092122_OACse4/processing/SCH5_15/DataFiles/AUTOMATIC_DEFAULT_scaled_unmerged.sca
mtz_unmerged format:
Scaled reflections (NATIVE): /nfs/userdocs/sch/sdeng/Syncr_APS_092122_OACse4/processing/SCH5_15/DataFiles/AUTOMATIC_DEFAULT_scaled_unmerged.mtz
mtz format:
Scaled reflections: /nfs/userdocs/sch/sdeng/Syncr_APS_092122_OACse4/processing/SCH5_15/DataFiles/AUTOMATIC_DEFAULT_free.mtz
```

Close

#### Overall

|  | NATIVE |
| --- | --- |
| Wavelength (Å) | 0.97918 |
| Resolution range (Å) | 109.11 - 1.73 (1.76 - 1.73) |
| Completeness (%) | 99.99 (97.75) |
| Multiplicity | 26.45 (26.62) |
| CC-half | 0.9996 (0.3553) |
| I/sigma | 16.93 (0.95) |
| Rmerge(I) | 0.1254 (4.7288) |
| Anomalous completeness (%) | 99.89 (97.92) |
| Anomalous multiplicity | 14.06 (13.87) |

#### References

Winter, G. (2010) J. Appl. Cryst. 43, 186-190.
  
Winter, G. et al. (2018) Acta Cryst. D74, 85-97.
  
Evans, P. (2006) Acta Cryst. D62, 72-82.
  
Evans, P. R. and Murshudov, G. N. (2013) Acta Cryst. D69, 1204-1214.
  
Winn, M. D. et al. (2011) Acta Cryst. D67, 235-242.

## xia2 output files

#### Reflection data files

- ##### Mtz files (useful for CCP4 and Phenix)

  | Dataset | File name |
  | --- | --- |
  | All datasets | AUTOMATIC\_DEFAULT\_free.mtz |
  | NATIVE | AUTOMATIC\_DEFAULT\_scaled\_unmerged.mtz |
- ##### SCA files (useful for autoSHARP, etc.)

  | Dataset | File name |
  | --- | --- |
  | NATIVE | AUTOMATIC\_DEFAULT\_scaled.sca |
- ##### SCA unmerged files (useful for XPREP and SHELXC/D/E)

  | Dataset | File name |
  | --- | --- |
  | NATIVE | AUTOMATIC\_DEFAULT\_scaled\_unmerged.sca |
- ##### Other files

  | File name | Description |
  | --- | --- |
  | xia2.cif | Crystallographic information file |

#### Log files

## Detailed statistics for dataset NATIVE

#### Overall

|  | Overall | Low resolution | High resolution |
| --- | --- | --- | --- |
| **Resolution (Å)** | 109.11 - 1.73 | 109.33 - 4.69 | 1.76 - 1.73 |
| **Observations** | 1258712 | 63096 | 61251 |
| **Unique reflections** | 47590 | 2633 | 2301 |
| **Multiplicity** | 26.4 | 24.0 | 26.6 |
| **Completeness** | 99.99% | 100.00% | 97.75% |
| **Mean I/σ(I)** | 16.9 | 53.6 | 1.0 |
| **Rmerge** | 0.125 | 0.041 | 4.729 |
| **Rmeas** | 0.128 | 0.042 | 4.818 |
| **Rpim** | 0.025 | 0.008 | 0.915 |
| **CC½** | 1.000 | 1.000 | 0.355 |

#### Resolution shells

| Resolution (Å) | N(obs) | N(unique) | Multiplicity | Completeness | Mean I | Mean I/σ(I) | Rmerge | Rmeas | Rpim | Ranom | CC½ | CCano |
| --- | --- | --- | --- | --- | --- | --- | --- | --- | --- | --- | --- | --- |
| 109.33 - 4.69 | 63096 | 2633 | 23.96 | 100.00 | 387.0 | 53.6 | 0.041 | 0.042 | 0.008 | 0.014 | 1.000\* | -0.131 |
| 4.69 - 3.73 | 65940 | 2458 | 26.83 | 100.00 | 371.4 | 59.7 | 0.048 | 0.049 | 0.009 | 0.015 | 1.000\* | -0.095 |
| 3.73 - 3.25 | 68142 | 2453 | 27.78 | 100.00 | 186.7 | 46.2 | 0.067 | 0.069 | 0.013 | 0.021 | 0.999\* | -0.200 |
| 3.25 - 2.96 | 58661 | 2412 | 24.32 | 100.00 | 102.8 | 32.7 | 0.092 | 0.094 | 0.019 | 0.032 | 0.998\* | -0.182 |
| 2.96 - 2.74 | 61064 | 2393 | 25.52 | 100.00 | 69.6 | 27.4 | 0.116 | 0.118 | 0.023 | 0.040 | 0.998\* | -0.156 |
| 2.74 - 2.58 | 63388 | 2384 | 26.59 | 100.00 | 46.4 | 21.8 | 0.151 | 0.154 | 0.030 | 0.051 | 0.997\* | -0.079 |
| 2.58 - 2.45 | 64401 | 2368 | 27.20 | 100.00 | 33.8 | 17.8 | 0.196 | 0.199 | 0.038 | 0.065 | 0.996\* | -0.122 |
| 2.45 - 2.35 | 65017 | 2366 | 27.48 | 100.00 | 27.9 | 15.4 | 0.235 | 0.239 | 0.045 | 0.077 | 0.994\* | -0.097 |
| 2.35 - 2.26 | 66020 | 2382 | 27.72 | 100.00 | 22.3 | 12.9 | 0.298 | 0.303 | 0.057 | 0.093 | 0.990\* | -0.073 |
| 2.26 - 2.18 | 64998 | 2338 | 27.80 | 100.00 | 16.7 | 9.9 | 0.395 | 0.402 | 0.076 | 0.124 | 0.987\* | -0.015 |
| 2.18 - 2.11 | 55907 | 2379 | 23.50 | 100.00 | 13.0 | 7.3 | 0.498 | 0.509 | 0.104 | 0.174 | 0.972\* | -0.038 |
| 2.11 - 2.05 | 59026 | 2331 | 25.32 | 99.91 | 10.9 | 6.4 | 0.609 | 0.622 | 0.123 | 0.194 | 0.952\* | -0.026 |
| 2.05 - 2.00 | 62029 | 2378 | 26.08 | 100.00 | 7.8 | 4.8 | 0.842 | 0.858 | 0.167 | 0.261 | 0.941\* | -0.017 |
| 2.00 - 1.95 | 61788 | 2311 | 26.74 | 100.00 | 5.5 | 3.5 | 1.186 | 1.209 | 0.232 | 0.346 | 0.905\* | -0.046 |
| 1.95 - 1.90 | 63612 | 2375 | 26.78 | 100.00 | 4.0 | 2.7 | 1.590 | 1.620 | 0.309 | 0.475 | 0.841\* | -0.001 |
| 1.90 - 1.86 | 63217 | 2304 | 27.44 | 100.00 | 3.3 | 2.3 | 1.960 | 1.997 | 0.378 | 0.547 | 0.788\* | -0.015 |
| 1.86 - 1.83 | 64300 | 2373 | 27.10 | 100.00 | 2.5 | 1.7 | 2.595 | 2.644 | 0.501 | 0.714 | 0.693\* | 0.035 |
| 1.83 - 1.79 | 64454 | 2348 | 27.45 | 100.00 | 2.1 | 1.4 | 3.169 | 3.228 | 0.610 | 0.826 | 0.569\* | -0.000 |
| 1.79 - 1.76 | 62401 | 2303 | 27.10 | 100.00 | 1.6 | 1.1 | 4.249 | 4.328 | 0.818 | 1.021 | 0.485\* | -0.027 |
| 1.76 - 1.73 | 61251 | 2301 | 26.62 | 97.75 | 1.5 | 1.0 | 4.729 | 4.818 | 0.915 | 1.106 | 0.355\* | -0.032 |

\*significant at p=0.01

#### Xtriage

#### 9 checks passed

The intensity statistics look normal, indicating that the data are not twinned.


×

#### Wilson ratio and moments

```
Acentric reflections:


   <I^2>/<I>^2    :2.055   (untwinned: 2.000; perfect twin 1.500)
   <F>^2/<F^2>    :0.783   (untwinned: 0.785; perfect twin 0.885)
   <|E^2 - 1|>    :0.750   (untwinned: 0.736; perfect twin 0.541)

Centric reflections:


   <I^2>/<I>^2    :3.098   (untwinned: 3.000; perfect twin 2.000)
   <F>^2/<F^2>    :0.649   (untwinned: 0.637; perfect twin 0.785)
   <|E^2 - 1|>    :0.966   (untwinned: 0.968; perfect twin 0.736)
```

Close

Translational NCS does not appear to be present.


×

#### Patterson analyses

```
 Largest Patterson peak with length larger than 15 Angstrom:
 Frac. coord.              :    0.023    0.023    0.500
 Distance to origin        :   19.247
 Height relative to origin :    4.619 %
 p_value(height)           :    9.643e-01

Explanation
 The p-value, the probability that a peak of the specified height or larger
 is found in a Patterson function of a macromolecule that does not have any
 translational pseudo-symmetry, is equal to 9.643e-01.  p_values smaller than
 0.05 might indicate weak translational pseudo symmetry, or the self vector of
 a large anomalous scatterer such as Hg, whereas values smaller than 1e-3 are
 a very strong indication for the presence of translational pseudo symmetry.
```

Close

Ice rings do not appear to be present.


×

#### Ice ring related problems

```
 The following statistics were obtained from ice-ring insensitive resolution
 ranges:

    mean bin z_score      : 2.52
        ( rms deviation   : 2.06 )
    mean bin completeness : 0.95
        ( rms deviation   : 0.08 )

 The following table shows the Wilson plot Z-scores and completeness for
 observed data in ice-ring sensitive areas.  The expected relative intensity
 is the theoretical intensity of crystalline ice at the given resolution.
 Large z-scores and high completeness in these resolution ranges might
 be a reason to re-assess your data processsing if ice rings were present.

  -------------------------------------------------------------
  | d_spacing | Expected rel. I | Data Z-score | Completeness |
  -------------------------------------------------------------
  |     3.897 |      1.000      |    0.92      |    1.00      |
  |     3.669 |      0.750      |    4.20      |    1.00      |
  |     3.441 |      0.530      |    4.27      |    1.00      |
  |     2.671 |      0.170      |    2.45      |    0.99      |
  |     2.249 |      0.390      |    0.31      |    0.97      |
  |     2.072 |      0.300      |    2.28      |    0.97      |
  |     1.948 |      0.040      |    7.04      |    0.88      |
  |     1.918 |      0.180      |    3.99      |    0.87      |
  |     1.883 |      0.030      |    1.12      |    0.86      |
  -------------------------------------------------------------
 Abnormalities in mean intensity or completeness at resolution ranges with a
 relative ice ring intensity lower than 0.10 will be ignored.
 No ice ring related problems detected.
 If ice rings were present, the data does not look worse at ice ring related
 d_spacings as compared to the rest of the data set.
```

Close

The fraction of outliers in the data is less than 0.1%.


×

#### Possible outliers

```
  Inspired by: Read, Acta Cryst. (1999). D55, 1759-1764

Acentric reflections:
            None

Centric reflections:
            None
```

Close

The data are not significantly anisotropic.


×

#### Maximum likelihood anisotropic Wilson scaling

```
ML estimate of overall B_cart value:
  27.02,  0.00,  0.00
         27.02,  0.00
                29.40

Equivalent representation as U_cif:
   0.34, -0.00, -0.00
          0.34,  0.00
                 0.37

Eigen analyses of B-cart:
  -------------------------------------------------
  | Eigenvector | Value   | Vector                |
  -------------------------------------------------
  | 1           |  29.404 | ( 0.00,  0.00,  1.00) |
  | 2           |  27.016 | (-0.71,  0.71, -0.00) |
  | 3           |  27.016 | ( 0.71,  0.71, -0.00) |
  -------------------------------------------------
ML estimate of  -log of scale factor:
  -3.12
```

Close

The resolution limit of the data seems appropriate.


×

#### Dataset consistency

```
  -------------------------------------------------------------------
  | Statistics for dataset consistency                              |
  |-----------------------------------------------------------------|
  | 1/d**2   | R-merge  | R-meas   | R-pim    | CC1/2    | CC(anom) |
  |-----------------------------------------------------------------|
  |   3.73   | 0.045    | 0.045    | 0.009    | 0.015    | -0.147   |
  |   2.96   | 0.075    | 0.077    | 0.015    | 0.025    | -0.191   |
  |   2.58   | 0.130    | 0.133    | 0.026    | 0.044    | -0.131   |
  |   2.35   | 0.214    | 0.218    | 0.041    | 0.071    | -0.089   |
  |   2.18   | 0.339    | 0.345    | 0.065    | 0.106    | -0.060   |
  |   2.05   | 0.550    | 0.562    | 0.113    | 0.183    | -0.007   |
  |   1.95   | 0.984    | 1.003    | 0.194    | 0.295    | -0.037   |
  |   1.86   | 1.757    | 1.790    | 0.340    | 0.507    | -0.026   |
  |   1.79   | 2.859    | 2.913    | 0.551    | 0.766    | 0.010    |
  |   1.73   | 4.478    | 4.562    | 0.865    | 1.061    | -0.020   |
  -------------------------------------------------------------------

References:
  Diederichs K & Karplus PA (1997) Nature Structural Biology 4:269-275
    (with erratum in: Nat Struct Biol 1997 Jul;4(7):592)
  Weiss MS (2001) J Appl Cryst 34:130-135.
  Dauter, Z. (2006). Acta Cryst. D62, 867-876.
  Karplus PA & Diederichs K (2012) Science 336:1030-3.
```

Close

The resolution cutoff appears to be similar in all directions.


×

#### Analysis of resolution limits

```
Your data have been examined to determine the resolution limits of the data
along the reciprocal space axes (a*, b*, and c*).  These are expected to vary
slightly depending on unit cell parameters and overall resolution, but should
never be significantly different for complete data.  (This is distinct from the
amount of anisotropy present in the data, which changes the effective
resolution but does not actually exclude reflections.)

    overall d_min                = 1.729
    d_min along a*               = 1.734
    d_min along b*               = 1.734
    d_min along c*               = 1.767
    max. difference between axes = 0.034

Resolution limits are within expected tolerances.
```

Close

The overall completeness in low-resolution shells is at least 90%.


×

#### Low resolution completeness analyses

```
The following table shows the completeness of the data to 5.0 A.  Poor
low-resolution completeness often leads to map distortions and other
difficulties, and is typically caused by problems with the crystal orientation
during data collection, overexposure of frames, interference with the beamstop,
or omission of reflections by data-processing software.
  -----------------------------------------------------------
  | Resolution range    | N(obs)/N(possible) | Completeness |
  -----------------------------------------------------------
  | 109.1361 -  10.7705 | [260/260]          | 1.000        |
  |  10.7705 -   8.5500 | [229/229]          | 1.000        |
  |   8.5500 -   7.4695 | [212/212]          | 1.000        |
  |   7.4695 -   6.7866 | [221/221]          | 1.000        |
  |   6.7866 -   6.3003 | [207/207]          | 1.000        |
  |   6.3003 -   5.9288 | [224/224]          | 1.000        |
  |   5.9288 -   5.6319 | [211/213]          | 0.991        |
  |   5.6319 -   5.3868 | [200/200]          | 1.000        |
  |   5.3868 -   5.1794 | [214/214]          | 1.000        |
  |   5.1794 -   5.0007 | [209/209]          | 1.000        |
  -----------------------------------------------------------
```

Close

The completeness is 99.88%.


×

#### Summary

```
The original dataset contained unmerged intensities; the statistics below
are for the merged data.
              File name:                                 None
            Data labels:                                 None
            Space group:                            P 42 21 2
              Unit cell: 154.305, 154.305, 37.114, 90, 90, 90
              Data type:                       xray.intensity
             Resolution:                     109.11 - 1.72919
              Anomalous:                                False
  Number of reflections:                                47535
           Completeness:                               99.88%

  Completeness should be used to determine if there is sufficient data for
  refinement and/or model-building. A value greater than 90% is generally
  desired, while a value less than 75% is considered poor. Values in between
  will provide less than optimal results.
```

Close

Zwart, P. H., Grosse-Kunstleve, R. W. & Adams, P. D. (2005). *CCP4 Newsl.* **43**, contribution 7.

## Analysis plots

#### Analysis by resolution

×

#### CC½ vs resolution

```
The correlation coefficients, CC½, between random half-datasets. A correlation
coefficient of +1 indicates good correlation, and 0 indicates no correlation.
CC½ is typically close to 1 at low resolution, falling off to close to zero at
higher resolution. A typical resolution cutoff based on CC½ is around 0.3-0.5.

[1] Karplus, P. A., & Diederichs, K. (2012). Science, 336(6084), 1030-1033.
    https://doi.org/10.1126/science.1218231
[2] Diederichs, K., & Karplus, P. A. (2013). Acta Cryst D, 69(7), 1215-1222.
    https://doi.org/10.1107/S0907444913001121
[3] Evans, P. R., & Murshudov, G. N. (2013). Acta Cryst D, 69(7), 1204-1214.
    https://doi.org/10.1107/S0907444913000061
```

Close

#### Analysis by batch

#### Miscellaneous
