# Supplementary material for: Recognition of centromere‐specific histone Cse4 by the inner kinetochore Okp1‐Ame1 complex
Source: EMBO Rep. 2023 Nov 20;24(12):e57702. doi: 10.15252/embr.202357702 (PMC10702835; doi:10.15252/embr.202357702)
Supplement: Supplementary file 7 — Source Data for Figure 1 [file EMBR-24-e57702-s001.zip › Figure_1/1C/D_1000273421_val-report-full_P1 (8T0P).pdf]

Preliminary Full wwPDB X-ray Structure Validation Report (i)

Aug 14, 2023 – 04:46 PM EDT

Deposition ID : D_1000273421

This wwPDB validation report is NOT for manuscript review

This is a Preliminary Full wwPDB X-ray Structure Validation Report.

This report is produced by the wwPDB Deposition System during initial deposition but before annotation of the structure.

We welcome your comments at *validation@mail.wwpdb.org*
A user guide is available at
https://www.wwpdb.org/validation/2017/XrayValidationReportHelp
with specific help available everywhere you see the (i) symbol.

The types of validation reports are described at
http://www.wwpdb.org/validation/2017/FAQs#types.

The following versions of software and data (see references (i)) were used in the production of this report:

MolProbity	:	4.02b-467
Mogul	:	1.8.5 (274361), CSD as541be (2020)
Xtriage (Phenix)	:	1.13
EDS	:	2.35
Percentile statistics	:	20191225.v01 (using entries in the PDB archive December 25th 2019)
Refmac	:	5.8.0158
CCP4	:	7.0.044 (Gargrove)
Ideal geometry (proteins)	:	Engh & Huber (2001)
Ideal geometry (DNA, RNA)	:	Parkinson et al. (1996)
Validation Pipeline (wwPDB-VP)	:	2.35

Not For Manuscript Review

1 Overall quality at a glance ⓘ

The following experimental techniques were used to determine the structure:
X-RAY DIFFRACTION

The reported resolution of this entry is 1.73 Å.

Percentile scores (ranging between 0-100) for global validation metrics of the entry are shown in the following graphic. The table shows the number of entries on which the scores are based.

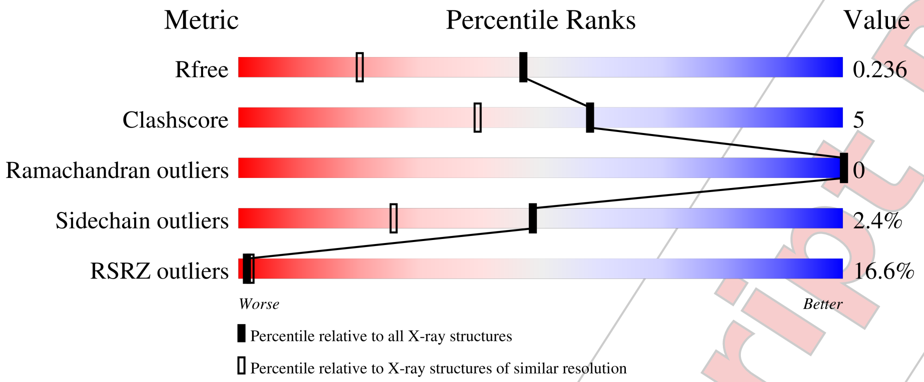

Metric	Whole archive (#Entries)	Similar resolution (#Entries, resolution range(Å))
R_{free}	130704	3764 (1.76-1.72)
Clashscore	141614	3923 (1.76-1.72)
Ramachandran outliers	138981	3878 (1.76-1.72)
Sidechain outliers	138945	3878 (1.76-1.72)
RSRZ outliers	127900	3705 (1.76-1.72)

The table below summarises the geometric issues observed across the polymeric chains and their fit to the electron density. The red, orange, yellow and green segments of the lower bar indicate the fraction of residues that contain outliers for >=3, 2, 1 and 0 types of geometric quality criteria respectively. A grey segment represents the fraction of residues that are not modelled. The numeric value for each fraction is indicated below the corresponding segment, with a dot representing fractions <=5% The upper red bar (where present) indicates the fraction of residues that have poor fit to the electron density. The numeric value is given above the bar.

Mol	Chain	Length	Quality of chain
1	B	107	15% / 89% 10% •
2	A	152	18% / 85% 14% •
3	C	18	17% / 83% 17%

Not For Manuscript Review

2 Entry composition (i)

There are 5 unique types of molecules in this entry. The entry contains 2492 atoms, of which 0 are hydrogens and 0 are deuteriums.

In the tables below, the ZeroOcc column contains the number of atoms modelled with zero occupancy, the AltConf column contains the number of residues with at least one atom in alternate conformation and the Trace column contains the number of residues modelled with at most 2 atoms.

- Molecule 1 is a protein called Inner kinetochore subunit AME1.

Mol	Chain	Residues	Atoms					ZeroOcc	AltConf	Trace
			Total	C	N	O	S			
1	B	107	867	548	144	173	2	0	0	0

- Molecule 2 is a protein called Inner kinetochore subunit OKP1.

Mol	Chain	Residues	Atoms					ZeroOcc	AltConf	Trace
			Total	C	N	O	S			
2	A	152	1270	807	229	231	3	0	1	0

There are 2 discrepancies between the modelled and reference sequences:

Chain	Residue	Modelled	Actual	Comment	Reference
A	123	GLY	-	expression tag	UNP P53298
A	124	SER	-	expression tag	UNP P53298

- Molecule 3 is a protein called Histone H3-like centromeric protein CSE4.

Mol	Chain	Residues	Atoms				ZeroOcc	AltConf	Trace
			Total	C	N	O			
3	C	18	140	83	30	27	0	0	0

- Molecule 4 is SULFATE ION (three-letter code: SO4) (formula: O_4S).

Not For Manuscript Review

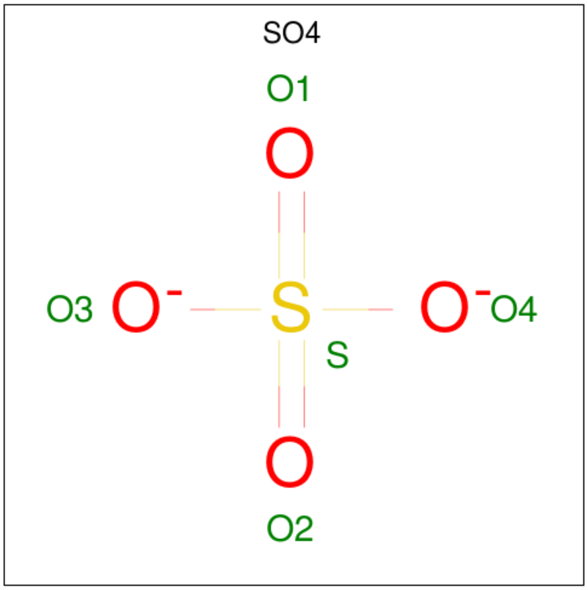

Mol	Chain	Residues	Atoms	ZeroOcc	AltConf
4	D	1	Total O S<br>5 4 1	0	0
4	D	1	Total O S<br>5 4 1	0	0
4	D	1	Total O S<br>5 4 1	0	0
4	D	1	Total O S<br>5 4 1	0	0
4	D	1	Total O S<br>5 4 1	0	0
4	D	1	Total O S<br>5 4 1	0	0

- Molecule 5 is water.

Mol	Chain	Residues	Atoms	ZeroOcc	AltConf
5	S	185	Total O<br>185 185	0	0

Not For Manuscript Review

3 Residue-property plots (i)

These plots are drawn for all protein, RNA, DNA and oligosaccharide chains in the entry. The first graphic for a chain summarises the proportions of the various outlier classes displayed in the second graphic. The second graphic shows the sequence view annotated by issues in geometry and electron density. Residues are color-coded according to the number of geometric quality criteria for which they contain at least one outlier: green = 0, yellow = 1, orange = 2 and red = 3 or more. A red dot above a residue indicates a poor fit to the electron density (RSRZ > 2). Stretches of 2 or more consecutive residues without any outlier are shown as a green connector. Residues present in the sample, but not in the model, are shown in grey.

- Molecule 1: Inner kinetochore subunit AME1

Chain B:

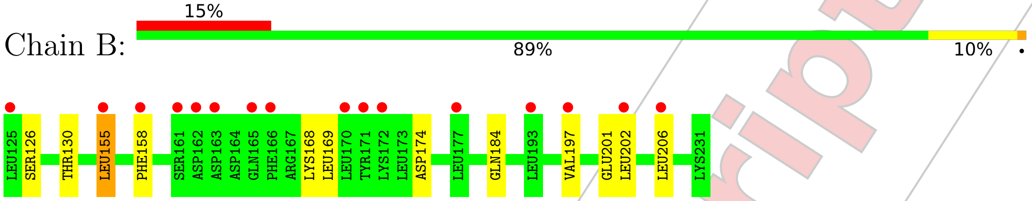

- Molecule 2: Inner kinetochore subunit OKP1

Chain A:

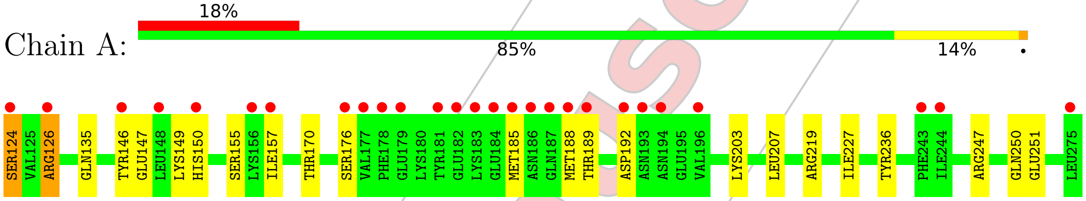

- Molecule 3: Histone H3-like centromeric protein CSE4

Chain C:

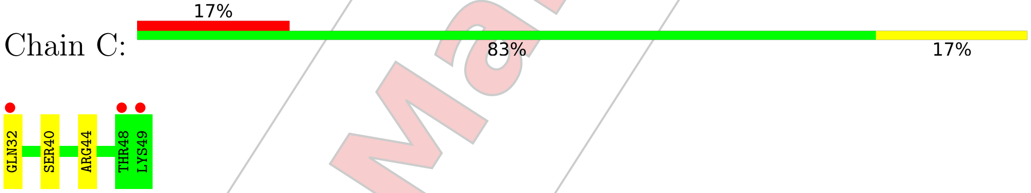

Not For Manuscript Review

4 Data and refinement statistics (i)

Property	Value	Source
Space group	P 42 21 2	Depositor
Cell constants<br>a, b, c, α, β, γ	154.31Å 154.31Å 37.11Å<br>90.00° 90.00° 90.00°	Depositor
Resolution (Å)	69.01 – 1.73<br>69.01 – 1.73	Depositor<br>EDS
% Data completeness<br>(in resolution range)	99.8 (69.01-1.73)<br>99.8 (69.01-1.73)	Depositor<br>EDS
R_{merge}	0.13	Depositor
R_{sym}	(Not available)	Depositor
$< I/\sigma(I) >$ [1]	1.23 (at 1.73Å)	Xtriage
Refinement program	PHENIX (1.20.1_4487: ???)	Depositor
R, R_{free}	0.207 , 0.236<br>0.209 , 0.236	Depositor<br>DCC
R_{free} test set	2443 reflections (5.14%)	wwPDB-VP
Wilson B-factor (Å^2)	30.8	Xtriage
Anisotropy	0.054	Xtriage
Bulk solvent k_{sol}(e/Å^3), B_{sol}(Å^2)	0.37 , 49.4	EDS
L-test for twinning[2]	$< \|L\| > = 0.49$, $< L^2 > = 0.32$	Xtriage
Estimated twinning fraction	No twinning to report.	Xtriage
F_o,F_c correlation	0.96	EDS
Total number of atoms	2492	wwPDB-VP
Average B, all atoms (Å^2)	46.0	wwPDB-VP

Xtriage's analysis on translational NCS is as follows: *The largest off-origin peak in the Patterson function is 4.57% of the height of the origin peak. No significant pseudotranslation is detected.*

[1]Intensities estimated from amplitudes.

[2]Theoretical values of $< |L| >$, $< L^2 >$ for acentric reflections are 0.5, 0.333 respectively for untwinned datasets, and 0.375, 0.2 for perfectly twinned datasets.

Not For Manuscript Review

Not For Manuscript Review

5 Model quality (i)

5.1 Standard geometry (i)

Bond lengths and bond angles in the following residue types are not validated in this section: SO4

The Z score for a bond length (or angle) is the number of standard deviations the observed value is removed from the expected value. A bond length (or angle) with $|Z| > 5$ is considered an outlier worth inspection. RMSZ is the root-mean-square of all Z scores of the bond lengths (or angles).

Mol	Chain	Bond lengths		Bond angles	
		RMSZ	#$\|Z\|$ >5	RMSZ	#$\|Z\|$ >5
1	B	0.80	1/874 (0.1%)	0.85	2/1179 (0.2%)
2	A	0.81	1/1293 (0.1%)	0.87	1/1737 (0.1%)
3	C	0.55	0/139	0.88	0/186
All	All	0.80	2/2306 (0.1%)	0.86	3/3102 (0.1%)

All (2) bond length outliers are listed below:

Mol	Chain	Res	Type	Atoms	Z	Observed(Å)	Ideal(Å)
2	A	155	SER	CB-OG	6.98	1.51	1.42
1	B	201	GLU	CB-CG	5.12	1.61	1.52

All (3) bond angle outliers are listed below:

Mol	Chain	Res	Type	Atoms	Z	Observed(^{o})	Ideal(^{o})
1	B	202	LEU	CB-CG-CD1	-5.27	102.04	111.00
2	A	236	TYR	CB-CG-CD1	-5.20	117.88	121.00
1	B	197	VAL	CA-CB-CG2	-5.00	103.39	110.90

There are no chirality outliers.

There are no planarity outliers.

5.2 Too-close contacts (i)

In the following table, the Non-H and H(model) columns list the number of non-hydrogen atoms and hydrogen atoms in the chain respectively. The H(added) column lists the number of hydrogen atoms added and optimized by MolProbity. The Clashes column lists the number of clashes within the asymmetric unit, whereas Symm-Clashes lists symmetry-related clashes.

Mol	Chain	Non-H	H(model)	H(added)	Clashes	Symm-Clashes
1	B	867	0	883	7	0
2	A	1270	0	1284	17	0
3	C	140	0	144	2	0
4	D	30	0	0	0	0
5	S	185	0	0	5	2
All	All	2492	0	2311	22	2

The all-atom clashscore is defined as the number of clashes found per 1000 atoms (including hydrogen atoms). The all-atom clashscore for this structure is 5.

All (22) close contacts within the same asymmetric unit are listed below, sorted by their clash magnitude.

Atom-1	Atom-2	Interatomic distance (Å)	Clash overlap (Å)
3:C:32:GLN:N	3:C:32:GLN:OE1	2.21	0.73
2:A:219:ARG:NH2	5:S:107:HOH:O	2.30	0.65
1:B:158:PHE:O	1:B:168:LYS:NZ	2.29	0.65
2:A:149:LYS:HG2	2:A:150:HIS:ND1	2.15	0.61
2:A:251:GLU:OE2	5:S:149:HOH:O	2.18	0.56
2:A:170:THR:HG23	2:A:207:LEU:HD13	1.93	0.51
2:A:124:SER:O	2:A:126:ARG:HD2	2.10	0.50
1:B:206:LEU:HD21	2:A:247:ARG:HD3	1.93	0.50
2:A:189:THR:OG1	2:A:192:ASP:HB3	2.12	0.49
2:A:126:ARG:HD2	2:A:126:ARG:N	2.28	0.48
2:A:185:MET:O	2:A:189:THR:HG22	2.14	0.47
1:B:169:LEU:HD22	2:A:188:MET:HG3	1.97	0.46
1:B:155:LEU:HD23	1:B:155:LEU:HA	1.74	0.46
2:A:250:GLN:NE2	5:S:154:HOH:O	2.26	0.46
1:B:184:GLN:HG3	5:S:151:HOH:O	2.15	0.46
1:B:130:THR:HG22	2:A:227:ILE:HG22	1.99	0.44
1:B:174:ASP:OD1	2:A:203:LYS:NZ	2.40	0.44
2:A:147:GLU:H	2:A:147:GLU:HG2	1.62	0.44
3:C:40:SER:OG	3:C:44:ARG:NH1	2.51	0.42
2:A:135:GLN:HG2	5:S:104:HOH:O	2.19	0.42
2:A:146:TYR:O	2:A:149:LYS:HB3	2.20	0.42
2:A:149:LYS:HG2	2:A:150:HIS:CE1	2.55	0.41

All (2) symmetry-related close contacts are listed below. The label for Atom-2 includes the symmetry operator and encoded unit-cell translations to be applied.

Atom-1	Atom-2	Interatomic distance (Å)	Clash overlap (Å)
5:S:68:HOH:O	5:S:68:HOH:O[8_555]	1.90	0.30

Continued on next page...

Not For Manuscript Review

Continued from previous page...

Atom-1	Atom-2	Interatomic distance (Å)	Clash overlap (Å)
5:S:28:HOH:O	5:S:92:HOH:O[8_556]	2.18	0.02

5.3 Torsion angles (i)

5.3.1 Protein backbone (i)

In the following table, the Percentiles column shows the percent Ramachandran outliers of the chain as a percentile score with respect to all X-ray entries followed by that with respect to entries of similar resolution.

The Analysed column shows the number of residues for which the backbone conformation was analysed, and the total number of residues.

Mol	Chain	Analysed	Favoured	Allowed	Outliers	Percentiles	
1	B	105/107 (98%)	104 (99%)	1 (1%)	0	100	100
2	A	151/152 (99%)	149 (99%)	2 (1%)	0	100	100
3	C	16/18 (89%)	16 (100%)	0	0	100	100
All	All	272/277 (98%)	269 (99%)	3 (1%)	0	100	100

There are no Ramachandran outliers to report.

5.3.2 Protein sidechains (i)

In the following table, the Percentiles column shows the percent sidechain outliers of the chain as a percentile score with respect to all X-ray entries followed by that with respect to entries of similar resolution.

The Analysed column shows the number of residues for which the sidechain conformation was analysed, and the total number of residues.

Mol	Chain	Analysed	Rotameric	Outliers	Percentiles	
1	B	103/105 (98%)	101 (98%)	2 (2%)	57	36
2	A	137/146 (94%)	133 (97%)	4 (3%)	42	18
3	C	15/16 (94%)	15 (100%)	0	100	100
All	All	255/267 (96%)	249 (98%)	6 (2%)	49	26

All (6) residues with a non-rotameric sidechain are listed below:

Not For Manuscript Review

Mol	Chain	Res	Type
1	B	126	SER
1	B	155	LEU
2	A	124	SER
2	A	126	ARG
2	A	157	ILE
2	A	176	SER

Sometimes sidechains can be flipped to improve hydrogen bonding and reduce clashes. All (1) such sidechains are listed below:

Mol	Chain	Res	Type
3	C	32	GLN

5.3.3 RNA (i)

There are no RNA molecules in this entry.

5.4 Non-standard residues in protein, DNA, RNA chains (i)

There are no non-standard protein/DNA/RNA residues in this entry.

5.5 Carbohydrates (i)

There are no monosaccharides in this entry.

5.6 Ligand geometry (i)

6 ligands are modelled in this entry.

In the following table, the Counts columns list the number of bonds (or angles) for which Mogul statistics could be retrieved, the number of bonds (or angles) that are observed in the model and the number of bonds (or angles) that are defined in the Chemical Component Dictionary. The Link column lists molecule types, if any, to which the group is linked. The Z score for a bond length (or angle) is the number of standard deviations the observed value is removed from the expected value. A bond length (or angle) with $|Z| > 2$ is considered an outlier worth inspection. RMSZ is the root-mean-square of all Z scores of the bond lengths (or angles).

Mol	Type	Chain	Res	Link	Bond lengths			Bond angles		
					Counts	RMSZ	$\#\|Z\| > 2$	Counts	RMSZ	$\#\|Z\| > 2$
4	SO4	D	18	-	4,4,4	0.23	0	6,6,6	0.93	0
4	SO4	D	8	-	4,4,4	0.21	0	6,6,6	0.33	0
4	SO4	D	9	-	4,4,4	0.15	0	6,6,6	0.23	0

Not For Manuscript Review

Mol	Type	Chain	Res	Link	Bond lengths			Bond angles		
					Counts	RMSZ	#|Z| > 2	Counts	RMSZ	#|Z| > 2
4	SO4	D	20	-	4,4,4	0.98	0	6,6,6	1.66	1 (16%)
4	SO4	D	2	-	4,4,4	0.20	0	6,6,6	1.40	1 (16%)
4	SO4	D	3	-	4,4,4	0.18	0	6,6,6	0.85	0

There are no bond length outliers.

All (2) bond angle outliers are listed below:

Mol	Chain	Res	Type	Atoms	Z	Observed(^{o})	Ideal(^{o})
4	D	20	SO4	O4-S-O3	3.83	125.39	109.06
4	D	2	SO4	O3-S-O2	-2.66	95.42	109.31

There are no chirality outliers.

There are no torsion outliers.

There are no ring outliers.

No monomer is involved in short contacts.

5.7 Other polymers (i)

There are no such residues in this entry.

5.8 Polymer linkage issues (i)

There are no chain breaks in this entry.

Not For Manuscript Review

6 Fit of model and data (i)

6.1 Protein, DNA and RNA chains (i)

In the following table, the column labelled '#RSRZ> 2' contains the number (and percentage) of RSRZ outliers, followed by percent RSRZ outliers for the chain as percentile scores relative to all X-ray entries and entries of similar resolution. The OWAB column contains the minimum, median, 95th percentile and maximum values of the occupancy-weighted average B-factor per residue. The column labelled 'Q< 0.9' lists the number of (and percentage) of residues with an average occupancy less than 0.9.

Mol	Chain	Analysed	<RSRZ>	#RSRZ>2			OWAB(Å^2)	Q<0.9
1	B	107/107 (100%)	1.05	16 (14%)	2	2	20, 36, 83, 98	0
2	A	152/152 (100%)	1.08	27 (17%)	1	1	20, 42, 91, 102	0
3	C	18/18 (100%)	0.82	3 (16%)	1	2	26, 37, 72, 86	0
All	All	277/277 (100%)	1.05	46 (16%)	1	2	20, 39, 88, 102	0

All (46) RSRZ outliers are listed below:

Mol	Chain	Res	Type	RSRZ
1	B	155	LEU	7.0
2	A	185	MET	6.2
2	A	177	VAL	5.8
2	A	178	PHE	5.4
1	B	163	ASP	5.2
2	A	157	ILE	4.9
3	C	48	THR	4.7
2	A	181	TYR	4.5
2	A	193	ASN	3.9
1	B	162	ASP	3.9
2	A	275	LEU	3.7
2	A	146	TYR	3.5
1	B	177	LEU	3.3
2	A	186	ASN	3.3
2	A	124	SER	3.3
2	A	187	GLN	3.3
2	A	179	GLU	3.2
2	A	126	ARG	3.1
2	A	183	LYS	3.1
2	A	196	VAL	3.1
2	A	182	GLU	2.9
2	A	194	ASN	2.8
1	B	158	PHE	2.7

Continued on next page...

Not For Manuscript Review

Continued from previous page...

Mol	Chain	Res	Type	RSRZ
1	B	161	SER	2.7
2	A	189	THR	2.7
2	A	176	SER	2.6
2	A	192	ASP	2.6
1	B	171	TYR	2.6
1	B	125	LEU	2.5
2	A	184	GLU	2.5
3	C	49	LYS	2.5
2	A	188	MET	2.5
2	A	244	ILE	2.5
3	C	32	GLN	2.5
1	B	202	LEU	2.3
1	B	193	LEU	2.3
1	B	170	LEU	2.3
1	B	172	LYS	2.3
2	A	148	LEU	2.2
1	B	166	PHE	2.2
2	A	150	HIS	2.2
1	B	206	LEU	2.2
2	A	243	PHE	2.1
2	A	156	LYS	2.1
1	B	165	GLN	2.1
1	B	197	VAL	2.1

6.2 Non-standard residues in protein, DNA, RNA chains (i)

There are no non-standard protein/DNA/RNA residues in this entry.

6.3 Carbohydrates (i)

There are no monosaccharides in this entry.

6.4 Ligands (i)

In the following table, the Atoms column lists the number of modelled atoms in the group and the number defined in the chemical component dictionary. The B-factors column lists the minimum, median, 95th percentile and maximum values of B factors of atoms in the group. The column labelled 'Q< 0.9' lists the number of atoms with occupancy less than 0.9.

Continued on next page...

Not For Manuscript Review

Continued from previous page...

Mol	Type	Chain	Res	Atoms	RSCC	RSR	B-factors(Å^2)	Q<0.9
4	SO4	D	20	5/?	0.91	0.34	30,30,30,30	0
4	SO4	D	9	5/?	0.93	0.24	100,103,110,116	0
4	SO4	D	8	5/?	0.94	0.18	57,64,75,93	0
4	SO4	D	3	5/?	0.95	0.13	48,53,57,57	0
4	SO4	D	2	5/?	0.98	0.14	41,43,52,57	0
4	SO4	D	18	5/?	0.99	0.12	31,33,47,56	0

6.5 Other polymers (i)

There are no such residues in this entry.

Not For Manuscript Review
