# Supplementary material for: Recognition of centromere‐specific histone Cse4 by the inner kinetochore Okp1‐Ame1 complex
Source: EMBO Rep. 2023 Nov 20;24(12):e57702. doi: 10.15252/embr.202357702 (PMC10702835; doi:10.15252/embr.202357702)
Supplement: Supplementary file 11 [file EMBR-24-e57702-s009.pdf]

EMBO reports

Recognition of centromere-specific histone Cse4 by the inner kinetochore Okp1-Ame1 complex

Sunbin Deng, Jiaxi Cai, Stephen Harrison, Huilin Zhou, and Stephen Hinshaw
DOI: 10.15252/embr.202357702

Corresponding author(s): Stephen Hinshaw (hinshaw@stanford.edu) , Stephen Harrison (harrison@crystal.harvard.edu), Huilin Zhou (huzhou@health.ucsd.edu)

Review Timeline:

Submission Date:	21st Jun 23
Editorial Decision:	26th Jul 23
Revision Received:	12th Sep 23
Editorial Decision:	26th Oct 23
Revision Received:	30th Oct 23
Accepted:	2nd Nov 23

Editor: Deniz Senyilmaz Tiebe

Transaction Report:

(Note: With the exception of the correction of typographical or spelling errors that could be a source of ambiguity, letters and reports are not edited. Depending on transfer agreements, referee reports obtained elsewhere may or may not be included in this compilation. Referee reports are anonymous unless the Referee chooses to sign their reports.)

1st Editorial Decision **26th Jul 2023**

Dear Dr. Hinshaw,

Thank you for the submission of your research manuscript to our journal, which was now seen by three referees, whose reports are copied below.

The referees express interest in the provided crystal structure of a Cse4/CENP-A bound to the Okp1-Ame1 heterodimer and the subsequent analyses. However, they also raise significant concerns that need to be addressed to consider publication here.

Given these positive recommendations, we would like to invite you to submit a revised manuscript. Please revise your manuscript with the understanding that the referee concerns (as in their reports) must be fully addressed and their suggestions taken on board. Please address all referee concerns in a complete point-by-point response. Acceptance of the manuscript will depend on a positive outcome of a second round of review. It is EMBO reports policy to allow a single round of major experimental revision only and acceptance or rejection of the manuscript will therefore depend on the completeness of your responses included in the next, final version of the manuscript.

We realize that it is difficult to revise to a specific deadline. In the interest of protecting the conceptual advance provided by the work, we recommend a revision within 3 months. Please discuss the revision progress ahead of this time with me if you require more time to complete the revisions, or if you have questions or comments regarding the revision (also by video chat).

IMPORTANT NOTE: we perform an initial quality control of all revised manuscripts before re-review. Your manuscript will FAIL this control and the handling will be DELAYED if the following APPLIES:
1. A data availability section providing access to data deposited in public databases is missing (where applicable).
2. Your manuscript contains statistics and error bars based on n=2. Please use scatter plots in these cases.

You can submit the revision either as a Scientific Report or as a Research Article. For Scientific Reports, the revised manuscript can contain up to 5 main figures and 5 Expanded View figures, and it should not exceed 27000 characters. If the revision leads to a manuscript with more than 5 main figures it will be published as a Research Article. In this case the Results and Discussion section should be separate. If a Scientific Report is submitted, these sections have to be combined. This will help to shorten the manuscript text by eliminating some redundancy that is inevitable when discussing the same experiments twice. In either case, all materials and methods should be included in the main manuscript file.

When submitting your revised manuscript, please carefully review the instructions that follow below. Failure to include requested items will delay the evaluation of your revision.

1) a .docx formatted version of the manuscript text (including legends for main figures, EV figures and tables). Please make sure that the changes are highlighted to be clearly visible.

2) individual production quality figure files as .eps, .tif, .jpg (one file per figure). See https://wol-prod-cdn.literatumonline.com/pb-assets/embo-site/EMBOPress_Figure_Guidelines_061115-1561436025777.pdf for more info on how to prepare your figures.

3) We replaced Supplementary Information with Expanded View (EV) Figures and Tables that are collapsible/expandable online. A maximum of 5 EV Figures can be typeset. EV Figures should be cited as 'Figure EV1, Figure EV2" etc... in the text and their respective legends should be included in the main text after the legends of regular figures.

- For the figures that you do NOT wish to display as Expanded View figures, they should be bundled together with their legends in a single PDF file called *Appendix*, which should start with a short Table of Content. Appendix figures should be referred to in the main text as: "Appendix Figure S1, Appendix Figure S2" etc. See detailed instructions regarding expanded view here: <https://www.embopress.org/page/journal/14693178/authorguide#expandedview>

- Additional Tables/Datasets should be labeled and referred to as Table EV1, Dataset EV1, etc. Legends have to be provided in a separate tab in case of .xls files. Alternatively, the legend can be supplied as a separate text file (README) and zipped together with the Table/Dataset file.

4) a .docx formatted letter INCLUDING the reviewers' reports and your detailed point-by-point responses to their comments. As part of the EMBO publication's Transparent Editorial Process, EMBO reports publishes online a Review Process File (RPF) to accompany accepted manuscripts. This File will be published in conjunction with your paper and will include the referee reports, your point-by-point response and all pertinent correspondence relating to the manuscript.
https://www.embopress.org/page/journal/14693178/authorguide#transparentprocess
You are able to opt out of this by letting the editorial office know (emboreports@embo.org). If you do opt out, the Review Process File link will point to the following statement: "No Review Process File is available with this article, as the authors have

chosen not to make the review process public in this case."

5) a complete author checklist, which you can download from our author guidelines https://www.embopress.org/page/journal/14693178/authorguide. Please insert information in the checklist that is also reflected in the manuscript. The completed author checklist will also be part of the RPF.

6) Please note that all corresponding authors are required to supply an ORCID ID for their name upon submission of a revised manuscript (<https://orcid.org/>). Please find instructions on how to link your ORCID ID to your account in our manuscript tracking system in our Author guidelines
<https://www.embopress.org/page/journal/14693178/authorguide#authorshipguidelines>

7) Before submitting your revision, primary datasets produced in this study need to be deposited in an appropriate public database (see https://www.embopress.org/page/journal/14693178/authorguide#datadeposition). Please remember to provide a reviewer password if the datasets are not yet public. The accession numbers and database should be listed in a formal "Data Availability" section placed after Materials & Method (see also https://www.embopress.org/page/journal/14693178/authorguide#datadeposition). Please note that the Data Availability Section is restricted to new primary data that are part of this study. * Note - All links should resolve to a page where the data can be accessed. *
If your study has not produced novel datasets, please mention this fact in the Data Availability Section.

8) At EMBO Press we ask authors to provide source data for the main figures. Our source data coordinator will contact you to discuss which figure panels we would need source data for and will also provide you with helpful tips on how to upload and organize the files.

Additional information on source data and instruction on how to label the files are available:
https://www.embopress.org/page/journal/14693178/authorguide#sourcedata

9) Our journal encourages inclusion of *data citations in the reference list* to directly cite datasets that were re-used and obtained from public databases. Data citations in the article text are distinct from normal bibliographical citations and should directly link to the database records from which the data can be accessed. In the main text, data citations are formatted as follows: "Data ref: Smith et al, 2001" or "Data ref: NCBI Sequence Read Archive PRJNA342805, 2017". In the Reference list, data citations must be labeled with "[DATASET]". A data reference must provide the database name, accession number/identifiers and a resolvable link to the landing page from which the data can be accessed at the end of the reference. Further instructions are available at http://www.embopress.org/page/journal/14693178/authorguide#referencesformat

10) Regarding data quantification (see Figure Legends:
https://www.embopress.org/page/journal/14693178/authorguide#figureformat)

The following points must be specified in each figure legend:

- the name of the statistical test used to generate error bars and P values,

- the number (n) of independent experiments (please specify technical or biological replicates) underlying each data point,

- the nature of the bars and error bars (s.d., s.e.m.),

- If the data are obtained from n Program fragment delivered error ``Can't locate object method "less" via package "than" (perhaps you forgot to load "than"?) at //ejpvfs23/sites23b/embor_www/letters/embor_decision_revise_and_review.txt line 56.' 2, use scatter blots showing the individual data points.

Discussion of statistical methodology can be reported in the materials and methods section, but figure legends should contain a basic description of n, P and the test applied.

- Please also include scale bars in all microscopy images.

11) The journal requires a statement specifying whether or not authors have competing interests (defined as all potential or actual interests that could be perceived to influence the presentation or interpretation of an article). In case of competing interests, this must be specified in your disclosure statement. Further information: https://www.embopress.org/competing-interests

12) Please also note our reference format:
http://www.embopress.org/page/journal/14693178/authorguide#referencesformat

We would also welcome the submission of cover suggestions, or motifs to be used by our Graphics Illustrator in designing a cover.

I look forward to seeing a revised version of your manuscript when it is ready. Please let me know if you have questions or comments regarding the revision.

Kind regards,

Deniz Senyilmaz Tiebe

Deniz Senyilmaz Tiebe, PhD
Editor
EMBO Reports

Referee #1:

This manuscript by Sunbin Deng and colleagues focuses on the S. cerevisiae kinetochore and reports a structure of the Okp1-Ame1 heterodimer in complex with an essential N-terminal segment (named the END segment) of Cse4/CENP-A, a histone H3 variant that acts as a beacon for kinetochore assembly. Okp1-Ame1 are products of essential kinetochore genes of S. cerevisiae, and are subunits of a larger assembly named the Ctf19 complex. Recent structural work on the S. cerevisiae Ctf19 complex bound to a hybrid Cse4 nucleosome failed to visualize the interaction of Cse4 with Okp1-Ame1. Rather than questioning the importance of the interaction, failure to visualize it, and more generally failure so far in identifying how the Ctf19 complex specifically recognizes Cse4, calls for further studies on the topic. This manuscript is therefore timely and has the potential to shed new light on an important issue in kinetochore biology.

The authors report a crystal structure of a minimal subcomplex of Okp1:Ame1 bound to a Cse4 peptide. They validate this interaction biochemically. Finally, they demonstrate that a mutant affecting two crucial residues in the END region is unable to support viability of S. cerevisiae, and that mutants affecting residues in Okp1 and Ame1 implicated in Cse4 END binding cannot sustain growth, or do so minimally.

Collectively, this is a technically sound study. All major conclusions are well supported. The narrative is concise and effective. The study sheds light on an important missing element of the S. cerevisiae kinetochore, a widely studied model system for kinetochore assembly and function. There has been substantial recent progress in the structural analysis of the Ctf19 complex. This progress, however, has not yet been followed up by an understanding of how the Ctf19 complex interacts with the Cse4 nucleosome. This study rationalizes that some of these recent contributions may not explain all facts satisfactorily. I therefore support publication of this study in EMBO Reports, but would urge the authors to consider the following points.

1) Page 2: The authors write "Nkp1 and Nkp2 bind an adjacent site in the assembled Ctf19c, but it is not clear from comparison of the cryo-EM and crystal structures (Fig. S1C) whether they would need to partly dissociate from Okp1-Ame1 to accommodate Cse4-END." This reads cryptic. Is it because the conformational change relative to the EM structure renders a prediction of steric clash uncertain? If so, maybe consider moving this short sentence to the paragraph on page 3 starting with "Comparison with cryo-EM structures..." and please try explain the context for this question more clearly.

2) The authors could elect to clarify if the binding affinity for the END peptide of the minimal heterodimeric complex used for crystallization (Okp1-125-275/Ame1-124-231) identical to that of the Okp1-Ame1 heterodimer. A more interesting extension would be measuring END peptide binding by the Ctf19 complex (not simply COMA). This would clarify if the inability to visualize the END peptide in complex with Ctf19 is due to an intrinsic property of the fully assembled Ctf19 complex or rather a limit of the structural analysis.

3) As pointed out in the Discussion, which is concise but effective, the work discussed here has important implications for our understanding of how the Ctf19 complex decodes the Cse4 histone. I would like to urge the authors to try to provide graphical aid to illustrate this concept, as most readers will find it challenging to understand what does not fit with the previous Ctf19c-nucleosome structures. As figures 3 and 4 are conceptually related, they could be merged and a new Figure 4 assembled to illustrate these important concepts graphically.

Minor points

Page 2: The statement "Molecular recognition of Cse4 by the Okp1-Ame1 heterodimer has not been resolved, despite recent cryo-EM structures that show in detail nearly all protein contacts that contribute to inner kinetochore assembly" should be referenced.

Please indicate model used in GraphPad Prism for binding reactions with the END peptide

"Cse4 residues 28-60 (Cse4END) was cloned into a pLIC..." Technically it is the DNA sequence encoding such segment that is being cloned.

Referee #2:

Part of the N-terminal tail of Cse4 (28-60), the centromere specifying histone in S. cerevisiae, is essential for cell viability. Several studies have shown that this part of the tail directly binds Okp1-Ame1 proteins, which in turn interact with Ndc80, a microtubule-binding protein, to enable chromosome segregation during mitosis. Anedchenko et al. proposed that this interaction is regulated by posttranslational modifications at positions R37 and K49 on Cse4. Here, Deng et al. provide a high-resolution molecular insight into this important interaction. Their work uncovers an Okp1-Ame1 conformation that is distinct from the conformation previously observed in the context of the Ctf19 complex and the Ctif19-Cse4 complex. This is an interesting observation that provides potential new elements for understanding and interpreting the structural organization of the S. cerevisiae centromere.
The results are interesting, but the work needs more rigor in the experimental part, in the presentation of the data, and in the writing.

1. Electron density.
- Show the differential electron density for Cse4 when it is omitted from the model, and final electron density when it is included and refined with the model. Show also where is Cse4 density located in the crystal lattice and if crystal contact can influence interpretation.
- Page 2, last paragraph "Most ordered Cse4 residues in the crystal structure have a-helical character (residues 34-46), and there are short flexible extensions (residues 32-34 and 46-49)." - please illustrate with the figure in the supplementary information
- It is very confusing to show density from an experiment done in different study in Figure 1. Please remove this from main set of figures.
2. Clear analysis of the possibility of Cse4 and Nkp1/2 co-binding to Okp1-Ame1 complex.
- some of the Ame1 residues that interact with Cse4 also interact with Nkp1/2 in the Ctf19 and Ctf19-Cse4 complexes (for example, Ile195). This is not addressed in the figure or text. Figure 2, last paragraph "Nkp1 and Nkp2 bind an adjacent site in the assembled Ctf19c, but it is not clear from comparison of the cryo-EM and crystal structures (Fig. S1C) whether they would need to partly dissociate from Okp1-Ame1 to accommodate Cse4END . Aside from Nkp1 and Nkp2, the Cse4 END binding site is exposed and on an external surface in structures of the assembled Ctf19c (4, 16)." What does this mean? Would there be steric clashes between Cse4 and Nkp1/2? Figure S1C is not clear.
- Competition experiment with Cse4 and Nkp1/2 was done only in the context of the full length Okp1-Ame1 (that has more extensive interaction interface with Nkp1/2) and not with the constructs used in crystallography. Also, in the context of the FL proteins, it would be nice to first form Okp1-Ame1-Nkp1/2 and then titrate in Cse4 to see if it will replace or co-bind together with Nkp1/2 or it wouldn't bind at all.
3. Check whether the post-translational modifications identified by Anedchenko et al. affect binding. This can be easily addressed with a fluorescence polarization experiment. Also comment on their finding that Okp1-R164C suppresses the Cse4-R37A mutation.
4. Pull-down experiments with GST-Cse4END should include GST only control.

Minor comments:
1. Abstract should have more info on the context and significance of the work.
2. The discussion should be more organized and flow better. It is also nice if you illustrate proposed model with a cartoon. Speculate about Cnn1/Wip1 binding and compare with human CCAN structure.
3. Figure 1A is nice, but it is not clear why are some regions in darker colors (doesn't correspond to names indicated above). Also, it is not very clear from the figure what are the constructs used in the study.
4. Figures 2B and C illustrating interactions should be bigger (and Cse4 should be in the map ideally).
5. The AF2 model is OK to mention, but interpretation of the data should focus on comparison with other experimental structures. Also, the compatibility of the observed Okp1-Ame1 conformation with the Cse4 nucleosome within the Ctf19-Cse4 complex should be illustrated by a figure.
6. The authors should be aware that their results, although interesting and significant, were still obtained with truncated proteins and isolated from other components that form yeast centromeres. Therefore, they provide valuable additional information that complements previously published structures and opens the possibility of a different protein arrangement in the yeast centromere, rather than "enables us to reinterpret published models of centromere- kinetochore".
7. Figures are not corresponding to the text. Page 1, last paragraph; Page 2, paragraph 5.
9. Page 2, paragraph 2. "Okp1 and Ame1 are the only fully essential components of the Ctf19c." Provide reference and explain.
10. Page 4, paragraph 2. Fig. 5A should be Fig 4A.
11. All statements based on previous data should contain corresponding references.

Referee #3:

The kinetochore is a large protein complex, which mediates accurate chromosome segregation through bridging centromeric chromatin with spindle microtubules. Centromeric chromatin contains a special nucleosome including Cse4p (Budding yeast) or CENP-A (human), which are centromere specific-histone H3 variant. Therefore, it is important for kinetochore components to distinguish centromeric chromatin from canonical H3 containing nucleosomes. Authors in this MS used budding yeast kinetochore components and tried to address how they specifically recognize Cse4p. They focused on the Cse4p binding to the Okp1-Ame1 complex and determined a crystal structure of the Okp1-Ame1 complex bound with Cse4p N-terminal peptide. They found that Cse4p N-terminal peptide bound a junction of Head domain and coiled-coil of the Okp1-Ame1 complex. They further identified several critical amino acids for robust binding of Cse4p to the Okp1-Ame1 complex. Mutation of these amino acids resulted in binding defects of Cse4p to the Okp1-Ame1 complex in vitro and caused cell death in vivo. Then, authors proposed that this binding is a key initial step for kinetochore assembly on the Cse4p containing centromeric chromatin.
Recent years, a cryo-EM structure of the entire kinetochore complex including Cse4p and the Okp1-Ame1 complex has been solved. However, based on the Cryo-EM structure, it was unclear how the kinetochore complex such as the Okp1-Ame1 complex recognizes the Cse4 nucleosome, because clear binding of Cse4p to the Okp1-Ame1 complex was not observed in the cryo-EM structure. However, as binding of Cse4p to the Okp1-Ame1 complex was observed in vitro experiments, it was open question to address how the kinetochore complex specifically recognizes Cse4p from canonical H3 nucleosomes. Authors tackled this question in this paper. Data quality in this MS is relatively high and this reviewer notices that this kind of piece of experiment is important to complement the cryo-EM structure. Therefore, I basically support the publication of this work in EMBO Rep. However, I have some suggestions to improve the paper, and authors should consider for incorporation of my suggestions in the revised version.

1. Concerning Nkp1-2, authors mentioned that the Cse4-Ame-Okp1-Nkp1-2 complex can be formed (Figure 3). Authors also described that Cse4 binding affinity to the Ame1-Okp1 complex to similar even in the absence of the Nkp1-2 complex. However, in Figure 3 experiments biding affinity seems to be slightly decreased in the absence of the Nkp1-2 complex (Figure 3B). I think that they can show more quantitative presentation for binding in either presence or absence of the Nkp1-2 complex. KDs for these bindings can be presented in either presence or absence of the Nkp1-2 complex.
2. For in vivo experiment, it might be better to add data for Cse4 lacking END sequence. We can easily compare phenotype of Cse4-2A or 1A and Cse4 END deletion.
3. In this paper authors used Cse4 peptide, but I am curious to know whether Cse4 N-terminal region binds to the Ame-Okp1 complex in the nucleosome context. If authors show binding results using Cse4 Nucleosome, it would be better.
4. In discussion, it would be good to discuss the Mif2 pathway. Cse4A-1A mutation showed slow growth, but cells with this mutation were still viable. In vitro experiments in Figure 3C indicated that both 1A and 2A mutations did not binds to the Okp1-Ame1 complex ar similar extent. Considering these results, we suggest that there would be the redundant pathway to link the kinetochore with centromeric chromatin in vivo. Mif2 is a good candidate to link the kinetochore with centromeric chromatin. It would be good to discuss this point.

Minor points
In page 4, for Figure describing CSE4 mutation, authors indicated Fig5A. It should be Fig 4A.

September 12th, 2023

To the editors at EMBO Reports:

We have updated our manuscript, *Recognition of centromere-specific histone Cse4 by the inner kinetochore Okp1-Ame1 complex* according to comments from reviewers. The updated manuscript and figure files are included in this resubmission. Changes to the main text are highlighted. The following pages have our in-line responses to all reviewer comments.

In addition to carrying out biochemical experiments as requested by reviewers, we have made several changes to the text, which we think have clarified the manuscript substantially. The new or repeated experiments are fully consistent with our original interpretations and solidify these points. We have also added a schematic figure (new Figure EV5), which we hope will help readers understand the connections between our new structural findings and the many cell biology and genetics results it addresses. We have also added several citations to further bring these connections to the reader's attention.

Thank you for a constructive review process. We look forward to hearing from you.

Regards,
Stephen M. Hinshaw

Referee #1:

This manuscript by Sunbin Deng and colleagues focuses on the S. cerevisiae kinetochore and reports a structure of the Okp1-Ame1 heterodimer in complex with an essential N-terminal segment (named the END segment) of Cse4/CENP-A, a histone H3 variant that acts as a beacon for kinetochore assembly. Okp1-Ame1 are products of essential kinetochore genes of S. cerevisiae, and are subunits of a larger assembly named the Ctf19 complex. Recent structural work on the S. cerevisiae Ctf19 complex bound to a hybrid Cse4 nucleosome failed to visualize the interaction of Cse4 with Okp1-Ame1. Rather than questioning the importance of the interaction, failure to visualize it, and more generally failure so far in identifying how the Ctf19 complex specifically recognizes Cse4, calls for further studies on the topic. This manuscript is therefore timely and has the potential to shed new light on an important issue in kinetochore biology.

The authors report a crystal structure of a minimal subcomplex of Okp1:Ame1 bound to a Cse4 peptide. They validate this interaction biochemically. Finally, they demonstrate that a mutant affecting two crucial residues in the END region is unable to support viability of S. cerevisiae, and that mutants affecting residues in Okp1 and Ame1 implicated in Cse4 END binding cannot sustain growth, or do so minimally.

Collectively, this is a technically sound study. All major conclusions are well supported. The narrative is concise and effective. The study sheds light on an important missing element of the S. cerevisiae kinetochore, a widely studied model system for kinetochore assembly and function. There has been substantial recent progress in the structural analysis of the Ctf19 complex. This progress, however, has not yet been followed up by an understanding of how the Ctf19 complex interacts with the Cse4 nucleosome. This study rationalizes that some of these recent contributions may not explain all facts satisfactorily. I therefore support publication of this study in EMBO Reports, but would urge the authors to consider the following points.

1) Page 2: The authors write "Nkp1 and Nkp2 bind an adjacent site in the assembled Ctf19c, but it is not clear from comparison of the cryo-EM and crystal structures (Fig. S1C) whether they would need to partly dissociate from Okp1-Ame1 to accommodate Cse4-END." This reads cryptic. Is it because

the conformational change relative to the EM structure renders a prediction of steric clash uncertain? If so, maybe consider moving this short sentence to the paragraph on page 3 starting with "Comparison with cryo-EM structures..." and please try explain the context for this question more clearly.

We have moved this sentence to the suggested position and modified the wording so that the statement is clearer. Making this change has also clarified the rationale for the pulldown experiment shown in Figure 3A.

2) The authors could elect to clarify if the binding affinity for the END peptide of the minimal heterodimeric complex used for crystallization (Okp1-125-275/Ame1-124-231) identical to that of the Okp1-Ame1 heterodimer. A more interesting extension would be measuring END peptide binding by the Ctf19 complex (not simply COMA). This would clarify if the inability to visualize the END peptide in complex with Ctf19 is due to an intrinsic property of the fully assembled Ctf19 complex or rather a limit of the structural analysis.

We have carried out FP experiments with the truncated AO complex used for crystallization. The measured dissociation is ~750 nM. We have inserted a short paragraph describing this experiment in the main text and include the experiment in the new EV figures. The buffer composition required to make these measurements differed for that used in the experiments with full length AO due to the limited solubility of truncated AO in low salt. We have updated the methods section to reflect this difference.

While we were preparing these revisions, the Barford group published a supplemental figure (Fig. S12, PMID 37506202) showing that Cse4-END does not bind the assembled Ctf19c in a gel filtration experiment. In our own unpublished work, we found that an immobilized MBP-Cse4-N peptide binds recombinant Ctf19c weakly if at all, though these experiments were not sufficiently clear to report in a publication. Our updated manuscript includes a reference to the Barford lab's finding.

We do not know why Ctf19c cannot bind Cse4-END. First, recombinant Ctf19c, when purified from insect cells, is phosphorylated on multiple subunits, and it is not yet clear how these modifications might influence the various biochemical activities of the Ctf19c. Second, the tendency of the full Ctf19c to dimerize in a state that apparently locks the AO head domains in a conformation not observed in the current crystal structure (see Hinshaw and Harrison, 2019 for details) complicates potential Cse4-END binding experiments. We believe this dimer form to be a biochemical detail of the reconstitution that is not relevant to kinetochore assembly *in vivo*. An ideal experiment would enable quantification of Ctf19c-nucleosome engagement with the mutations we have mapped in the current study, as this would presumably bypass the Ctf19c dimerization problem. We cannot perform this ideal experiment yet, as we do not know how to reconstitute a biologically relevant Ctf19c-nucleosome complex.

3) As pointed out in the Discussion, which is concise but effective, the work discussed here has important implications for our understanding of how the Ctf19 complex decodes the Cse4 histone. I would like to urge the authors to try to provide graphical aid to illustrate this concept, as most readers will find it challenging to understand what does not fit with the previous Ctf19c-nucleosome structures. As figures 3 and 4 are conceptually related, they could be merged and a new Figure 4 assembled to illustrate these important concepts graphically.

We have included a graphical representation as suggested. We have included this illustration as an Expanded View figure for interested or curious readers.

Minor points

Page 2: The statement "Molecular recognition of Cse4 by the Okp1-Ame1 heterodimer has not been resolved, despite recent cryo-EM structures that show in detail nearly all protein contacts that contribute to inner kinetochore assembly" should be referenced.

We have included the following references: Dendooven, 2023; Guan, 2021; Hinshaw, 2019; Hinshaw, 2020; Yan, 2018; Yan, 2019.

Please indicate model used in GraphPad Prism for binding reactions with the END peptide

We have included this information in the updated Methods section.

"Cse4 residues 28-60 (Cse4END) was cloned into a pLIC..." Technically it is the DNA sequence encoding such segment that is being cloned.

We have fixed this statement as requested.

Referee #2:

Part of the N-terminal tail of Cse4 (28-60), the centromere specifying histone in S. cerevisiae, is essential for cell viability. Several studies have shown that this part of the tail directly binds Okp1-Ame1 proteins, which in turn interact with Ndc80, a microtubule-binding protein, to enable chromosome segregation during mitosis. Anedchenko et al. proposed that this interaction is regulated by posttranslational modifications at positions R37 and K49 on Cse4. Here, Deng et al. provide a high-resolution molecular insight into this important interaction. Their work uncovers an Okp1-Ame1 conformation that is distinct from the conformation previously observed in the context of the Ctf19 complex and the Ctif19-Cse4 complex. This is an interesting observation that provides potential new elements for understanding and interpreting the structural organization of the S. cerevisiae centromere.
The results are interesting, but the work needs more rigor in the experimental part, in the presentation of the data, and in the writing.

1. Electron density.
- Show the differential electron density for Cse4 when it is omitted from the model, and final electron density when it is included and refined with the model. Show also where is Cse4 density located in the crystal lattice and if crystal contact can influence interpretation.

We have included the requested electron density figures in a new Expanded View Figure. In the crystal, there are no peptides interacting with Cse4-END besides Okp1-Ame1 as shown in the main text.

- Page 2, last paragraph "Most ordered Cse4 residues in the crystal structure have a-helical character (residues 34-46), and there are short flexible extensions (residues 32-34 and 46-49)." - please illustrate with the figure in the supplementary information

This information is summarized above the sequence alignment in Figure 2A. We have simplified the statement in the updated manuscript so that the "short flexible extensions" are less of a focus, as these are just the loose ends of the Cse4-END alpha helix.

- It is very confusing to show density from an experiment done in different study in Figure 1. Please remove this from main set of figures.

We have updated the figure so that the model is a cartoon.

2. Clear analysis of the possibility of Cse4 and Nkp1/2 co-binding to Okp1-Ame1 complex.
- some of the Ame1 residues that interact with Cse4 also interact with Nkp1/2 in the Ctf19 and Ctf19-Cse4 complexes (for example, Ile195). This is not addressed in the figure or text. Figure 2, last paragraph "Nkp1 and Nkp2 bind an adjacent site in the assembled Ctf19c, but it is not clear from comparison of the cryo-EM and crystal structures (Fig. S1C) whether they would need to partly dissociate from Okp1-Ame1 to accommodate Cse4END . Aside from Nkp1 and Nkp2, the Cse4 END binding site is exposed and on an external surface in structures of the assembled Ctf19c (4, 16)." What does this mean? Would there be steric clashes between Cse4 and Nkp1/2? Figure S1C is not clear.

Please see responses above. We have moved the sentence that begins with "Nkp1 and Nkp2" as requested by Reviewer #1. Because we do not know how Nkp1-Nkp2 binds the AO complex in the "flexed head" conformation we observe in the current crystal structure, we cannot say for sure whether they will clash with Cse4-END. Nevertheless, we have updated our statements so they are clearer.

- Competition experiment with Cse4 and Nkp1/2 was done only in the context of the full length Okp1-Ame1 (that has more extensive interaction interface with Nkp1/2) and not with the constructs used in crystallography.

The competition experiment is intended to test whether Nkp1-Nkp2 can compete with Cse4, and the result shows that it cannot. It is true that we might expect to see competition between Nkp1-Nkp2 and Cse4 if we only measure binding to the observed Cse4 binding site. However, it is not clear what this would mean in terms of the underlying biology.

Also, in the context of the FL proteins, it would be nice to first form Okp1-Ame1-Nkp1/2 and then titrate in Cse4 to see if it will replace or co-bind together with Nkp1/2 or it wouldn't bind at all.

In FP experiments, Nkp1-Nkp2 are included at a vast molar excess concentration versus Cse4, which carries the tracer fluor. Therefore, given on rates governed by diffusion, the Nkp1-Nkp2-Okp1-Ame1 binding event will dominate, likely going to saturation, before Cse4-END binds appreciably. That we see no competition between Nkp1-Nkp2 and Cse4-END is, in our view, probative that there is no competition between the two. Additionally, we present a pulldown experiment (Figure 3A) that demonstrates conclusively that all three components (Okp1-Ame1, Nkp1-Nkp2, and Cse4-END) associate as a single complex with apparently equal stoichiometry.

3. Check whether the post-translational modifications identified by Anedchenko et al. affect binding. This can be easily addressed with a fluorescence polarization experiment. Also comment on their finding that Okp1-R164C suppresses the Cse4-R37A mutation.

These experiments are presented in Figure 6 of the Anedchenko manuscript, which we have cited in our manuscript. The crystal structure shows that R37 contacts the conserved Asp191 and Asp194 of Ame1. We found that mutation of Ame1-Asp191/194 caused synthetic lethality with *mcm21D* as was reported for the R37A mutation and as we state in the text.

The crystal structure shows that Okp1-Arg164 contacts the Cse4 main chain. We have added a note to describe the possible amino acid-level consequences of the R164C mutation in the updated text. It is not clear whether this change (Okp1-Arg164Cys) corresponds to biologically meaningful regulation (*i.e.* there is not data we are aware of that Okp1-Arg164 is modified in cells).

We have added a short paragraph to the discussion detailing the correspondence between the structure and these genetic observations. This paragraph includes a note about the Cse4-K49 side chain, which we cannot see in the crystal structure.

4. Pull-down experiments with GST-Cse4END should include GST only control.

We have repeated the key pulldown experiments with GST as the bait protein and present these in a new Expanded View figure.

Minor comments:
1. Abstract should have more info on the context and significance of the work.

We have added a statement to the abstract giving general context and relevance.

2. The discussion should be more organized and flow better. It is also nice if you illustrate proposed model with a cartoon. Speculate about Cnn1/Wip1 binding and compare with human CCAN structure.

We chose to keep the entire manuscript narrowly focused on the crystal structure and its implications to avoid complications arising from still incomplete understanding of inner kinetochore assembly. Please see above for a note on the requested illustration, which we now include as a supplementary figure. Because we do not understand the contribution of Cnn1/Wip1 to Cse4 nucleosome recognition (or exclusion from the Ctf19c complex), we have chosen not to discuss these factors in the current manuscript beyond the comment about Ctf3c flexibility. It is unclear what comparison with the human structure would add, especially since a functional equivalent of the Cse4-END peptide has not been found in CENP-A.

3. Figure 1A is nice, but it is not clear why are some regions in darker colors (doesn't correspond to names indicated above).

The darker colors correspond to ordered regions, which are alpha helical in the previously published cryo-EM structures. We have added a note explaining this to the legend. The names and bars below indicate the boundaries of the domains according to the cryo-EM structures. These are generally demarcated by breaks in helical segments, but this is not uniformly true (C-terminal boundary of Ame1-Head, for example).

Also, it is not very clear from the figure what are the constructs used in the study.

The amino acid boundaries for the constructs used for crystallography are indicated by the gray boxes. This is noted in the legend. In addition to this, we have removed the "Okp1^{xtal}-Ame1^{xtal}" notation from Figure EV3 and instead noted the amino acid boundaries.

4. Figures 2B and C illustrating interactions should be bigger (and Cse4 should be in the map ideally).

We have included Cse4 map images in the supplementary images. Including the map in these images (Figure 2B-C) distracts from the modeled interactions.

5. The AF2 model is OK to mention, but interpretation of the data should focus on comparison with other experimental structures.

We previously published comparisons between AlphaFold2 models and experimentally determined crystal structures (Zahm, *et al.* 2023; PMID 36883282), noting that the predictions were helpful in selecting expression constructs and in building initial models. It is also noteworthy that the AlphaFold model corresponds nicely with the extended-head AO conformation. The bulk of the comparisons do focus on the published experimental structures and the genetic data.

Also, the compatibility of the observed Okp1-Ame1 conformation with the Cse4 nucleosome within the Ctf19-Cse4 complex should be illustrated by a figure.

We don't know how Ctf19c engages the genuine Cse4 particle, as demonstrated by this work. Therefore, it is not possible to illustrate this. We have added an EV figure showing the overall arrangement of the key factors in cartoon form.

6. The authors should be aware that their results, although interesting and significant, were still obtained with truncated proteins and isolated from other components that form yeast centromeres. Therefore, they provide valuable additional information that complements previously published structures and opens the possibility of a different protein arrangement in the yeast centromere, rather than "enables us to reinterpret published models of centromere- kinetochore".

The crystallography results were obtained with truncated proteins that recapitulate in isolation an important aspect of centromere biology. The structure led to cell biology experiments, which were carried out with intact organisms, and these, along with older genetic results, are what enable us to reinterpret the published models.

7. Figures are not corresponding to the text. Page 1, last paragraph; Page 2, paragraph 5.

We have clarified or fixed these figure references.

9. Page 2, paragraph 2. "Okp1 and Ame1 are the only fully essential components of the Ctf19c." Provide reference and explain.

We have referenced the following papers, in which the *okp1-5* and *ame1-4* alleles were first reported: Ortiz *et al.* PMID 10323865 and Pot *et al.* PMID 16177574. We have also updated the wording of this sentence so that instead of "essential," it says, "required for mitosis."

10. Page 4, paragraph 2. Fig. 5A should be Fig 4A.

We have fixed this mistake.

11. All statements based on previous data should contain corresponding references.

We have added references as requested.

Referee #3:

The kinetochore is a large protein complex, which mediates accurate chromosome segregation through bridging centromeric chromatin with spindle microtubules. Centromeric chromatin contains a special nucleosome including Cse4p (Budding yeast) or CENP-A (human), which are centromere specific-histone H3 variant. Therefore, it is important for kinetochore components to distinguish centromeric chromatin from canonical H3 containing nucleosomes. Authors in this MS used budding yeast kinetochore components and tried to address how they specifically recognize Cse4p. They focused on the Cse4p binding to the Okp1-Ame1 complex and determined a crystal structure of the Okp1-Ame1 complex bound with Cse4p N-terminal peptide. They found that Cse4p N-terminal peptide bound a junction of Head domain and coiled-coil of the Okp1-Ame1 complex. They further identified several critical amino acids for robust binding of Cse4p to the Okp1-Ame1 complex. Mutation of these amino acids resulted in binding defects of Cse4p to the Okp1-Ame1 complex in vitro and caused cell death in vivo. Then, authors proposed that this binding is a key initial step for kinetochore assembly on the Cse4p containing centromeric chromatin.
Recent years, a cryo-EM structure of the entire kinetochore complex including Cse4p and the Okp1-Ame1 complex has been solved. However, based on the Cryo-EM structure, it was unclear how the kinetochore complex such as the Okp1-Ame1 complex recognizes the Cse4 nucleosome, because clear binding of Cse4p to the Okp1-Ame1 complex was not observed in the cryo-EM structure. However, as binding of Cse4p to the Okp1-Ame1 complex was observed in vitro experiments, it was open question to address how the kinetochore complex specifically recognizes Cse4p from canonical H3 nucleosomes. Authors tackled this question in this paper. Data quality in this MS is relatively high and this reviewer notices that this kind of piece of experiment is important to complement the cryo-EM structure. Therefore, I basically support the publication of this work in EMBO Rep. However, I have some suggestions to improve the paper, and authors should consider for incorporation of my suggestions in the revised version.

1. Concerning Nkp1-2, authors mentioned that the Cse4-Ame-Okp1-Nkp1-2 complex can be formed (Figure 3). Authors also described that Cse4 binding affinity to the Ame1-Okp1 complex to similar even in the absence of the Nkp1-2 complex. However, in Figure 3 experiments biding affinity seems to be slightly decreased in the absence of the Nkp1-2 complex (Figure 3B). I think that they can show more quantitative presentation for binding in either presence or absence of the Nkp1-2 complex. KDs for these bindings can be presented in either presence or absence of the Nkp1-2 complex.

The quantification of the binding experiments in Figure 3B is in Table 1. The binding curves showing this, which are presented in Figure 3B, have been repeated. In the initial submission, a plate reader normalization setting caused the curves to be offset on the Y-axis, but this has now been fixed. The original result has been confirmed. Quantification of the binding equilibria shows that inclusion of Nkp1/2 does not change the measured affinity of Okp1-Ame1 for Cse4-END.

2. For in vivo experiment, it might be better to add data for Cse4 lacking END sequence. We can easily compare phenotype of Cse4-2A or 1A and Cse4 END deletion.

The Cse4-END deletion was previously reported as lethal by the Fitzgerald-Hayes lab (PMID 10958698), the Kaufman lab (PMID 30136924, complete deletion of Cse4-1-70), and the Herzog lab (PMID 31112132, anchor away experiment). The updated manuscript includes citations of all three papers in the relevant spots. We have also included diagrams of the key alleles in an updated Figure EV5.

3. In this paper authors used Cse4 peptide, but I am curious to know whether Cse4 N-terminal region binds to the Ame-Okp1 complex in the nucleosome context. If authors show binding results using Cse4 Nucleosome, it would be better.

We do not know how the Ctf19c binds the Cse4 nucleosome, nor do we know what kind of nucleosome it binds. Our work shows that the published Cse4-Ctf19c structure(s) cannot easily be accepted as correct recapitulations of the situation *in vivo*, and our own unpublished efforts to reconstitute such a complex have resulted in similarly impossible structures. Indeed, faithfully reconstituting a meaningful Ctf19c-Cse4 interaction is a major outstanding objective, and the work we have presented here is a step in this direction.

To wit:
I am curious to know whether Cse4 N-terminal region binds to the Ame-Okp1 complex in the nucleosome context.
So are we! Figuring this out would be a major advance.

4. In discussion, it would be good to discuss the Mif2 pathway. Cse4A-1A mutation showed slow growth, but cells with this mutation were still viable. In vitro experiments in Figure 3C indicated that both 1A and 2A mutations did not binds to the Okp1-Ame1 complex ar similar extent. Considering these results, we suggest that there would be the redundant pathway to link the kinetochore with centromeric chromatin in vivo. Mif2 is a good candidate to link the kinetochore with centromeric chromatin. It would be good to discuss this point.

Figure 3C does not show the *cse4-1A* mutation (only -2A). It is true that the hypomorphic phenotype of the -1A mutation could provide a useful tool for studying synthetic interactions in the yeast kinetochore that disable related foundational contacts, but deciphering the biochemical and genetic interactions (and their regulation) is beyond the scope of this work. Indeed, in our work on Mif2, we have found that seemingly simple phosphoregulation of Mif2 has complex consequences; genetic and biochemical interactions are strengthened and weakened in unexpected ways in the corresponding mutants (Hinshaw et al. 2023; PMID 36736323). We think this indicates layered negative and positive regulation during cell cycle progression. We have made more explicit mention of Mif2 in the final sentence of the discussion and include it in the updated Figure 1B cartoon.

Minor points
In page 4, for Figure describing CSE4 mutation, authors indicated Fig5A. It should be Fig 4A.

We have fixed this mistake.

Dear Dr. Hinshaw,

Thank you for submitting your revised manuscript. It has now been seen by all of the original referees.

As you can see, the referees find that the study is significantly improved during revision and recommend publication. However, I need you to address the points below before I can accept the manuscript.

• Please address the remaining concerns of referee #2 by expanding the discussion on Dendoeven et al. (PMID: 37506202), focusing on direct comparison of the structures presented.
• Please provide 3-5 keywords for your study. These will be visible in the html version of the paper and on PubMed and will help increase the discoverability of your work.
• Please remove the Author Contribution section from the manuscript text.
• As per our format requirements, in the reference list, citations should be listed in alphabetical order and then chronologically, with the authors' surnames and initials inverted; where there are more than 10 authors on a paper, 10 will be listed, followed by 'et al.'. Moreover, we need DOIs only for preprints and datasets that have not been published yet. Please see https://www.embopress.org/page/journal/14693178/authorguide#referencesformat
• We note that the funding information is not complete in the manuscript submission system (Argonne National Laboratory under Contract DE-AC02-06CH11357 is missing).
• Please remove the figures from the manuscript, but leave the legends in.
• We note that Figure 1B is currently not called out in the text.
• Tables EV1-EV3 need to be removed from the manuscript and uploaded separately.
• Please upload source data as one file per figure. Currently, source data for Figures 1 and 2 are combined as one file.
• The manuscript sections should be in the following order: Title page - Abstract & Keywords - Introduction - Results - Discussion - Materials & Methods - Data Availability - Acknowledgments - Disclosure Statement & Competing Interests - References - Figure Legends - Tables with legends - Expanded View Figure Legends.
• Our data editors have asked you to clarify the below points in the figure legends:

o Please note that 'Figure Title' for all main figures 1-4 are not provided.
o Please note that the figure legend style does not comply with the journal guidelines i.e. all the figure legends are in a run-on style. Instead, figure legends should be formatted such that each panel, or group of panels, has its own entry with the panel letter (or range) on the left and the description on the right, the panels should be described in sequential order in the legend. Please see https://www.embopress.org/page/journal/14693178/authorguide#figureformat

• Papers published in EMBO Reports include a 'synopsis' and 'bullet points' to further enhance discoverability. Both are displayed on the html version of the paper and are freely accessible to all readers. The synopsis includes a short standfirst summarizing the study in 1 or 2 sentences (max 35 words) that summarize the paper and are provided by the authors and streamlined by the handling editor. I would therefore ask you to include your synopsis blurb and 3-5 bullet points listing the key experimental findings.
• In addition, please provide an image for the synopsis. This image should provide a rapid overview of the question addressed in the study but still needs to be kept fairly modest since the image size cannot exceed 550 (width) x 300-600 (height) pixels.

Thank you again for giving us to consider your manuscript for EMBO Reports, I look forward to your minor revision.

Kind regards,

Deniz Senyilmaz Tiebe

--
Deniz Senyilmaz Tiebe, PhD
Editor
EMBO Reports

Referee #1:

I am grateful to the authors for submitting this improved manuscript and am delighted to support its publication in EMBO Reports. Congratulations to the authors for an important new achievement.

Referee #2:

While this manuscript was under review, a cryo EM structure of the complete inner kinetochore of the budding yeast point centromere was published by Dendooven at al. (PMID: 37506202). Their work, among others, also addresses the interactions of the N-terminal Cse4 with Ame1 and Opk1 and presents a different type of interaction between these proteins in the context of the complete inner kinetochore. Although the authors of the manuscript under review cite the new publication in their revised manuscript, they do not directly compare their result with that in the publication or offer a convincing interpretation of the differences. Moreover, a clear discussion of possible steric conflicts between Nkp1/Nkp2 and the N-term of Cse4, as modeled on the previous cryoEM structure without Cse4, is still missing (although 2 reviewers asked for this). Interestingly, Dendooven at al. found that the Nkp1/Nkp2 complex undergoes a conformational change when in the complete inner kinetochore context, and this exposes the binding site for the N-terminal Cse4 at Ame1/Opk1. The authors here failed to provide a transparent comparison of the different binding modes and the relationship between Nkp1/Nkp2, N-terminal Cse4, and Ame1/Opk1 in different structures. This is a pity, because despite the new publication (or earlier publications to that effect), they would have had the chance to put their work in a broader context and explain how/why their structure still provides important information without disapproving other models. It is possible that complexes undergo conformational changes and reorganizations during the cell cycle and more than one arrangement is possible and relevant.

Referee #3:

This is a revised MS for interaction of Cse4 N-terminus with the Okp1-Ame1 complex by Hinshaw and his colleagues. I think that authors addressed all concerns from reviewers including myself, and the MS is improved well. Therefore, I support publication of this MS in EMBO Rep.

Follow-up to Editorial Decision — 27th Oct 2023

Dear Stephen,

I would like to add another point to the decision letter below. Your manuscript was submitted as 'Research Article'. Since your manuscript fits better into our 'Report' article type given the number of figures and the word count, I would like to encourage you to change the article type accordingly during revision. If you agree with this, please combine Results and Discussion sections into one section (as explained here https://www.embopress.org/page/journal/14693178/authorguide#researcharticleguide). Thank you.

Kind regards,

Deniz

--

Dear Dr. Hinshaw,

Thank you for submitting your revised manuscript. It has now been seen by all of the original referees.

As you can see, the referees find that the study is significantly improved during revision and recommend publication. However, I need you to address the points below before I can accept the manuscript.

• Please address the remaining concerns of referee #2 by expanding the discussion on Dendoeven et al. (PMID: 37506202), focusing on direct comparison of the structures presented.

We now directly address the Dendoeven work to the manuscript, and we have responded in-line to the reviewer's comments below.

• Please provide 3-5 keywords for your study. These will be visible in the html version of the paper and on PubMed and will help increase the discoverability of your work.

We have included these in the manuscript file.

• Please remove the Author Contribution section from the manuscript text.

Done.

• As per our format requirements, in the reference list, citations should be listed in alphabetical order and then chronologically, with the authors' surnames and initials inverted; where there are more than 10 authors on a paper, 10 will be listed, followed by 'et al.'. Moreover, we need DOIs only for preprints and datasets that have not been published yet. Please see https://www.embopress.org/page/journal/14693178/authorguide#referencesformat

Done.

• We note that the funding information is not complete in the manuscript submission system (Argonne National Laboratory under Contract DE-AC02-06CH11357 is missing).

We added this in the submission system.

• Please remove the figures from the manuscript, but leave the legends in.

Done.

• We note that Figure 1B is currently not called out in the text.

We added a reference to Figure 1B as requested.

• Tables EV1-EV3 need to be removed from the manuscript and uploaded separately.

Done.

• Please upload source data as one file per figure. Currently, source data for Figures 1 and 2 are combined as one file.

Done.

• The manuscript sections should be in the following order: Title page - Abstract & Keywords - Introduction - Results - Discussion - Materials & Methods - Data Availability - Acknowledgments - Disclosure Statement & Competing Interests - References - Figure Legends - Tables with legends - Expanded View Figure Legends.

Done.

• Our data editors have asked you to clarify the below points in the figure legends:

Done.

o Please note that 'Figure Title' for all main figures 1-4 are not provided.

o Please note that the figure legend style does not comply with the journal guidelines i.e. all the figure legends are in a run-on style. Instead, figure legends should be formatted such that each panel, or group of panels, has its own entry with the panel letter (or range) on the left and the description on the right, the panels should be described in sequential order in the legend. Please see https://www.embopress.org/page/journal/14693178/authorguide#figureformat

• Papers published in EMBO Reports include a 'synopsis' and 'bullet points' to further enhance discoverability. Both are displayed on the html version of the paper and are freely accessible to all readers. The synopsis includes a short standfirst summarizing the study in 1 or 2 sentences (max 35 words) that summarize the paper and are provided by the authors and streamlined by the handling editor. I would therefore ask you to include your synopsis blurb and 3-5 bullet points listing the key experimental findings.

We have added the requested items as an additional file.

• In addition, please provide an image for the synopsis. This image should provide a rapid overview of the question addressed in the study but still needs to be kept fairly modest since the image size cannot exceed 550 (width) x 300-600 (height) pixels.

The requested image is included as a submitted file.

Thank you again for giving us to consider your manuscript for EMBO Reports, I look forward to your minor revision.

Kind regards,

Deniz Senyilmaz Tiebe

--
Deniz Senyilmaz Tiebe, PhD
Editor
EMBO Reports

Referee #1:

I am grateful to the authors for submitting this improved manuscript and am delighted to support its publication in EMBO Reports. Congratulations to the authors for an important new achievement.

Referee #2:

While this manuscript was under review, a cryo EM structure of the complete inner kinetochore of the budding yeast point centromere was published by Dendooven at al. (PMID: 37506202). Their work, among others, also addresses the interactions of the N-terminal Cse4 with Ame1 and Opk1 and presents a different type of interaction between these proteins in the context of the complete inner kinetochore.

Dendooven et al. do not resolve Cse4-END in their cryo-EM map showing nearly all components of the inner kinetochore. The only Cse4-END structure reported in the Dendooven manuscript is from Alpha-Fold. We note the correctness of this predictions and commend the nice biochemical experiments testing this model.

Although the authors of the manuscript under review cite the new publication in their revised manuscript, they do not directly compare their result with that in the publication or offer a convincing interpretation of the differences.

Direct comparison is difficult for two reasons. First, Dendooven et al. do not report a structure of Cse4-END in the context of their assembled particle. Second, with respect to Cse4-Okp1-Ame1, the reported complete assembly does not go beyond what has been reported previously. One specific comparison we do make in the manuscript is that Dendooven et al. find that Cse4-END does not associate biochemically with their assembled particle.

Moreover, a clear discussion of possible steric conflicts between Nkp1/Nkp2 and the N-term of Cse4, as modeled on the previous cryoEM structure without Cse4, is still missing (although 2 reviewers asked for this).

Our previous manuscript version included this sentence: “Because the Okp1-Ame1 head domain is shifted in the crystal structure as described above, it is not clear from these comparisons whether Nkp1-Nkp2 would need to partly dissociate from Okp1-Ame1 to accommodate $Cse4^{END}$ (Figure EV1F).”

We include the following sentence in the updated manuscript: "Indeed, a recent cryo-EM structure of COMA-Nkp1-Nkp2 shows partial Nkp1-Nkp2 dissociation from Okp1-Ame1 is possible (Dendooven et al., 2023)."

Interestingly, Dendooven at al. found that the Nkp1/Nkp2 complex undergoes a conformational change when in the complete inner kinetochore context, and this exposes the binding site for the N-terminal Cse4 at Ame1/Opk1.

This is incorrect. The Nkp1/2 conformation change is only observed in a cryo-EM structure of COMA-Nkp1-Nkp2 and not in the full inner kinetochore structure (as it is reported). In the full complex Nkp1/2 is in the "default" (previously observed) position, which is presumed to be incompatible with Cse4-END binding.

The authors here failed to provide a transparent comparison of the different binding modes and the relationship between Nkp1/Nkp2, N-terminal Cse4, and Ame1/Opk1 in different structures.

It's unclear what different binding modes there are to compare. We have added a note on the observed partial dissociation of Nkp1-Nkp2 to the results section.

This is a pity, because despite the new publication (or earlier publications to that effect), they would have had the chance to put their work in a broader context and explain how/why their structure still provides important information without disapproving other models.

We think the work as presented does exactly this. Our crystal structure enables evaluation of previously proposed kinetochore assembly states and helps us understand genetic literature pertaining to heretofore confusing *cse4* alleles. We are careful to avoid stating we have disproved any models. Instead, we discuss how our experiments influence our thinking about the big picture and how the various proposed assembly states are likely to fit into the puzzle.

It is possible that complexes undergo conformational changes and reorganizations during the cell cycle and more than one arrangement is possible and relevant.

We agree with this statement, and the manuscript is written from this perspective. We add the following: results of structural biology experiments should be regarded with suspicion until incisive experiments can be carried out to contextualize and evaluate the findings. This principle may be a minor concern for crystal structures of isolated protein domains and situations where previous literature points to the essential correctness of the structural findings. By contrast, this principle applies *writ large* for massive DNA-bound complexes subject to extensive post-translational regulation and with shifting functions during the cell cycle, the likes of which are now amenable to cryo-EM.

Referee #3:

This is a revised MS for interaction of Cse4 N-terminus with the Okp1-Ame1 complex by Hinshaw and his colleagues. I think that authors addressed all concerns from reviewers including myself, and the MS is improved well. Therefore, I support publication of this MS in EMBO Rep.

Dear Stephen,

Thank you for submitting your revised manuscript. I have now looked at everything and all is fine. Therefore, I am very pleased to accept your manuscript for publication in EMBO Reports.

Congratulations on a nice work!

Kind regards,

Deniz
--
Deniz Senyilmaz Tiebe, PhD
Editor
EMBO Reports

--

At the end of this email I include important information about how to proceed. Please ensure that you take the time to read the information and complete and return the necessary forms to allow us to publish your manuscript as quickly as possible.

As part of the EMBO publication's Transparent Editorial Process, EMBO reports publishes online a Review Process File to accompany accepted manuscripts. As you are aware, this File will be published in conjunction with your paper and will include the referee reports, your point-by-point response and all pertinent correspondence relating to the manuscript.

If you do NOT want this File to be published, please inform the editorial office within 2 days, if you have not done so already, otherwise the File will be published by default [contact: emboreports@embo.org]. If you do opt out, the Review Process File link will point to the following statement: "No Review Process File is available with this article, as the authors have chosen not to make the review process public in this case."

Should you be planning a Press Release on your article, please get in contact with emboreports@wiley.com as early as possible, in order to coordinate publication and release dates.

Thank you again for your contribution to EMBO reports and congratulations on a successful publication. Please consider us again in the future for your most exciting work.

****

THINGS TO DO NOW:

Please note that you will be contacted by Wiley Author Services to complete licensing and payment information. The required 'Page Charges Authorization Form' is available here: https://www.embopress.org/pb-assets/embo-site/er_apc.pdf - please download and complete the form and return to embopressproduction@wiley.com

EMBO Press participates in many Publish and Read agreements that allow authors to publish Open Access with reduced/no publication charges. Check your eligibility: https://authorservices.wiley.com/author-resources/Journal-Authors/open-access/affiliation-policies-payments/index.html

You will receive proofs by e-mail approximately 2-3 weeks after all relevant files have been sent to our Production Office; you should return your corrections within 2 days of receiving the proofs.

Please inform us if there is likely to be any difficulty in reaching you at the above address at that time. Failure to meet our deadlines may result in a delay of publication, or publication without your corrections.

All further communications concerning your paper should quote reference number EMBOR-2023-57702V3 and be addressed to emboreports@wiley.com.

Should you be planning a Press Release on your article, please get in contact with emboreports@wiley.com as early as possible, in order to coordinate publication and release dates.

EMBO Press Author Checklist

Corresponding Author Name: Stephen M Hinshaw	
Journal Submitted to: EMBO Reports	
Manuscript Number: EMBOR-2023-57702	

USEFUL LINKS FOR COMPLETING THIS FORM
The EMBO Journal - Author Guidelines
EMBO Reports - Author Guidelines
Molecular Systems Biology - Author Guidelines
EMBO Molecular Medicine - Author Guidelines

Reporting Checklist for Life Science Articles (updated January
This checklist is adapted from Materials Design Analysis Reporting (MDAR) Checklist for Authors. MDAR establishes a minimum set of requirements in transparent reporting in the life sciences (see Statement of Task: 10.31222/osf.io/9sm4x). Please follow the journal's guidelines in preparing your
Please note that a copy of this checklist will be published alongside your article.

Abridged guidelines for figures
1. Data
The data shown in figures should satisfy the following conditions:
- the data were obtained and processed according to the field's best practice and are presented to reflect the results of the experiments in an accurate and unbiased manner.
- ideally, figure panels should include only measurements that are directly comparable to each other and obtained with the same assay.
- plots include clearly labeled error bars for independent experiments and sample sizes. Unless justified, error bars should not be shown for technical
- if n<5, the individual data points from each experiment should be plotted. Any statistical test employed should be justified.
- Source Data should be included to report the data underlying figures according to the guidelines set out in the authorship guidelines on Data

2. Captions
Each figure caption should contain the following information, for each panel where they are relevant:
- a specification of the experimental system investigated (eg cell line, species name).
- the assay(s) and method(s) used to carry out the reported observations and measurements.
- an explicit mention of the biological and chemical entity(ies) that are being measured.
- an explicit mention of the biological and chemical entity(ies) that are altered/varied/perturbed in a controlled manner.
- the exact sample size (n) for each experimental group/condition, given as a number, not a range;
- a description of the sample collection allowing the reader to understand whether the samples represent technical or biological replicates (including how many animals, litters, cultures, etc.).
- a statement of how many times the experiment shown was independently replicated in the laboratory.
- definitions of statistical methods and measures:
  - common tests, such as t-test (please specify whether paired vs. unpaired), simple χ2 tests, Wilcoxon and Mann-Whitney tests, can be unambiguously identified by name only, but more complex techniques should be described in the methods section;
  - are tests one-sided or two-sided?
  - are there adjustments for multiple comparisons?
  - exact statistical test results, e.g., P values = x but not P values < x;
  - definition of 'center values' as median or average;
  - definition of error bars as s.d. or s.e.m.

Please complete ALL of the questions below.
Select "Not Applicable" only when the requested information is not relevant for your study.

Materials

Newly Created Materials	Information included in the manuscript?	In which section is the information available? (Reagents and Tools Table, Materials and Methods, Figures, Data Availability Section)
New materials and reagents need to be available; do any restrictions apply?	Yes	Table S3

Antibodies	Information included in the manuscript?	In which section is the information available? (Reagents and Tools Table, Materials and Methods, Figures, Data Availability Section)
For **antibodies** provide the following information:<br>- Commercial antibodies: RRID (if possible) or supplier name, catalogue number and or/clone number<br>- Non-commercial: RRID or citation	Not Applicable	N/A

DNA and RNA sequences	Information included in the manuscript?	In which section is the information available? (Reagents and Tools Table, Materials and Methods, Figures, Data Availability Section)
**Short novel DNA or RNA including primers, probes**: provide the sequences.	Not Applicable	

Cell materials	Information included in the manuscript?	In which section is the information available? (Reagents and Tools Table, Materials and Methods, Figures, Data Availability Section)
**Cell lines:** Provide species information, strain. Provide accession number in repository **OR** supplier name, catalog number, clone number, and/**OR** RRID.	Yes	Table S2
**Primary cultures:** Provide species, strain, sex of origin, genetic modification status.	Not Applicable	
Report if the cell lines were recently **authenticated** (e.g., by STR profiling) and tested for mycoplasma contamination.	Not Applicable	

Experimental animals	Information included in the manuscript?	In which section is the information available? (Reagents and Tools Table, Materials and Methods, Figures, Data Availability Section)
**Laboratory animals or Model organisms:** Provide species, strain, sex, age, genetic modification status. Provide accession number in repository **OR** supplier name, catalog number, clone number, **OR** RRID.	Not Applicable	
**Animal observed in or captured from the field:** Provide species, sex, and age where possible.	Not Applicable	
Please detail **housing and husbandry conditions**.	Not Applicable	

Plants and microbes	Information included in the manuscript?	In which section is the information available? (Reagents and Tools Table, Materials and Methods, Figures, Data Availability Section)
**Plants:** provide species and strain, ecotype and cultivar where relevant, unique accession number if available, and source (including location for collected wild specimens).	Not Applicable	
**Microbes:** provide species and strain, unique accession number if available, and source.	Not Applicable	

Human research participants	Information included in the manuscript?	In which section is the information available? (Reagents and Tools Table, Materials and Methods, Figures, Data Availability Section)
If collected and within the bounds of privacy constraints report on age, sex and gender or ethnicity for all study participants.	Not Applicable	

Core facilities	Information included in the manuscript?	In which section is the information available? (Reagents and Tools Table, Materials and Methods, Figures, Data Availability Section)
If your work benefited from core facilities, was their service mentioned in the acknowledgments section?	Yes	Methods, and Acknowledgement

Design

Study protocol	Information included in the manuscript?	In which section is the information available? (Reagents and Tools Table, Materials and Methods, Figures, Data Availability Section)

If study protocol has been **pre-registered, provide DOI in the manuscript**. For clinical trials, provide the trial registration number **OR** cite DOI.	Not Applicable	
Report the **clinical trial registration number** (at ClinicalTrials.gov or equivalent), where applicable.	Not Applicable	

Laboratory protocol	Information included in the manuscript?	In which section is the information available? (Reagents and Tools Table, Materials and Methods, Figures, Data Availability Section)
Provide DOI OR other citation details if **external detailed step-by-step protocols** are available.	Not Applicable	

Experimental study design and statistics	Information included in the manuscript?	In which section is the information available? (Reagents and Tools Table, Materials and Methods, Figures, Data Availability Section)
Include a statement about **sample size** estimate even if no statistical methods were used.	Not Applicable	
Were any steps taken to minimize the effects of subjective bias when allocating animals/samples to treatment (e.g. **randomization procedure**)? If yes, have they been described?	Not Applicable	
Include a statement about **blinding** even if no blinding was done.	Not Applicable	
Describe **inclusion/exclusion criteria** if samples or animals were excluded from the analysis. Were the criteria pre-established?<br><br>If sample or data points were omitted from analysis, report if this was due to attrition or intentional exclusion and provide justification.	Not Applicable	
For every figure, are **statistical tests** justified as appropriate? Do the data meet the assumptions of the tests (e.g., normal distribution)? Describe any methods used to assess it. Is there an estimate of variation within each group of data? Is the variance similar between the groups that are being statistically compared?	Not Applicable	

Sample definition and in-laboratory replication	Information included in the manuscript?	In which section is the information available? (Reagents and Tools Table, Materials and Methods, Figures, Data Availability Section)
In the figure legends: state number of times the experiment was **replicated** in laboratory.	Yes	Figure 3B, Figure EV3C
In the figure legends: define whether data describe **technical or biological replicates**.	Yes	Figure 3B, Figure EV3C

Ethics

Ethics	Information included in the manuscript?	In which section is the information available? (Reagents and Tools Table, Materials and Methods, Figures, Data Availability Section)
Studies involving **human participants**: State details of **authority granting ethics approval** (IRB or equivalent committee(s), provide reference number for approval.	Not Applicable	
Studies involving **human participants**: Include a statement confirming that **informed consent** was obtained from all subjects and that the experiments conformed to the principles set out in the WMA Declaration of Helsinki and the Department of Health and Human Services Belmont Report.	Not Applicable	
Studies involving **human participants:** For publication of **patient photos**, include a statement confirming that consent to publish was obtained.	Not Applicable	
Studies involving experimental **animals**: State details of **authority granting ethics approval** (IRB or equivalent committee(s), provide reference number for approval. Include a statement of compliance with ethical regulations.	Not Applicable	
Studies involving **specimen and field samples:** State if relevant **permits** obtained, provide details of authority approving study; if none were required, explain why.	Not Applicable	

Dual Use Research of Concern (DURC)	Information included in the manuscript?	In which section is the information available? (Reagents and Tools Table, Materials and Methods, Figures, Data Availability Section)
Could your study fall under dual use research restrictions? Please check biosecurity documents and list of **select agents and toxins** (CDC): https://www.selectagents.gov/sat/list.htm	Not Applicable	
If you used a select agent, is the security level of the lab appropriate and reported in the manuscript?	Not Applicable	
If a study is subject to dual use research of concern regulations, is the name of the **authority granting approval and reference number** for the regulatory approval provided in the manuscript?	Not Applicable	

Reporting

The MDAR framework recommends adoption of discipline-specific guidelines, established and endorsed through community initiatives. Journals have their own policy about requiring specific guidelines and recommendations to complement MDAR.

Adherence to community standards	Information included in the manuscript?	In which section is the information available? (Reagents and Tools Table, Materials and Methods, Figures, Data Availability Section)
State if relevant guidelines or checklists (e.g., **ICMJE, MIBBI, ARRIVE, PRISMA**) have been followed or provided.	Not Applicable	
For **tumor marker prognostic studies,** we recommend that you follow the **REMARK** reporting guidelines (see link list at top right). See author guidelines, under 'Reporting Guidelines'. Please confirm you have followed these guidelines.	Not Applicable	
For **phase II and III randomized controlled trials**, please refer to the **CONSORT** flow diagram (see link list at top right) and submit the CONSORT checklist (see link list at top right) with your submission. See author guidelines, under 'Reporting Guidelines'. Please confirm you have submitted this list.	Not Applicable	

Data Availability

Data availability	Information included in the manuscript?	In which section is the information available? (Reagents and Tools Table, Materials and Methods, Figures, Data Availability Section)
Have **primary datasets** been deposited according to the journal's guidelines (see 'Data Deposition' section) and the respective accession numbers provided in the Data Availability Section?	Yes	Data availability
Were **human clinical and genomic datasets** deposited in a public access-controlled repository in accordance to ethical obligations to the patients and to the applicable consent agreement?	Not Applicable	
Are **computational models** that are central and integral to a study available without restrictions in a machine-readable form? Were the relevant accession numbers or links provided?	Not Applicable	
If publicly available data were reused, provide the respective **data citations in the reference list**.	Not Applicable	
